# Unveiling the nexus between irradiation and phase reconstruction in tin-lead perovskite solar cells

**DOI:** 10.1038/s41467-025-55814-0

**Published:** 2025-01-08

**Authors:** Wenbo Li, Zhe Li, Shun Zhou, Yanzhuo Gou, Guang Li, Jinghao Li, Cheng Wang, Yan Zeng, Jiakai Yan, Yan Li, Wei Dai, Yaoguang Rong, Weijun Ke, Ti Wang, Hongxing Xu

**Affiliations:** 1 School of Physics and Technology, and Key Laboratory of Artificial Micro- and Nano-structures of Ministry of Education, Wuhan University, Wuhan, China; 2 School of Materials Science and Engineering, Hubei University, Wuhan, China; 3 State Key Laboratory of Advanced Technology for Materials Synthesis and Processing, Wuhan University of Technology, Wuhan, China; 4 School of Microelectronics, Wuhan University, Wuhan, China; 5 Wuhan Institute of Quantum Technology, Wuhan, China; 6 Henan Academy of Sciences, Zhengzhou, China

**Keywords:** Solar cells, Solar cells

## Abstract

Tin-lead perovskites provide an ideal bandgap for narrow-bandgap perovskites in all-perovskite tandem solar cells, fundamentally improving power conversion efficiency. However, light-induced degradation in ambient air is a major issue that can hinder the long-term operational stability of these devices. Understanding the specifics of what occurs during this pathway provides the direction for improving device stability. In this study, we investigate the long-term stability problem of tin-lead perovskites under irradiation, counterintuitively discovering an irreversible phase reconstruction process. In-situ photoluminescence spectroscopy is used to monitor the reconstruction process, which involves the reaction of oxygen with photoexcited electrons to form superoxide. It is proposed that Pb-rich regions appear on the surface after Sn^2+^ oxidation, and these Pb-rich regions are reconstituted from the yellow phase of formamidinium lead iodide to the black phase with prolonged irradiation. This study highlights the phase reconstruction process during the degradation of tin-lead perovskites, providing valuable insights into the superoxide degradation mechanism and guiding further stability improvements for narrow-bandgap tin-lead perovskites and tandem solar cells.

## Abstract (web summary)

Light-induced degradation in ambient air hinders the long-term operational stability of tin-lead perovskite solar cells. Here, authors monitor the irreversible phase reconstruction process during perovskite degradation, guiding stability improvements for tin-lead perovskite and tandem solar cells.

## Introduction

The power conversion efficiency (PCE) of single-junction solar cells is inherently limited by the principle of detailed balance. To surpass this constraint, a tandem cell architecture can be utilized^1–5^. This design incorporates a top cell with a wider bandgap to absorb high-energy photons, yielding a higher open-circuit voltage, while the bottom cell with a narrower bandgap captures the remaining low-energy photons^6–8^. Compared to silicon-perovskite tandem cells, all-perovskite tandem cells provide significant advantages, such as lower processing temperatures and greater compositional tunability, which are beneficial for manufacturing processes^8,9^. Mixed tin-lead perovskites, known for their strong bandgap bowing effect, achieve the narrowest possible bandgap among perovskites^10,11^. This makes them highly promising candidates for the bottom subcell in an all-perovskite tandem configuration^12–15^.

For successful commercialization, achieving high PCE is essential, but long-term light stability is equally crucial^3,16^. Although all-perovskite tandem solar cells have achieved a certified PCE of over 30%, their stability remains insufficient for commercial viability. This limitation is mainly due to the instability of their bottom subcells, which use narrow-bandgap perovskites incorporating Sn elements. The Sn elements in narrow-bandgap perovskites are unstable in the form of stannous (Sn^2+^), which can easily oxidize to Sn^4+^. The formation of Sn^4+^ accelerates nonradiative recombination, thus deteriorating device performance and hampering commercial viability^17–20^. Even under encapsulated conditions, these devices remain unstable when exposed to light and oxygen, adversely impacting their optoelectronic performance^21^. The degradation mechanism of tin-lead perovskites is a significant area of ongoing research. Studies have clarified the degradation processes of pure tin-based perovskites under moisture, heat, and light conditions. Specifically, in the absence of light, degradation by oxygen (O_2_) molecules is relatively mild. However, under light exposure, photo-excited electrons are captured by O_2_ molecules to form superoxide (O_2_^−^), which disrupts the covalent bond of Sn-I^22,23^. This photo-activation of electrons leads to the formation of superoxide from O_2_, accelerating the oxidation of Sn^2+^ and the generation of I^−^ vacancies, thereby causing a significant drop in device performance. Superoxide-induced degradation is prevalent in various perovskites and tends to occur more rapidly than moisture-induced degradation^24^. The formation of I^−^ and Sn^2+^ vacancies can lead to irreversible changes in the perovskite crystalline regions. Although the degradation pathways of pure tin-based perovskites have been extensively studied, the degradation processes in tin and lead alloyed perovskites may differ significantly^18,25–27^. Therefore, it is crucial to deeply investigate the degradation mechanisms of tin-lead perovskites under light and ambient air conditions. This understanding is fundamental for improving the stability of all-perovskite tandem solar cells, which remains a significant challenge for their future commercialization and practical application^28–31^.

Here, we reveal that irradiation of tin-lead perovskite films in ambient air initiates a superoxidation process, leading to phase reconstructions that produce Pb-rich regions. The oxidation of Sn^2+^ generates a large number of vacancy defects, and the tin-lead mixed region undergoes phase reconstructions to produce formamidinium lead iodide (FAPbI_3_), as documented by in-situ photoluminescence (PL) spectroscopy. Specifically, FAPbI_3_ in the Pb-rich region transitions from a yellow to a black phase with prolonged irradiation. Triiodide ions are also formed during this process, consistent with the superoxidation observed in tin-based perovskite. Additionally, the air-aged film retains the black phase of FAPbI_3_ when irradiated in air, due to the entry of superoxide ions. These processes are individual to mixed tin-lead perovskites and have not been observed in pure lead or tin-based perovskites. Our work systematically monitors the structural and compositional evolution of tin-lead perovskite reconstructed in phase under irradiation in ambient air and provides essential insights into their impact on the long-term stability of all-perovskite tandem electronic devices.

## Results

### Phase reconstruction in tin-lead perovskite devices

**Fig. 1:**
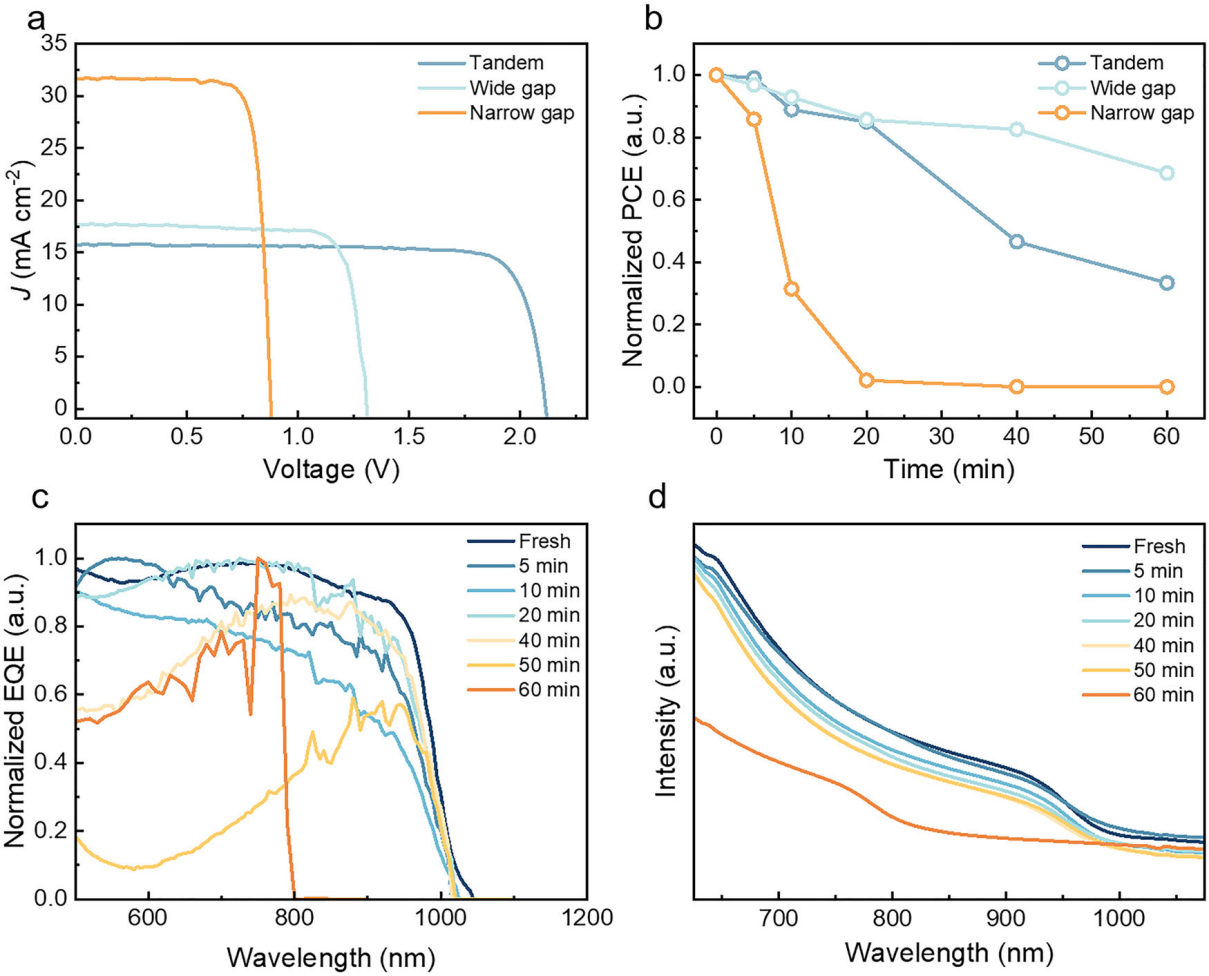
Wide bandgap perovskite single-junction, narrow bandgap perovskite single-junction and 2T all-perovskite tandem PSCs. **a** Typical photocurrent density-voltage (*J–V*) curves of single-junction wide bandgap perovskite, single-junction narrow bandgap perovskite, and 2T all-perovskite tandem solar cells. **b** Irradiation/air stability tests for unencapsulated single-junction wide bandgap perovskite, single-junction narrow bandgap perovskite, and 2T all-perovskite tandem solar cells. **c** External quantum efficiency spectra of a single-junction narrow bandgap perovskite solar cell under irradiation/air exposure. **d** Absorption spectra of a narrow bandgap perovskite thin film under irradiation/air exposure.

The perovskite solar cells (PSCs) were synthesized by spin-coating perovskite precursors, as detailed in the Methods section. Figure 1a presents the photocurrent density-voltage (*J*–*V*) curves of a representative single-junction wide bandgap (FA_0.8_Cs_0.2_PbI_1.8_Br_1.2_) perovskite, a representative single-junction narrow bandgap (FA_0.7_MA_0.3_Sn_0.5_Pb_0.5_I_3_) perovskite, and a representative two-terminal (2T) all-perovskite tandem solar cells. For the single-junction devices, the highest power conversion efficiency (PCE) achieved was 22.37% for the narrow bandgap devices, while that for the wide bandgap devices reached 18.82%. The best-performing 2T all-perovskite tandem device exhibited the highest PCE of 27.28%. The detailed photovoltaic parameters are summarized in Supplementary Table 1 and Supplementary Fig. 1. Figure 1b shows the stability test of these three devices, where a mercury lamp was employed to accelerate the aging process. After 60 min of irradiation, the PCE of the 2T all-perovskite tandem solar cells degrade to 33.37% of its original value, while the wide bandgap perovskite single-junction solar cells only degrade to 68.56% of their original value. Surprisingly, the narrow bandgap perovskite single-junction solar cells’ PCE degraded to nearly 0%. Therefore, the instability of the narrow bandgap perovskites is the main reason for the reduced efficiency of the 2T all-perovskite tandem solar cells. It is important to note that the stability of all-perovskite tandems is generally superior to that of their single-junction mixed tin-lead perovskite counterparts. This is primarily because the light irradiation on the bottom tin-lead perovskite subcells in tandem configurations is considerably weaker, as it is filtered by the top wide bandgap subcells.

Given that the stability of all-perovskite tandem solar cells is limited by narrow-bandgap subcells, we focus on the instability mechanisms of narrow-bandgap perovskites. Figure 1c, d illustrate the external quantum efficiency (EQE) and absorption spectra of a single-junction narrow-bandgap perovskite solar cell and a narrow-bandgap perovskite thin film under irradiation, respectively. Notably, the edge of the EQE spectra approximately shifts from 1020 nm to 790 nm after 60 min of irradiation (Supplementary Fig. 1b). The absorption spectra present a similar trend as the EQE spectra (Supplementary Fig. 1c, d). It is noted that the edge at 1020 nm is related to the band gap of FA_0.7_MA_0.3_Sn_0.5_Pb_0.5_I_3_ perovskites. However, the shift to 790 nm indicates that the perovskite compounds underwent changes during the aging process.

### Phase reconstruction processes in perovskite thin films with various components

**Fig. 2:**
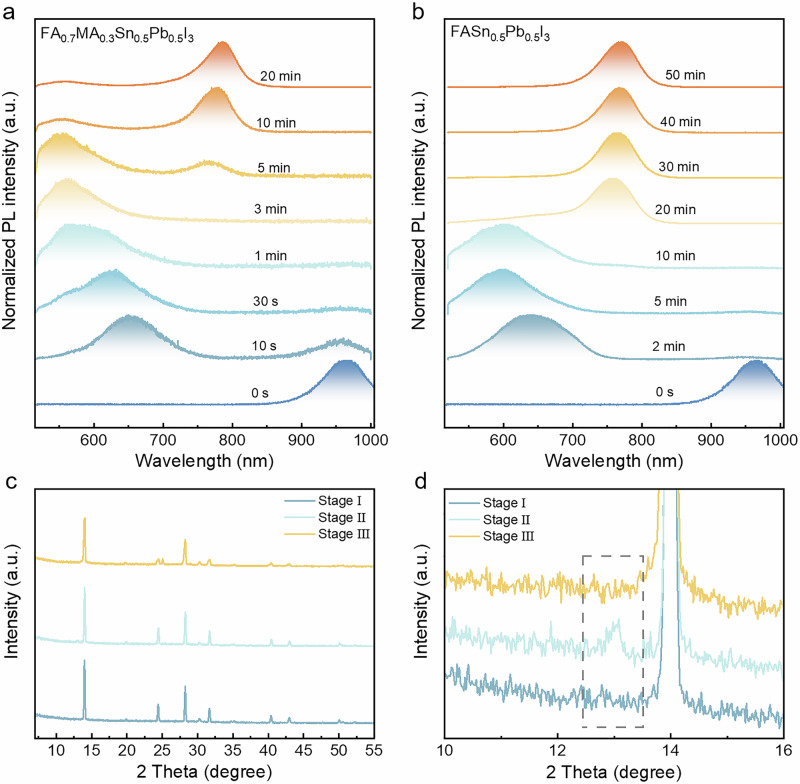
In-situ observation of the reconstruction of mixed tin-lead perovskite films. **a** Evolution of normalized PL spectrum results of FA_0.7_MA_0.3_Sn_0.5_Pb_0.5_I_3_ thin films under irradiation/air exposure (Mercury lamp, 13 W cm^−^^2^). **b** Evolution of normalized PL spectrum results of FASn_0.5_Pb_0.5_I_3_ thin films under irradiation/air exposure (Mercury lamp, 13 W cm^−2^). **c** XRD patterns of FA_0.7_MA_0.3_Sn_0.5_Pb_0.5_I_3_ thin films in different reconstruction stages. **d** Closed-up of XRD patterns of FA_0.7_MA_0.3_Sn_0.5_Pb_0.5_I_3_ thin films in different reconstruction stages.

To explore the physics behind this edge shift under irradiation, an in-situ technique is required to monitor this transition process. Previously, photoluminescence (PL) imaging has been demonstrated as an effective method for studying the phase separation and reconstruction processes in mixed bromide and iodide perovskites^32,33^. In this study, PL spectra are recorded to in-situ track the entire process of tin-lead perovskites under irradiation. A schematic diagram of the setup is provided in Supplementary Fig. 2. We selected two typical tin-lead perovskite compounds for investigation: FA_0.7_MA_0.3_Sn_0.5_Pb_0.5_I_3_, used in high-performance devices, and the more stable, MA-free FASn_0.5_Pb_0.5_I_3_. Figure 2a, b showcase the evolution of the PL spectra for FA_0.7_MA_0.3_Sn_0.5_Pb_0.5_I_3_ and FASn_0.5_Pb_0.5_I_3_ thin films, respectively, under irradiation. Given that the maximum intensity of the solar spectrum occurs at 460–490 nm, a near-realistic condition is selected to simulate the impact of blue light on the tin-lead perovskites (Supplementary Fig. 3a, b). The spectrum of the light source and the temperature conditions are depicted in Supplementary Fig. 3c. Notably, FA_0.7_MA_0.3_Sn_0.5_Pb_0.5_I_3_ thin films exhibit similar phenomena under AM 1.5 G spectra with a standard solar simulator as they do under blue light emitted by a mercury lamp (Supplementary Fig. 3d). Therefore, the blue light irradiation conditions are chosen to accelerate the reconstruction processes. Unless specified otherwise, all degradation experiments reported herein were conducted by exposing the samples to ambient air (relative humidity, RH = 38.0 ± 7.5%; temperature, *T* = 24.5 ± 0.9 °C; oxygen content, 20.9%), which is consistent with normal working conditions.

The entire process of the PL emission evolution in FA_0.7_MA_0.3_Sn_0.5_Pb_0.5_I_3_ thin films can be categorized into three distinct stages (Fig. 2a): Stage I: Initially, FA_0.7_MA_0.3_Sn_0.5_Pb_0.5_I_3_ thin films exhibit only a PL emission peak at 960 nm, corresponding to the original PL peak of the material. Stage II: Upon continued irradiation, the PL peak commences a blue shift. Notably, a PL peak appears at 650 nm after just 10 s of irradiation. The PL peak continues to evolve and by approximately 3 min of irradiation, it stabilizes at around 590 nm, signaling a significant structural transformation within the material. Stage III: By the 5-min mark, another PL emission peak is observed at 790 nm. As irradiation proceeds, the PL intensity at 590 nm diminishes, while the emission spectrum is increasingly dominated by the 790 nm peak, characteristic of the black phase of FAPbI_3_. The FASn_0.5_Pb_0.5_I_3_ thin films present a similar transformation process as the FA_0.7_MA_0.3_Sn_0.5_Pb_0.5_I_3_ thin films (Fig. 2b). Notably, the entire reconstruction process is irreversible and cannot be attributed to moisture and thermal effects (Supplementary Figs. 4–6)^34^. The thin films have been annealed under various temperatures and times and the PL spectra show a totally different trend. After 10 min of annealing, a PL peak appears at around 600 nm, and no additional PL emission emerges at 790 nm during continued annealing (Supplementary Fig. 5). The PL spectra at different humidity levels are also generally consistent, suggesting that moisture does not affect the reconstruction process (Supplementary Fig. 6). Therefore, both moisture and thermal effects can be excluded from the observed phenomena. Factors affecting the overall reconstruction process include oxygen content, light wavelength, and power (Supplementary Figs. 7–9). Thin films irradiated under varying oxygen levels exhibit differences in PL spectra timescales. Notably, the degradation rate accelerates at high oxygen content and decelerated under low oxygen conditions (Supplementary Figs. 7 and 8). Additionally, PL spectra recorded at different light wavelengths and intensities reveal diverse timescales, indicating that both wavelength and light intensity are significant factors in the reconstruction process (Supplementary Fig. 9). It was expected that tin-lead mixed perovskites would easily degrade into other compounds under continuous irradiation. However, surprisingly, they transition to the black phase of FAPbI_3_, passing through an intermediate state with a PL emission at 590 nm^35^. The reconstruction process exhibited by tin-lead perovskites under continuous blue light irradiation showcases significant shifts in PL behavior, reflecting structural changes at the molecular level. Each stage represents a distinct phase transition triggered by the irradiation, highlighting the dynamic nature of these materials under external energy influences.

To further investigate the structure of this intermediate state, X-ray diffraction (XRD) measurements were conducted. Since the reconstruction processes are irreversible, the structures at each stage could be independently studied. Figure 2c presents the XRD results of the FA_0.7_MA_0.3_Sn_0.5_Pb_0.5_I_3_ perovskite thin films at different stages. At Stage I, the XRD patterns confirm a pure phase of FA_0.7_MA_0.3_Sn_0.5_Pb_0.5_I_3_. At Stage II, an additional XRD peak at 13.0° is observed, associated with the 4H phase of FAPbI_3_. This aligns with the PL measurements, where the 4H phase FAPbI_3_ is identified by a PL emission at 590 nm^36,37^. This process is known as superoxidation, which will be discussed later. At Stage III, the peak at 13.0° disappears, indicating the absence of the 4H phase FAPbI_3_ (Fig. 2d)^38^. The XRD patterns at this stage show an additional peak at 25.1°, which is indicative of SnO_2_, suggesting an oxidation process of Sn (Supplementary Fig. 10). These stages highlight a complex and dynamic transformation process in the perovskite structure under irradiation, shedding light on both the material’s instability and its potential pathways to stabilization in a distinct phase.

For the dynamic transformation process of perovskites under irradiation, we have investigated various components of tin-based and lead-based perovskite films. A similar phenomenon is observed in FA_0.5_MA_0.5_Sn_0.5_Pb_0.5_I_3_ thin films, where an increase in MA content leads to a PL emission peak at 745 nm, as shown in Supplementary Fig. 11. This peak is due to Pb enrichment, resulting in the concurrent formation of MAPbI_3_. The final state of the thin film exhibits a PL emission peak at 790 nm after 20 min of irradiation, suggesting that the phase reconstruction process is independent of A-site cations. Furthermore, the lead and tin-based perovskite films do not exhibit the same reconstruction process as tin-lead thin films when subjected to irradiation (Supplementary Figs. 12–14). The dynamics of irradiation in perovskites thin films prepared with different tin-lead ratios is also intriguing (Supplementary Fig. 15). With sufficiently long irradiation, the overall trend favors the appearance of PL peaks near 790 nm presenting similar reconstruction process (Supplementary Fig. 16). When the incident light enters through the back side of the perovskite thin films, the reconstruction process still begins on the side in contact with the air (Supplementary Fig. 18). These findings suggest that the device failure is interfacial in nature. To confirm this, surface modification of perovskite thin films—through encapsulation or oxygen isolation—has been shown to mitigate irradiation-induced degradation to varying extents (Supplementary Fig. 17). This underscores the importance of examining surface properties at different stages of degradation.

### Surface chemical states for phase reconstruction

**Fig. 3:**
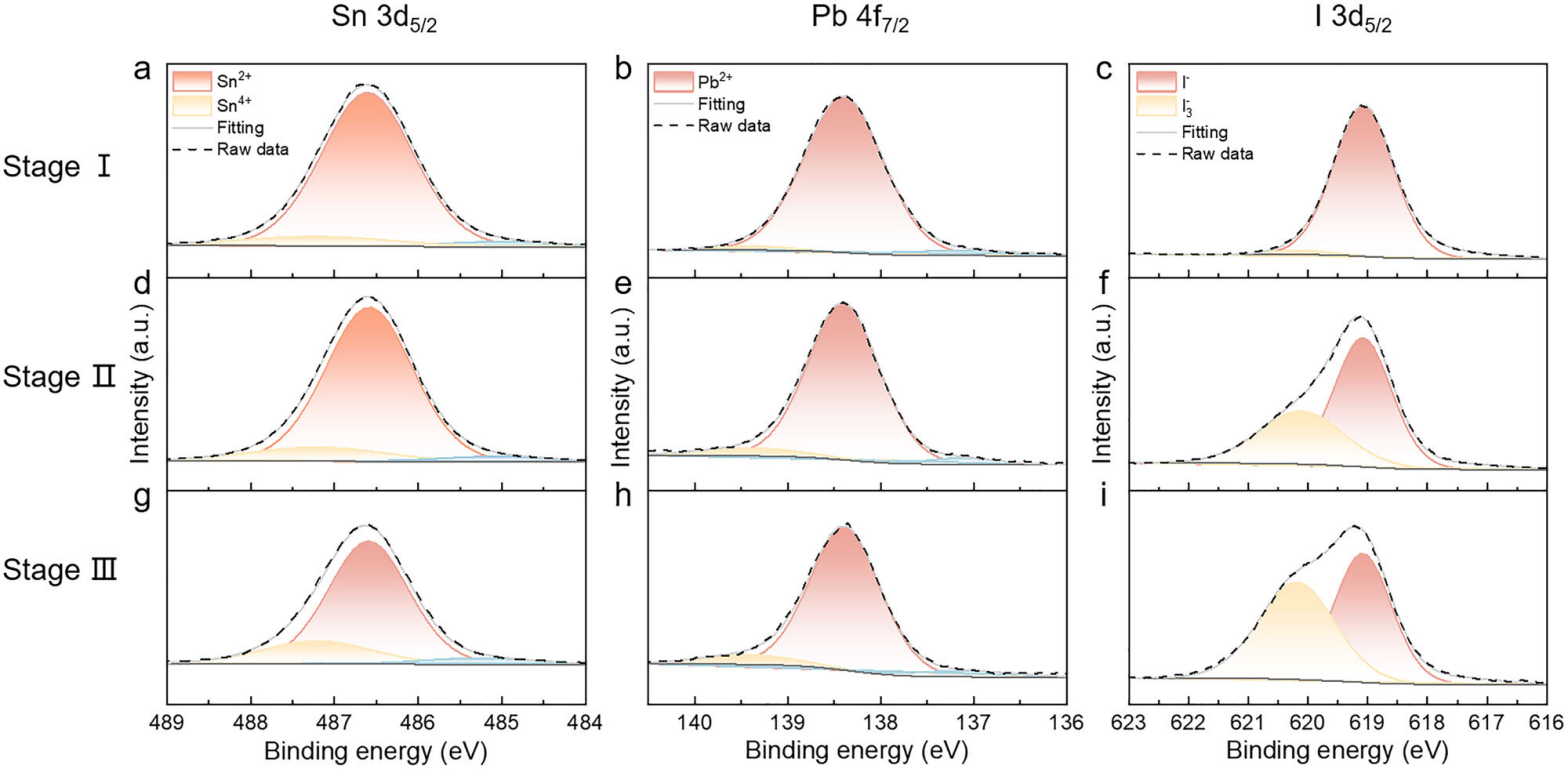
The surface element properties of mixed tin-lead perovskite films during phase reconstruction. **a**–**c** XPS spectra of mixed FA_0.7_MA_0.3_Sn_0.5_Pb_0.5_I_3_ perovskite films in stage I: (**a**) Sn 3d_5/2_; (**b**) Pb 4f_7/2_; (**c**) I 3d_5/2_. **d**–**f** XPS spectra of mixed FA_0.7_MA_0.3_Sn_0.5_Pb_0.5_I_3_ perovskite films in stage II: (**d**) Sn 3d_5/2_; (**e**) Pb 4f_7/2_; (**f**) I 3d_5/2_. **g**–**i** XPS spectra of mixed FA_0.7_MA_0.3_Sn_0.5_Pb_0.5_I_3_ perovskite films in stage III: (**g**) Sn 3d_5/2_; (**h**) Pb 4f_7/2_; (**i**) I 3d_5/2_.

The surface properties of the irradiated films are analyzed using X-ray photoelectron spectroscopy (XPS). Figure 3 displays the XPS results for the initial tin-lead perovskite films (Stage I), aged thin films under blue light irradiation for 10 min (Stage II), and aged thin films under blue light irradiation for 60 min (Stage III). Stage I: The primary signal is the lead-iodide peak with a binding energy of 138.4 eV, consistent with the lead-iodide environment within the perovskites. A smaller peak at 137.2 eV correlates with the Sn 4 s core level. Figure 3a, c show the Sn 3d_5/2_ peak at 486.6 eV and the I 3d_5/2_ peak at 619.1 eV, respectively. Stage II: As shown in Fig. 3d–f, the Pb 4f_7/2_ peak remains almost unchanged^39–41^. However, there is a slight increase in peak intensity at a higher binding energy (487.4 eV) for the Sn 3d_5/2_ peak, indicative of the oxidation of Sn^2+^ to Sn^4+^. Additionally, it is noted a high-energy hump on the I 3d_5/2_ at 620.1 eV, associated with covalent bonding. The superoxidation process likely causes molecular iodine to escape to the surface due to its volatility, further binding with iodide ions to form triiodide ions. Stage III: Fig. 3g, h indicate further slow oxidation of Sn^2+^ to Sn^4+^. Additionally, the peak area of triiodide ions dramatically increases to approximately 42.27% of the 3d_5/2_ peak area, signaling further formation of triiodide ions (Supplementary Table 2 and Supplementary Fig. 19). It is noteworthy that a distinct inconsistency exists between the changes in film surface elements during the reconstruction process and those following thermally induced degradation (under comparable temperature and duration conditions). The surface of thermally degraded thin films show significantly fewer triiodide ions and a higher Sn^4+^ concentration compared to irradiated films (Supplementary Fig. 20). The XPS profiles of oxygen across different stages further reveal that irradiation-induced degradation leads to more pronounced oxidation of the film than heating (Supplementary Fig. 21a, b). This indicates an accelerated generation of iodine vacancies during irradiation-induced superoxide degradation. Additionally, the rapid increase in the atomic ratio of lead ions to iodide ions during irradiation-induced phase reconstruction corroborates the formation of Pb-rich regions (Supplementary Fig. 21c). This XPS analysis underscores the complex interplay of oxidation processes and ionic transformations in perovskites under irradiation, significantly affecting the stability and electronic structure of the materials.

### Surface ion distribution of irradiated thin films

**Fig. 4:**
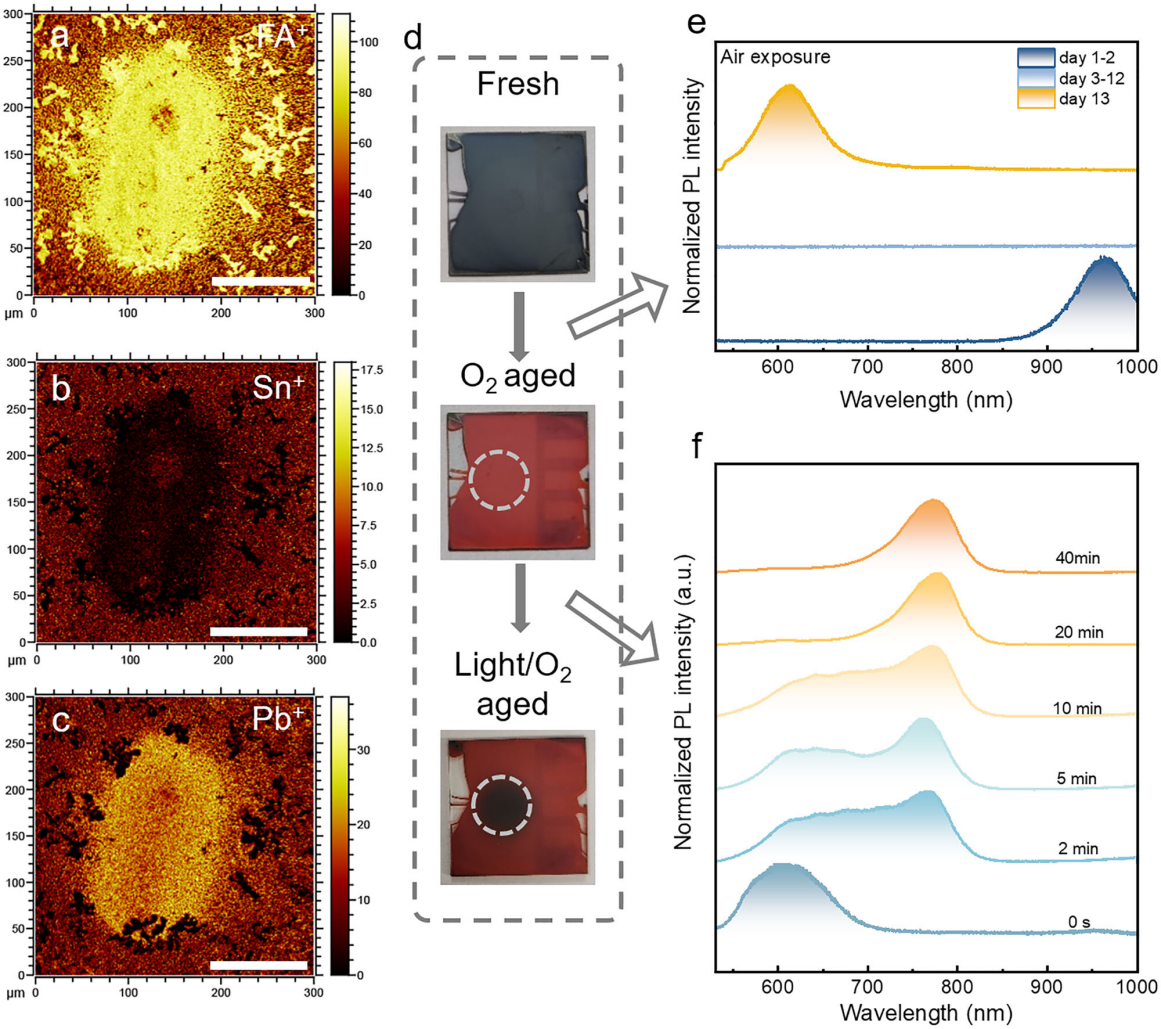
The surfaced ions distribution of mixed tin-lead perovskite thin films during phase reconstruction and the phase reconstruction processes in air-aged tin-lead perovskite thin films. **a**–**c** TOF-SIMS mapping signals of (**a**) FA^+^, (**b**) Pb^+^, (**c**) Sn^+^ on the mixed FASn_0.5_Pb_0.5_I_3_ perovskite films in stage III. The scale bar is 100 µm. **d** Digital images of the fresh FA_0.7_MA_0.3_Sn_0.5_Pb_0.5_I_3_ perovskite films, the films with air exposure, and the films with continuous blue-light irradiation after air exposure. **e** Evolution of normalized PL spectrum results of FA_0.7_MA_0.3_Sn_0.5_Pb_0.5_I_3_ perovskite films with air exposure. **f** Evolution of normalized PL spectrum results of FA_0.7_MA_0.3_Sn_0.5_Pb_0.5_I_3_ perovskite films with air exposure under continuous blue-light irradiation (Mercury lamp, 13 W cm^−2^).

To further confirm the composition at Stage III, time-of-flight secondary ion mass spectrometry (TOF-SIMS) was employed. The TOF-SIMS results for the region at Stage III, as shown in Fig. 4a–c, reveal an interesting pattern. After irradiation, there is a simultaneous enrichment of FA^+^ and Pb^+^ signals, alongside a diminished signal of Sn^+^. The signal of the SnI_3_^−^ is barely detectable, whereas the signal distribution of the PbI_3_^−^ is consistent with that of the Pb^+^ (Supplementary Fig. 22). Additionally, Sn^+^ ions are found to be less distributed in areas where Pb^+^ concentrations are higher, suggesting that the lattice becomes Pb-rich during the reconstruction process. Moreover, a comparison of TOF-SIMS results across Stages I, II, and III reveals enhanced signals of FA^+^, Pb^+^, and I^−^ within the same region, while the Sn^+^ signal in this area is significantly reduced (Supplementary Fig. 23). This observation supports the hypothesis that the formation of Pb-rich regions is directly associated with the oxidation of Sn^2+^ and potentially leads to the migration of Sn^2+^ out of the perovskite lattice. This is also evident in FA_0.7_MA_0.3_Sn_0.5_Pb_0.5_I_3_ thin films, further supporting the universality of this process in tin-lead perovskite (Supplementary Fig. 24). Despite the stronger binding energy of Sn-I compared to Pb-I, the instability of Sn^2+^ in air facilitates the formation of these Pb-rich regions in the thin films after irradiation. The TOF-SIMS results not only support the observed changes in the films’ composition but also help confirm the final stage reconstruction of FAPbI_3_. This final transformation indicates a significant alteration in the material’s structure and composition, driven by the irradiation process and the inherent instability of Sn^2+^ in the perovskite matrix.

To confirm the universality of the reconstruction process, tin-lead perovskite thin films were first aged in the air and subsequently subjected to irradiation. Figure 4d presents digital images that capture the entire process. Intriguingly, the black perovskites turn red upon air exposure and revert to black under irradiation. Comparing fresh and aged tin-lead perovskite thin films, it is evident that the crystal quality of the aged films has dramatically decreased, with no characteristic XRD peaks of PbI_2_, SnI_4_, or SnO_2_ observed. This lack of peaks can be attributed to the amorphous and disordered nature of structural domains in the XRD pattern (Supplementary Fig. 25). Figure 4e shows the evolution of the PL spectra under irradiation. Initially, there are two distinct peaks positioned at 620 nm and 790 nm, respectively. The 620 nm peak is associated with FA_2_SnI_6_, while the 790 nm peak corresponds to the black phase of FAPbI_3_^42,43^. After 40 min of irradiation, the PL peak at 620 nm disappears, and the PL spectrum is dominated by the 790 nm peak, indicative of the formation of the black phase of FAPbI_3_. This reproducible phenomenon underscores the universal and irreversible behavior of tin-lead perovskites under irradiation (Supplementary Fig. 26). To further understand the role of irradiation in this phase reconstruction process, the aged tin-lead perovskites were annealed at various temperatures. Notably, none of the perovskites show the black phase of FAPbI_3_ under these conditions, suggesting that the observed phase reconstruction process cannot be attributed to thermal effects (Supplementary Fig. 27). Therefore, irradiation plays a crucial role in the phase reconstruction of tin-lead perovskites, emphasizing its significance in influencing the material’s properties and behavior.

### The nexus between irradiation and phase reconstruction in tin-lead perovskite

**Fig. 5:**
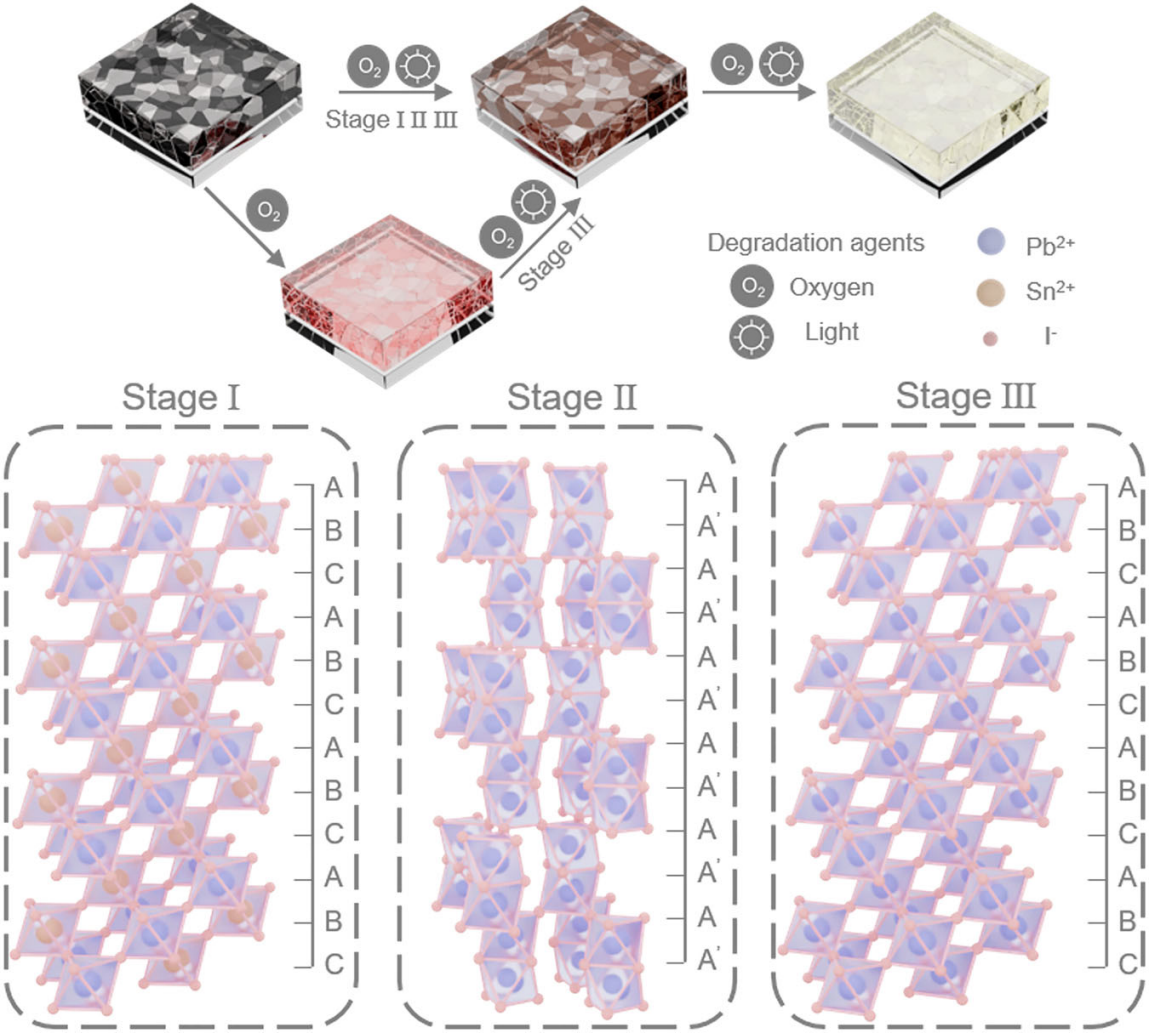
Mechanism of the reconstruction of mixed tin-lead perovskites. Scheme for the evolution from tin-lead perovskites to a reconstruction of lead perovskites under light.

Extending the previous practice on superoxide-induced degradation in FASnI_3_ perovskite, Fig. 5 illustrates the phase reconstruction mechanism induced by superoxides in tin-lead perovskite thin films. The tin-lead perovskite thin films at Stage I are prepared with the stoichiometric of FA_0.7_MA_0.3_Sn_0.5_Pb_0.5_I_3_. Under continuous blue light irradiation, the oxidation of Sn ions within the lattice induces the formation of FA_2_SnI_6_ in the film^44^. With extended irradiation, FA_2_SnI_6_ undergoes further oxidation, leading to the formation of the yellow phase of FAPbI_3_ (Stage II). After nearly an hour of irradiation, the films finally transform into the black phase of FAPbI_3_ (Stage III). These processes are driven by incident light with energies exceeding the band gap, which activates absorbed oxygen through charged electrons in the conduction band^45^. This activation deprotonates FA^+^ and accelerates the oxygen-induced oxidation of Sn^2+^. To facilitate understanding, we use FASn_0.5_Pb_0.5_I_3_ as the main component of the reaction formula, described by the following equation:

1 $${{{{\rm{FASn}}}}}_{0.5}{{{{\rm{Pb}}}}}_{0.5}{{{{\rm{I}}}}}_{3}{-\!\!\!-\!\!\!-\!\!\!-\!\!\!-\!\!\!-\!\!\!-\!\!\!-\!\!\!-\!\!\!-\!\!\!-\!\!\!-\!\!\!-\!\!\! \longrightarrow }^{{\mbox{light no moisture}}}{{{{\rm{FASn}}}}}_{0.5}{{{{\rm{Pb}}}}}_{0.5}{{{{\rm{I}}}}}_{3}^{*}$$

2 $${{{{\rm{O}}}}}_{2}{-\!\!\!-\!\!\!-\!\!\!-\!\!\!-\!\!\!-\!\!\! \longrightarrow }^{{{{{\rm{FASn}}}}}_{0.5}{{{{\rm{Pb}}}}}_{0.5}{{{{\rm{I}}}}}_{3}^{*}}{{{{\rm{O}}}}}_{2}^{-}$$

Incident light can accelerate the oxygen-induced oxidation of Sn^2+^ by activating the absorbed oxygen with energized electrons in the conduction band^45^. The wavelength of light with energy higher than the band gap does not significantly affect phase reconstruction (Supplementary Fig. 9a, c). If the irradiation density is halved, the time required for the reconstruction process doubles. One possible explanation is that the reconstruction process depends on the irradiation flux (Supplementary Fig. 9d). This result robustly supports our proposed mechanism of superoxide-induced phase reconstruction. Elevated irradiation densities produce a greater number of electrons and holes, which in turn enhances charge-oxygen interactions. These intensified interactions expedite the phase reconstruction process. Consequently, the reconstruction rate is dependent on the quantity of excited electrons and holes, rendering the entire process proportional to the irradiation density. The tin-lead perovskite undergoes a series of phase transitions during superoxide degradation, we predict the following potential degradation pathways induced by superoxide in FASn_0.5_Pb_0.5_I_3_ films.

3 $$4{{{\rm{FASn}}}}_{0.5}{{{{\rm{Pb}}}}}_{0.5}{{{{\rm{I}}}}}_{2}^{*}\to {{{{\rm{FA}}}}}_{2}{{{\rm{Sn}}}}{{{{\rm{I}}}}}_{6}+{{{\rm{Sn}}}}{{{{\rm{O}}}}}_{2}+2{{{\rm{FAPb}}}}{{{{\rm{I}}}}}_{3}$$

4 $$2{{{{\rm{FA}}}}}_{2}{{{\rm{Sn}}}}{{{{\rm{I}}}}}_{6}+3{{{{\rm{O}}}}}_{2}\to 4{{{{\rm{CH}}}}}_{4}{{{{\rm{N}}}}}_{2}+2{{{{\rm{H}}}}}_{2}{{{\rm{O}}}}+2{{{\rm{Sn}}}}{{{{\rm{O}}}}}_{2}+6{{{{\rm{I}}}}}_{2}$$

5 $${{{{\rm{I}}}}}_{2}+{{{{\rm{I}}}}}^{-}\to {{{{\rm{I}}}}}_{3}^{-}$$

Stage I to Stage II: Based on experimental results, it is inferred that reaction (3) and (4) proceed at a rapid rate, with FA_2_SnI_6_ existing only transiently during the initial stages of the reaction. Thus, the PL spectrum continues to evolve after a few minutes of irradiation, it stabilizes at around 590 nm which is at Stage II. It is noteworthy that when Sn^2+^ is oxidized to Sn^4+^, it readily transforms into the FA_2_SnI_6_ structure. Unlike the common perovskite structure, the [SnI_6_]^4^^−^ units in the FA_2_SnI_6_ structure do not connect through the iodide ions, but form a face-centered cubic arrangement with the A-site ions occupying the interstitials. The further progress of the reaction is accompanied by an increase in triiodide ions (reaction (5)) as well as Sn^4+^. Under irradiation, iodide ions are easily oxidized to form I_2_, which then combines with iodide ions to form triiodide ions^46^. This observation is consistent with the XPS results. The transformation of Sn ions into the disordered free state does not result in lattice collapse and facilitates the formation of the yellow phase of FAPbI_3_.

Stage II to Stage III: Due to the significantly slower mobility of Sn ions (with a typical timescale of hours) compared to halide ions (timescale of minutes), and in conjunction with the near-hourly reconstruction observed in Stage III, we conclude that the black phase of FAPbI_3_ arises from the migration and accumulation of Sn ions^47–49^. The degradation of FA_2_SnI_6_ or the formation of Sn^4+^ under prolonged irradiation results in numerous Sn vacancies within the thin films. The migration and clustering of these ions contribute to the development of Pb-rich regions, while the original tin-lead lattice remains structurally intact. Consequently, Pb-rich regions emerge in the black phase FAPbI_3_.

To summarize, we have demonstrated that tin-lead perovskites undergo superoxide-induced degradation under irradiation in air. During this degradation process, even when Sn is oxidized to an amorphous form, it does not cause lattice collapse. This property is crucial to the dynamic phase reconstruction that occurs in thin films under irradiation. The mechanistic insights gained from this study reveal that the superoxidation process of tin-lead perovskite thin films is not only an isolated Sn^2+^ oxidation process but is also accompanied by the oxidation of iodide to generate triiodide ions. Despite the relatively low activation energy for halogen ion migration and their susceptibility to light-induced ion movement, prolonged irradiation eventually causes cation migration, triggering a phase transition in the tin-lead perovskite. We therefore recommend further investigation into fabrication methods for mixed tin-lead perovskites. This includes exploring an iodide-rich environment to prevent iodide vacancies and optimizing the concentration of tin additives to carefully balance the trade-off between tin-vacancy and tin-interstitial formation. Additionally, greater emphasis should be placed on eliminating tin inhomogeneities. These inhomogeneities may result in tin-lead perovskites with nominally optimized tin stoichiometry but exhibit a combination of locally tin-rich environments, where tin interstitials are dominant, and locally tin-poor environments, where tin vacancies are predominant. Our study underscores the pressing need to develop alternative passivation methods specifically tailored for mixed tin-lead perovskites.

## Discussion

In conclusion, we have identified and monitored the phase reconstruction process of tin-lead perovskites under irradiation. This process is driven by superoxide generated from the reaction between O_2_ molecules and photoexcited electrons, leading to the formation of vacancies through the oxidation of Sn^2+^ on the surface of the tin-lead perovskite thin films. When the formation of Sn^2+^ vacancies is accompanied by the generation of triiodide ions, Pb-rich regions are simultaneously created at the vacancies. These Pb-rich regions facilitate the random generation of black phase perovskite from yellow phase perovskite under irradiation, transitioning into a different phase of FAPbI_3_. Considering the fundamental nature of the revealed redox chemistry, we anticipate that these processes cannot be fully prevented but could be functionally mitigated. This work provides significant insights into the phase reconstruction of perovskites and sheds light on understanding the property evolution under irradiation.

## Methods

### Materials

All chemicals without further purification were used for preparing perovskite precursors. FAI (99.9%), methylammonium iodide (MAI, 99.9%), SnI_2_ (99.99%) and ITO (12 Ω sq^−1^) were purchased from Advanced Election Technology Co., Ltd. Tin fluoride (SnF_2_, 98%), N, N-dimethylformamide (DMF, 99.8%), dimethyl sulphoxide (DMSO, 99.8%), chlorobenzene (CB, 99.8%), and lead thiocyanate (Pb(SCN)_2_,99.5%) were bought from Sigma Aldrich (Shanghai) Trading Co., Ltd. C60 (99.9%) and Copper (Cu) were purchased from Xi’an Yuri Solar Co., Ltd. PbI_2_ (99.99%) was bought from Tokyo Chemical Industry (Shanghai) development Co., Ltd. PEDOT:PSS was purchased from Heraeous Germany Co., Ltd. BCP (99.5%) was purchased from Jilin OLED Material Tech Co., Ltd.

### Precursor solutions preparation

WBG FA_0.8_Cs_0.2_PbI_1.8_Br_1.2_ perovskite precursors with a concentration of 1.1 M were synthesized by dissolving FAI, CsI, PbI_2_, PbBr_2_, and Pb(SCN)_2_ in a mixed solvent of DMF and DMSO with a 3:1 volume ratio. The molar ratio of FAI to CsI was 4:1, and the molar ratio of PbI_2_ to PbBr_2_ to Pb(SCN)_2_ was 2:3:0.02. The resulting precursors were stirred at 70 °C for 3 h prior to usage.

NBG FA_0.7_MA_0.3_Pn_0.5_Sn_0.5_I_3_ perovskite precursor solutions, with a concentration of 1.8 M, were synthesized by dissolving FAI, MAI, PbI_2_, SnI_2_, SnF_2_, and Pb(SCN)_2_ in a mixed solvent of DMF and DMSO with a 3:1 volume ratio. The molar ratio of FAI to MAI was 7:3, and the molar ratio of PbI_2_ to SnI_2_ to SnF_2_ to Pb(SCN)_2_ was 1:1:0.1:0.02. The resulting precursors were stirred at 25 °C for 3 h before application.

### Fabrication of single-junction NBG PSCs

The substrates were coated with PEDOT:PSS solutions via spin-coating at 5000 rpm for 30 s and then annealed at 140 °C for 30 min. Following annealing, the substrates were promptly moved into an N_2_-filled glovebox. Perovskite precursors were deposited onto the substrates directly using a one-step spin-coating method at 1000 rpm for 10 s followed by 4000 rpm for 40 s. At the twentieth second before the spin-coating process concluded, 400 μl of CB was applied onto the films. The substrates with perovskite films underwent annealing at 100 °C for 10 min. Subsequently, posttreatment solutions were spin-coated onto the perovskite films at 4000 rpm for 30 s, and the films were annealed at 100 °C for 7 min. Finally, layers of C_60_ (20 nm), BCP (7 nm), and Cu (80 nm) were sequentially deposited onto the perovskite films using a thermal evaporator.

### Fabrication of single-junction WBG PSCs

The substrates were promptly transferred into a glovebox filled with nitrogen. A 2PACz solution (0.3 mg ml^−1^) was spin-coated onto the ITO substrates at 3000 rpm for 30 s and annealed at 100 °C for 10 min. Subsequently, perovskite precursor solutions were spin-coated at 4000 rpm for 30 s. At the tenth second before the spin-coating process concluded, 500 μl of diethyl ether was added to the center of the films. The perovskite films were annealed at 70 °C for 2 min and then at 100 °C for 8 min. Propane-1,3-diammonium iodide solutions were then spin-coated onto the perovskite films at 4000 rpm for 30 s and annealed at 100 °C for 5 min. Following post-treatment, the films were transferred to a thermal evaporator chamber where 20 nm of C_60_ was thermally deposited. Subsequently, the substrates were introduced into an ALD system to deposit a 20-nm-thick (130 cycles) SnO_2_ layer at 90 °C. Finally, a 80 nm layer of Cu was thermally evaporated onto the substrates.

### Fabrication of 2T all-perovskite tandem solar cells

WBG subcells were initially assembled using identical procedures to those used for single-junction WBG PSCs fabrication, up to the ALD-SnO_2_ layer step. Next, the substrates were shifted to a thermal evaporator for the deposition of a 1-nm-thick Au interlayer. Subsequently, NBG subcells were deposited onto the substrates, utilizing the same procedures as for single-junction NBG PSCs fabrication.

### Characterizations

PL measurements: Typically, PL spectra were acquired using a Halogen lamp (U-LH100-3, Olympus) for excitation, passed through a ≈460–490 nm bandpass filter. The excitation power under the 100× objective was approximately ≈1.04 W cm^−2^, as measured by an optical power meter. For capturing PL imaging results, a mercury lamp (U-LH100HG, Olympus) served as excitation, filtered through an ≈460–490 nm bandpass filter. The excitation power under the 100× objective was estimated at ≈13.0 W cm^–2^. The signal was filtered through a 520 nm long-pass filter before being collected by either a CCD camera (Tucsen, TCH-1.4CICE) or a spectrometer (Renishaw inVia). XRD spectra were obtained using an X’pert PRO X-ray diffractometer with Cu K*α* radiation at 40 mA and 40 kV, covering a range from 5° to 55° at a scanning speed of 5° min^−1^. XPS spectra were acquired using a Kratos AXIS Supra X-ray photoelectron spectrometer. Samples were prepared as 5 × 5 mm^2^ pieces and affixed to the sample stage using tape. The voltage during full spectrum collection was set at 15 kV with a current of 5 mA, and for each element, the voltage was 15 kV and the current was 10 mA. ToF-SIMS measurements were conducted using a focused ion beam ToF-SIMS spectrometer (GAIA3 GMU Model 2016, Czech). Absorption spectra were measured using a SHIMADZU mini 1280 UV–vis spectrophotometer.

### Device performance measurements

*J–V* measurements were conducted using a Keithley 2400 source meter coupled with a solar simulator (SS-X50, Enlitech), employing AM 1.5 G spectra. Spectra intensity was calibrated utilizing a certified WPVS standard solar reference cell (SRC-2020, Enlitech), traceable to the National Renewable Energy Laboratory, at an irradiance of 100 mW cm^−2^. Bias voltages for single-junction cell *J–V* measurements ranged from −0.1 V to 1 V and from 1 V to −0.1 V, with a scanning rate of 0.2 V s^−1^, a voltage step of 50 mV, and a delay time of 25 ms. For tandem cell *J–V* measurements, bias voltages ranged from −0.1 V to 2.2 V and from 2.2 V to −0.1 V, following the same scanning parameters. The device area was 0.0948 cm^2^, with a metal aperture defining an active area of 0.070225 cm^2^. EQE spectra were obtained using a quantum efficiency/incident photon to current conversion efficiency system from Enli Technology Co., Ltd. For tandem solar cell EQE measurements, light-emitting diodes emitting at wavelengths of 450 nm and 850 nm were employed as bias lights for NBG and WBG subcells, respectively.

### Reporting summary

Further information on research design is available in the Nature Portfolio Reporting Summary linked to this article.

## Supplementary information

Supplementary Information

Reporting Summary

Peer Review file

## Data Availability

The data that support the plots within this paper and other finding of this study are available from the corresponding author at request.
