# Supplementary material for: Unveiling the nexus between irradiation and phase reconstruction in tin-lead perovskite solar cells
Source: Nat Commun. 2025 Jan 8;16:506. doi: 10.1038/s41467-025-55814-0 (PMC11711683; doi:10.1038/s41467-025-55814-0)
Supplement: Supplementary file 1 — Supplementary Information [file 41467_2025_55814_MOESM1_ESM.pdf]

Supplementary Information for Unveiling the nexus between irradiation and phase reconstruction in tin-lead perovskite solar cells

Wenbo Li,[1#] Zhe Li,[1#] Shun Zhou,[1#] Yanzhuo Gou,[2] Guang Li,[1] Jinghao Li,[3] Cheng Wang,[1] Yan Zeng,[1] Jiakai Yan,[1] Yan Li,[1] Wei Dai,[1] Yaoguang Rong,[3] Weijun Ke,[1*] Ti Wang,[1*] and Hongxing Xu[1,4,5,6*]

[1] School of Physics and Technology, and Key Laboratory of Artificial Micro- and Nano-structures of Ministry of Education, Wuhan University, Wuhan 430072, China

[2] School of Materials Science and Engineering, Hubei University, Wuhan 430062, China

[3] State Key Laboratory of Advanced Technology for Materials Synthesis and Processing, Wuhan 430070, China

[4] School of Microelectronics, Wuhan University, Wuhan 430072, China

[5] Wuhan Institute of Quantum Technology, Wuhan 430206, China

[6] Henan Academy of Sciences, Zhengzhou 450046, China

[#] These authors contributed equally to this work

[*] Correspondence: weijun.ke@whu.edu.cn

[*] Correspondence: wangti@whu.edu.cn

[*] Correspondence: hxxu@whu.edu.cn

Contents
Supplementary Figures

Fig. S1. Performance and optical property of different perovskite soalr cells.

Fig. S2. Schematic of the PL microscopy experiment setup.

Fig. S3. Experimental conditions and phenomena under irradiation.

Fig. S4. Probing the reversibility of $FA_{0.7}MA_{0.3}Sn_{0.5}Pb_{0.5}I_3$ perovskite phase reconstruction.

Fig. S5. Effect of heating on $FA_{0.7}MA_{0.3}Sn_{0.5}Pb_{0.5}I_3$ perovskite thin films.

Fig. S6. Effect of moisture on $FA_{0.7}MA_{0.3}Sn_{0.5}Pb_{0.5}I_3$ perovskite thin films under irradiation.

Fig. S7. Schematic diagram of the experimental setup for oxygen content control.

Fig. S8. Effect of oxygen content on $FA_{0.7}MA_{0.3}Sn_{0.5}Pb_{0.5}I_3$ perovskite thin films under irradiation.

Fig. S9. The effect of light of different wavelengths and powers on the photostability of $FA_{0.7}MA_{0.3}Sn_{0.5}Pb_{0.5}I_3$ perovskite.

Fig. S10. XRD patterns of $FA_{0.7}MA_{0.3}Sn_{0.5}Pb_{0.5}I_3$ perovskite thin films in diffrernt reconstructuion stages.

Fig. S11. In situ observation of the reconstruction of $FA_{0.5}MA_{0.5}Sn_{0.5}Pb_{0.5}I_3$ thin films.

Fig. S12. PL spectra of Sn-based perovskites after irradiation.

Fig. S13. PL spectra of Pb-based perovskites after irradiation.

Fig. S14. PL spectra of aged Pb-based perovskite thin films under irradiation.

Fig. S15. The XRD and PL spectra of $FA_{0.7}MA_{0.3}Sn_xPb_{1-x}I_3$ perovskite thin films.

Fig. S16. PL spectra of $FA_{0.7}MA_{0.3}Sn_xPb_{1-x}I_3$ perovskite thin films under irradiation.

Fig. S17. Tin-lead perovskite thin films irradiation phenomena for different conditions and fractions.

Fig. S18. Progressive irradiation aging of $FA_{0.7}MA_{0.3}Sn_{0.5}Pb_{0.5}I_3$ perovskite thin films.

Fig. S19. The surface element properties of mixed tin-lead perovskite films during phase reconstruction.

Fig. S20. Surface elemental properties of mixed tin-lead cladding crystalline films after 30 min of heating at 40 °C.

Fig. S21. Surface elemental properties of mixed tin-lead cladding crystalline films under different conditions.

Fig. S22. The surfaced ions distribution of $FASn_{0.5}Pb_{0.5}I_3$ perovskite thin films in stage III (The scale bar is 100 μm).

Fig. S23. The surfaced ions distribution of $FA_{0.7}MA_{0.3}Sn_{0.5}Pb_{0.5}I_3$ perovskite thin films in different stage (The scale bar is 100 μm).

Fig. S24. The surfaced ions distribution of $FA_{0.7}MA_{0.3}Sn_{0.5}Pb_{0.5}I_3$ perovskite thin films in stage III (The scale bar is 100 μm).

Fig. S25. XRD patterns of aged $FA_{0.7}MA_{0.3}Sn_{0.5}Pb_{0.5}I_3$ perovskite thin films.

Fig. S26. Probing the reversibility of aged $FA_{0.7}MA_{0.3}Sn_{0.5}Pb_{0.5}I_3$ perovskite phase reconstruction.

Fig. S27. Effect of heating on aged $FA_{0.7}MA_{0.3}Sn_{0.5}Pb_{0.5}I_3$ perovskite thin films.

Supplementary Tables

Supplementary Table 1. Summary of photovoltaic parameters for PSCs measured in backward direction under AM 1.5G (100 mW cm^{-2}).

Supplementary Table 2. Relative distribution of elemental species for the tin-lead perovskite films in different stages.

Supplemental Items

1. Supplemental Figures

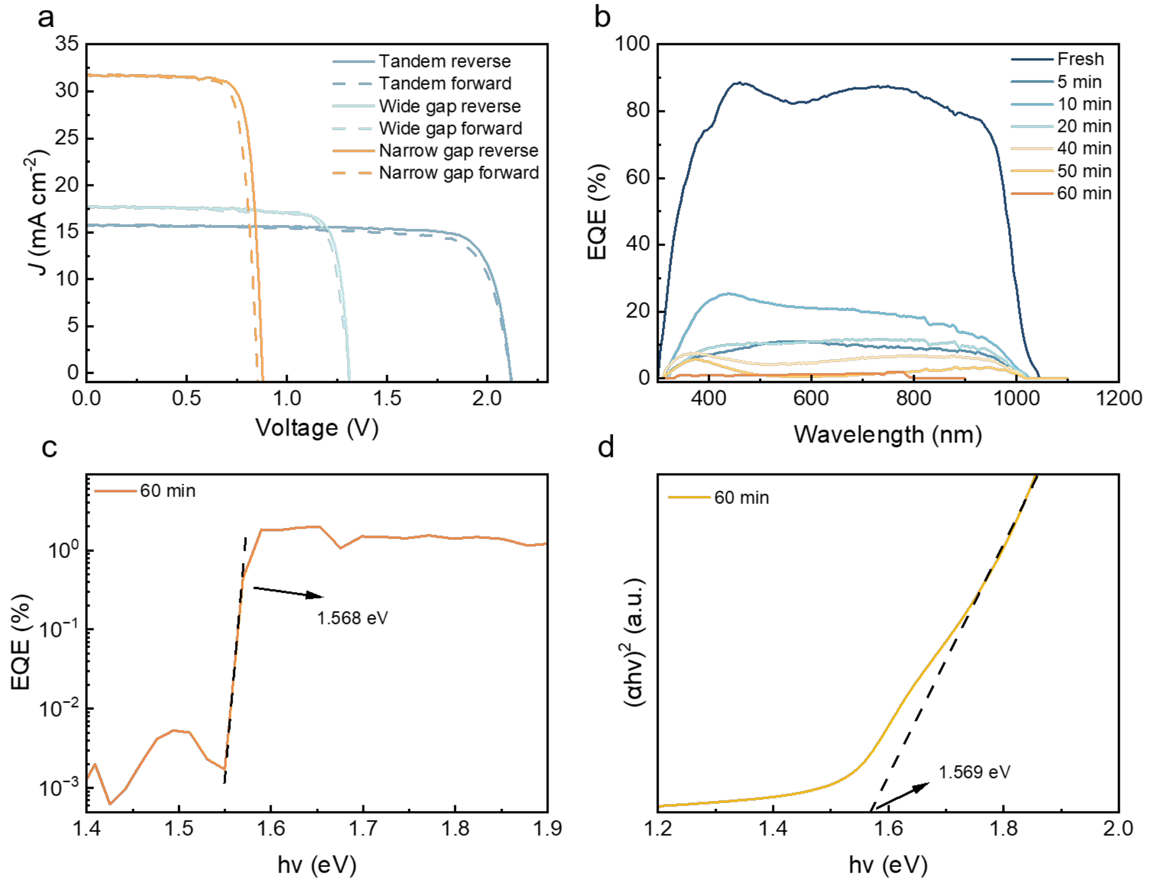

Fig. S1. Performance and optical property of different perovskite soalr cells. (a) Typical photocurrent density-voltage (*J-V*) curves of the best performing single-junction and tandem devices measured under reverse and forward voltages scan directions. (b) External quantum efficiency spectra of a single-junction narrow bandgap perovskite solar cell under irradiation/air exposure. (c) Optical band gap of a film aged for 60 min under a mercury lamp, as calculated by EQE. (d) Optical bandgap of films aged for 60 min in a mercury lamp, calculated from absorption spectra.

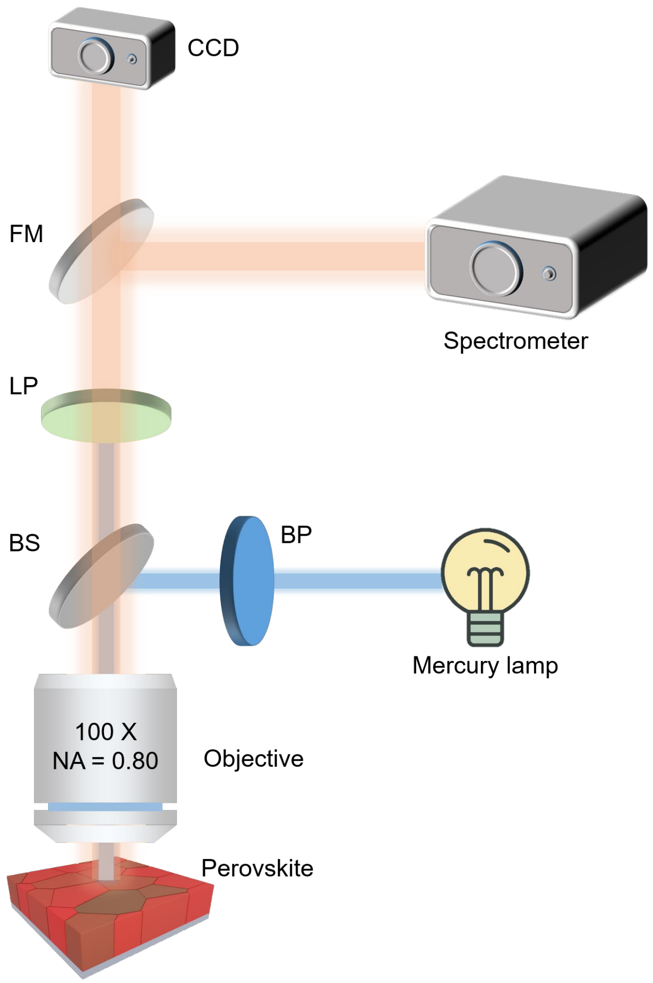

Fig. S2. Schematic of the PL microscopy experiment setup. BP: bandpass filter; BS: beam splitter; LP: long pass filter; FM: flip mirror; CCD: charge coupled device.

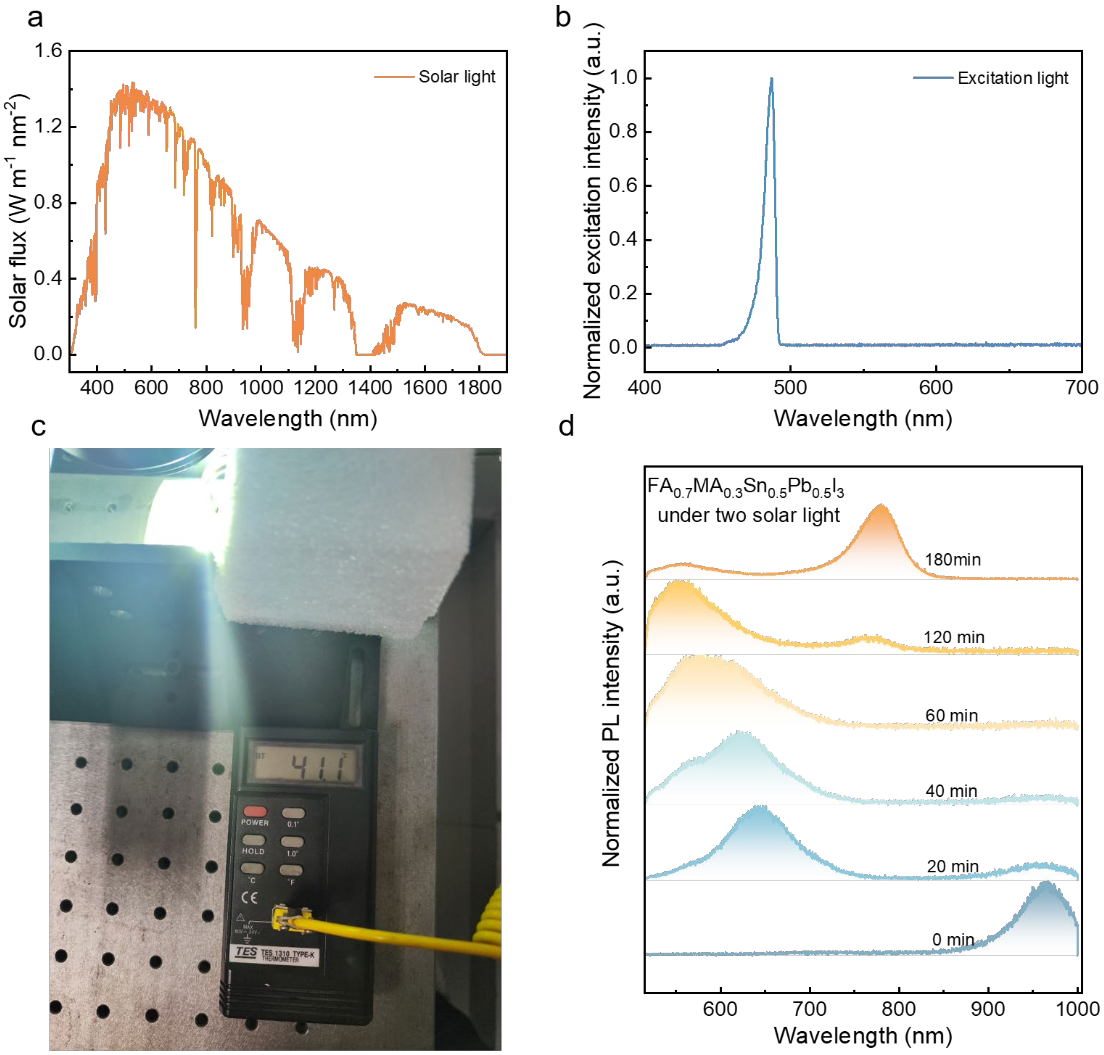

Fig. S3. Experimental conditions and phenomena under irradiation. (a) The spectrum of solar light. (b) The spectrum of irradiation light used in the experiment. (c) Films surface temperature under irradiation. (d) Evolution of normalized PL spectrum results of $FA_{0.7}MA_{0.3}Sn_{0.5}Pb_{0.5}I_3$ thin films under AM 1.5G spectra (2 sun, 200 mW cm^{-2}) with a standard solar simulator.

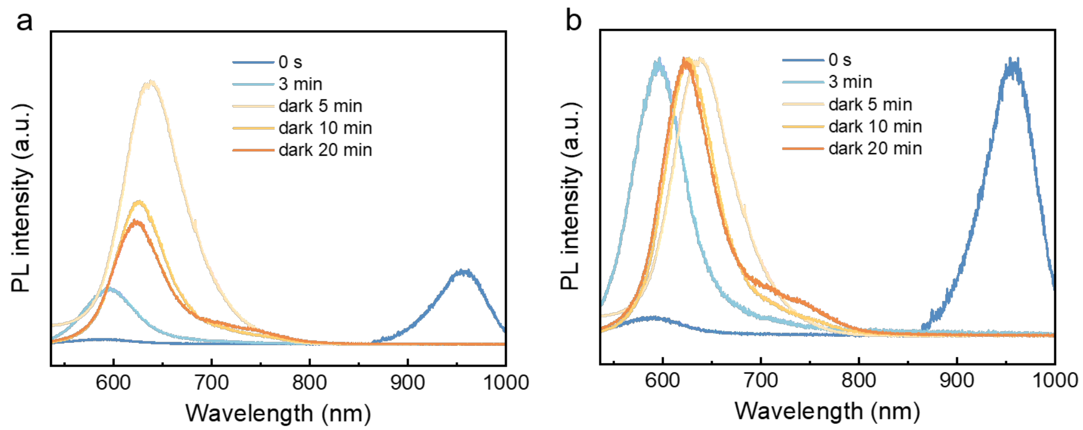

Fig. S4. Probing the reversibility of $FA_{0.7}MA_{0.3}Sn_{0.5}Pb_{0.5}I_3$ perovskite phase reconstruction. (a) PL spectra of films under dark conditions (Mercury lamp, 13 W cm^{-2}). The thin film is irradiated for 3min. (b) Normalized PL spectra of irradiated films under dark conditions (Mercury lamp, 13 W cm^{-2}).

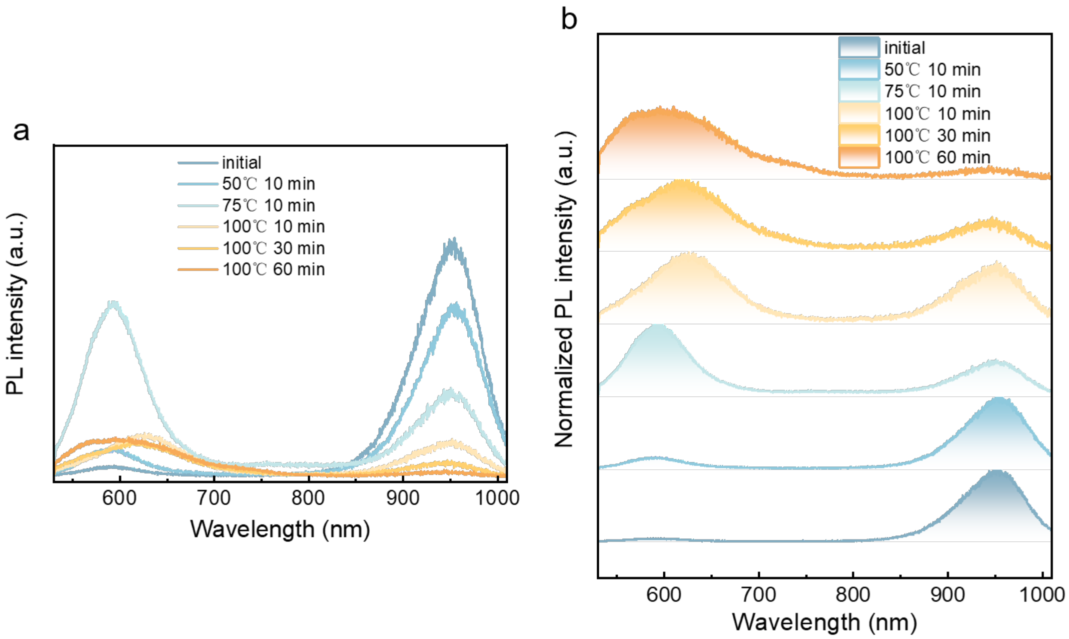

Fig. S5. Effect of heating on $FA_{0.7}MA_{0.3}Sn_{0.5}Pb_{0.5}I_3$ perovskite thin films. (a) PL spectra of $FA_{0.7}MA_{0.3}Sn_{0.5}Pb_{0.5}I_3$ perovskite films under different temperatures and times. (b) Normalized PL spectra of $FA_{0.7}MA_{0.3}Sn_{0.5}Pb_{0.5}I_3$ perovskite films under different temperatures and times.

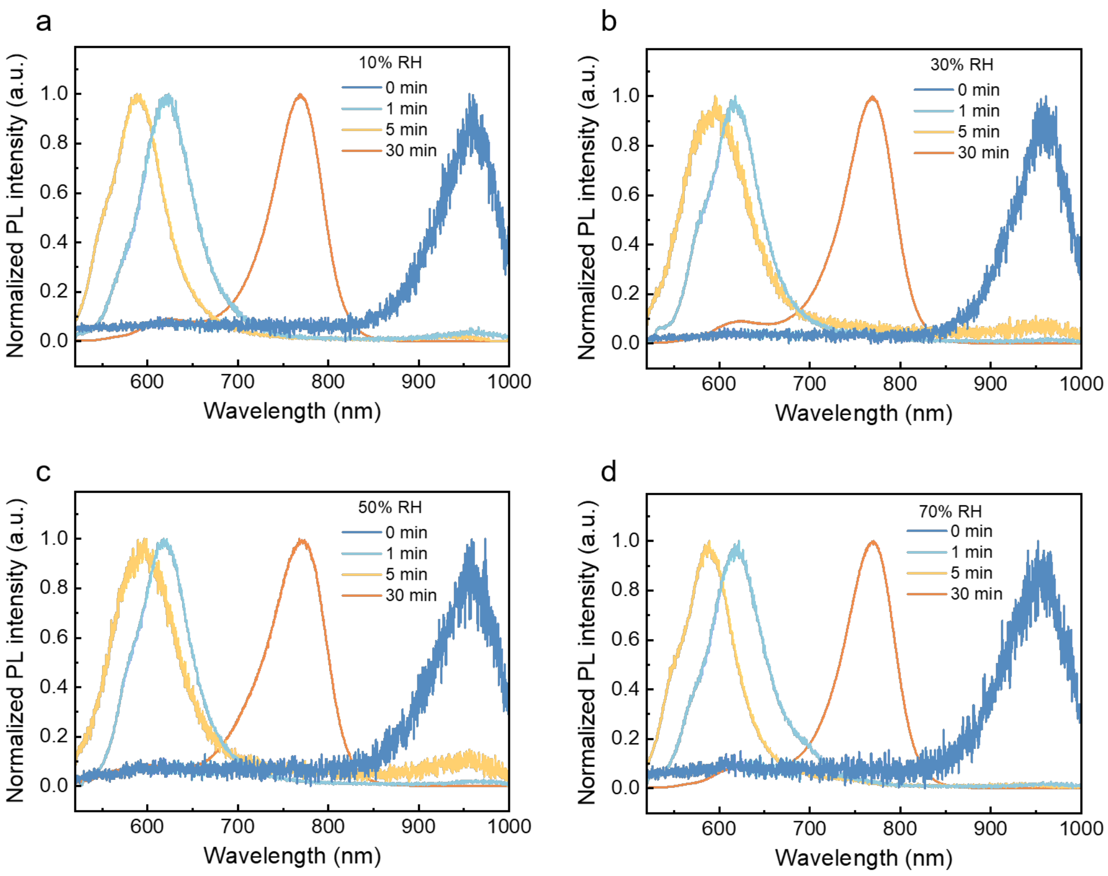

Fig. S6. Effect of moisture on $FA_{0.7}MA_{0.3}Sn_{0.5}Pb_{0.5}I_3$ perovskite thin films under irradiation. (a) Normalized PL spectra of $FA_{0.7}MA_{0.3}Sn_{0.5}Pb_{0.5}I_3$ perovskite films under 10% RH. (b) Normalized PL spectra of $FA_{0.7}MA_{0.3}Sn_{0.5}Pb_{0.5}I_3$ perovskite films under 30% RH. (c) Normalized PL spectra of $FA_{0.7}MA_{0.3}Sn_{0.5}Pb_{0.5}I_3$ perovskite films under 50% RH. (d) Normalized PL spectra of $FA_{0.7}MA_{0.3}Sn_{0.5}Pb_{0.5}I_3$ perovskite films under 70% RH.

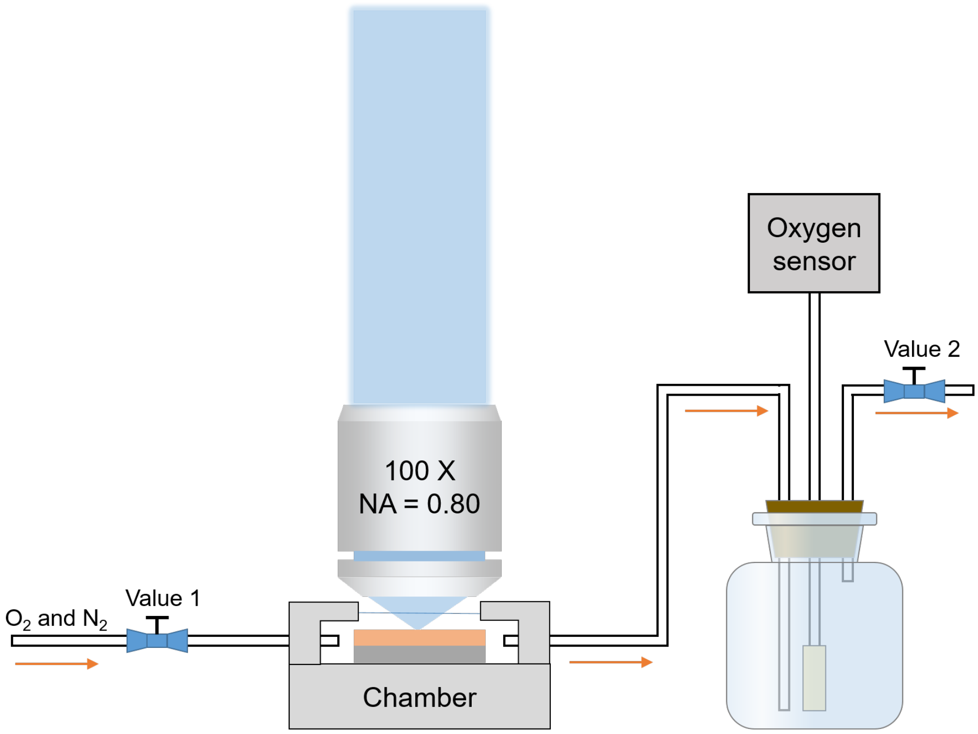

Fig. S7. Schematic diagram of the experimental setup for oxygen content control. Oxygen content is controlled by passing a mixture of gases into a closed chamber.

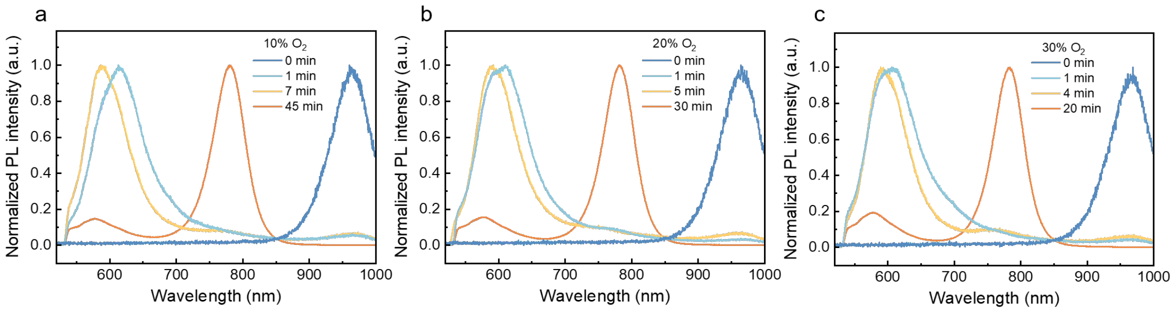

Fig. S8. Effect of oxygen content on $FA_{0.7}MA_{0.3}Sn_{0.5}Pb_{0.5}I_3$ perovskite thin films under irradiation. (a) Normalized PL spectra of $FA_{0.7}MA_{0.3}Sn_{0.5}Pb_{0.5}I_3$ perovskite films under 10% O_2. (b) Normalized PL spectra of $FA_{0.7}MA_{0.3}Sn_{0.5}Pb_{0.5}I_3$ perovskite films under 20% O_2. (c) Normalized PL spectra of $FA_{0.7}MA_{0.3}Sn_{0.5}Pb_{0.5}I_3$ perovskite films under 30% O_2.

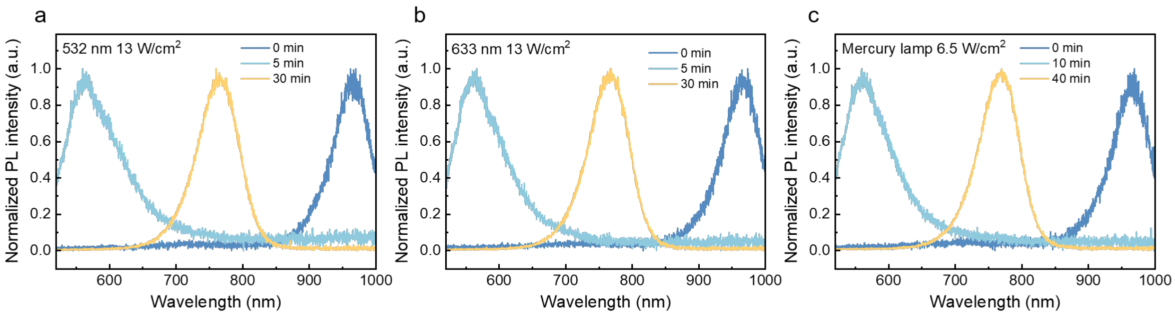

Fig. S9. The effect of light of different wavelengths and powers on the photostability of $FA_{0.7}MA_{0.3}Sn_{0.5}Pb_{0.5}I_3$ perovskite. (a) Normalized PL spectra of irradiated films under 532 nm light (532 nm light passes through a 532 nm bandpass filter). (b) Normalized PL spectra of irradiated films under 633 nm light (633 nm light passes through a 633 nm bandpass filter). (c) Normalized PL spectra of irradiated films under a Mercury lamp (Mercury lamp, 6.5 W cm^{-2}).

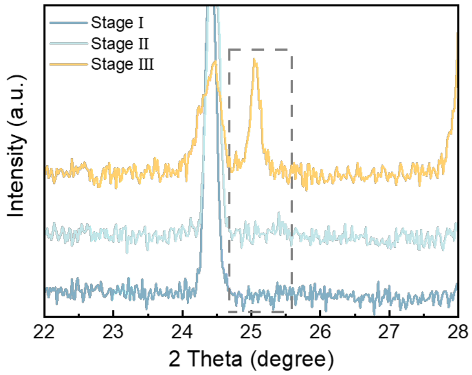

Fig. S10. XRD patterns of $FA_{0.7}MA_{0.3}Sn_{0.5}Pb_{0.5}I_3$ perovskite thin films in diffrernt reconstructuion stages. Closed-up of XRD patterns of $FA_{0.7}MA_{0.3}Sn_{0.5}Pb_{0.5}I_3$ thin films in diffrernt reconstructuion stages.

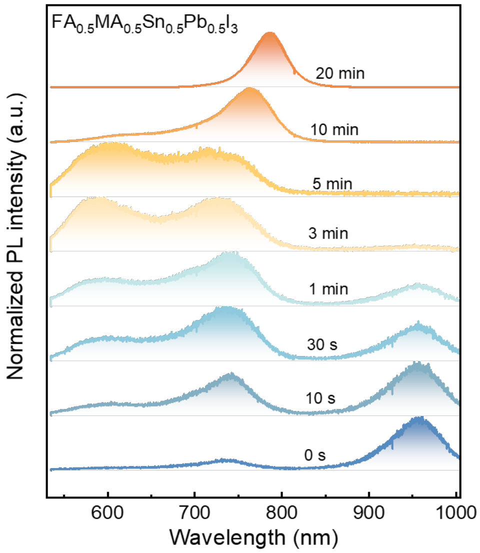

Fig. S11. In situ observation of the reconstruction of $FA_{0.5}MA_{0.5}Sn_{0.5}Pb_{0.5}I_3$ thin films. Evolution of normalized PL spectrum results of $FA_{0.5}MA_{0.5}Sn_{0.5}Pb_{0.5}I_3$ thin films under light/O_2 exposure (Mercury lamp, 13 W cm^{-2}).

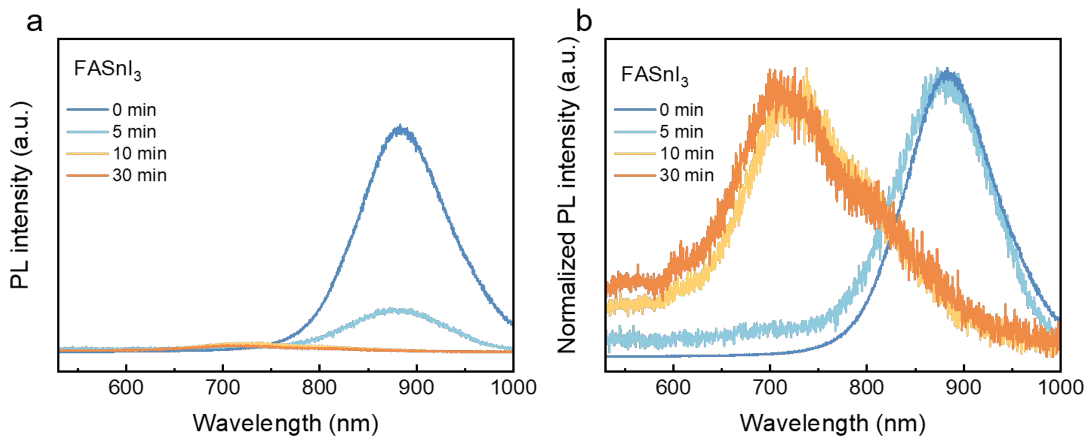

Fig. S12. PL spectra of Sn-based perovskites after irradiation. (a) Evolution of normalized PL spectrum results of $FASnI_3$ thin films under light/O_2 exposure (Mercury lamp, 13 W cm^{-2}). (b) Evolution of normalized normalized PL spectrum results of $FASnI_3$ thin films under light/O_2 exposure (Mercury lamp, 13 W cm^{-2}).

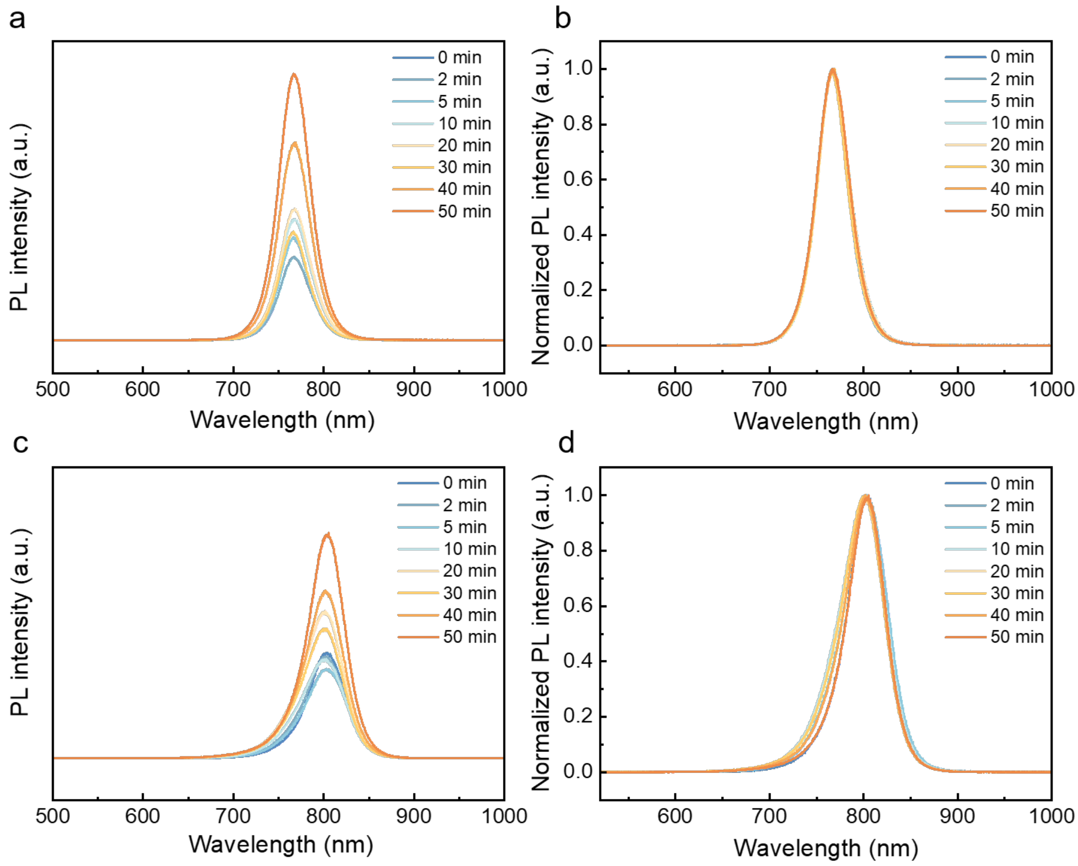

Fig. S13. PL spectra of Pb-based perovskites after irradiation. (a-b) The (a) PL and (b) normalized PL spectra of $MAPbI_3$ perovskite films under irradiation (Mercury lamp, 13 W cm^{-2}). (c-d) The (c) PL and (d) normalized PL spectra of $FAPbI_3$ perovskite films under irradiation (Mercury lamp, 13 W cm^{-2}).

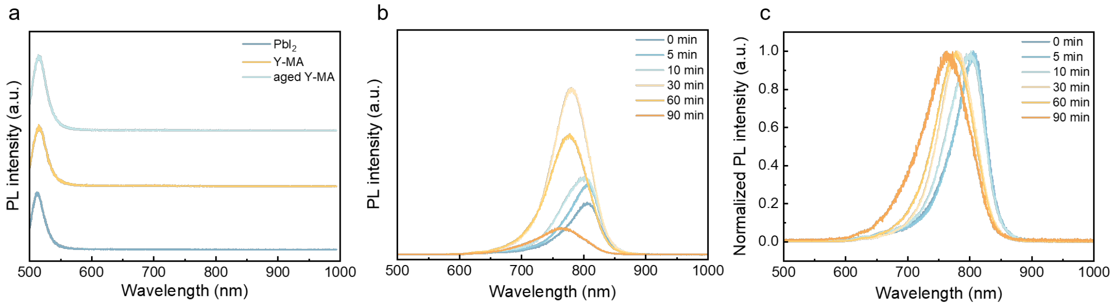

Fig. S14. PL spectra of aged Pb-based perovskite thin films under irradiation. (a) The PL spectra of air-aged $MAPbI_3$ perovskite films under irradiation (Mercury lamp, 13 W cm^{-2}). (b-c) The (b) PL and (c) normalized spectra of air-aged $FAPbI_3$ perovskite films under irradiation (Mercury lamp, 13 W cm^{-2}).

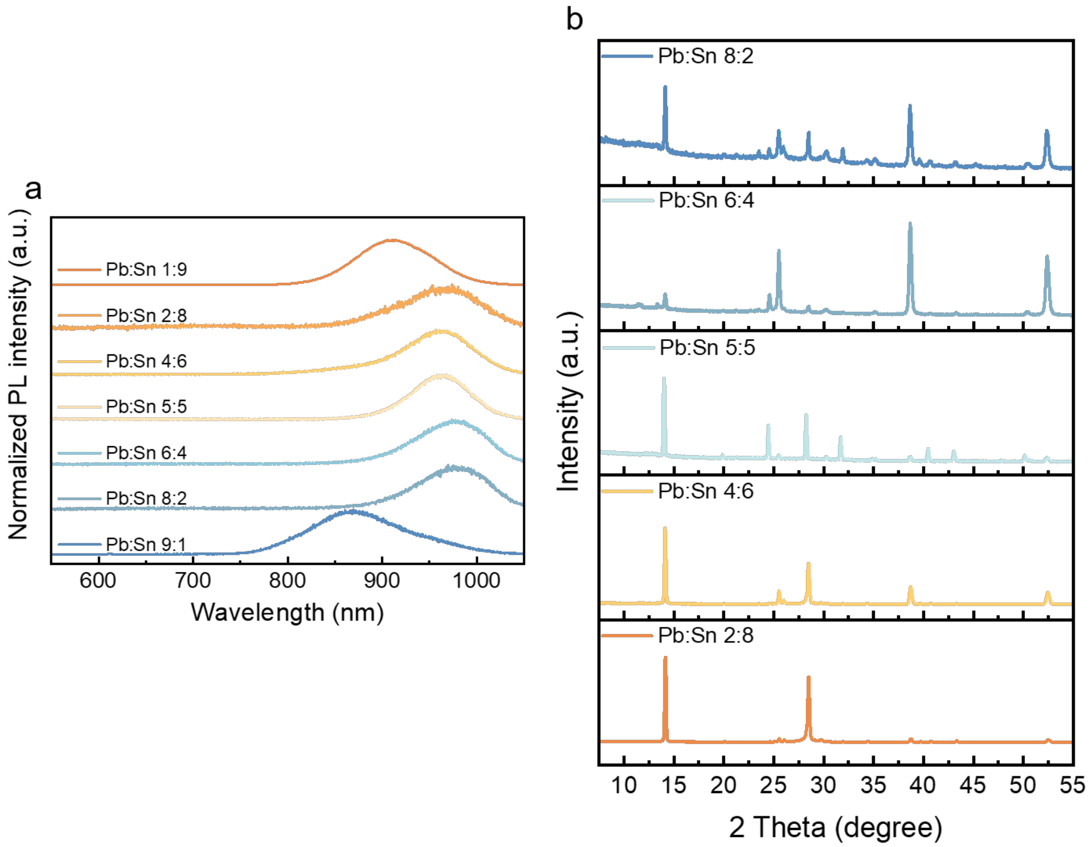

Fig. S15. The XRD and PL spectra of $FA_{0.7}MA_{0.3}Sn_xPb_{1-x}I_3$ perovskite thin films. (a) Steady-state PL spectra of $FA_{0.7}MA_{0.3}Sn_xPb_{1-x}I_3$ thin films on the cover glass. (b) XRD patterns of $FA_{0.7}MA_{0.3}Sn_xPb_{1-x}I_3$ thin films on the cover glass.

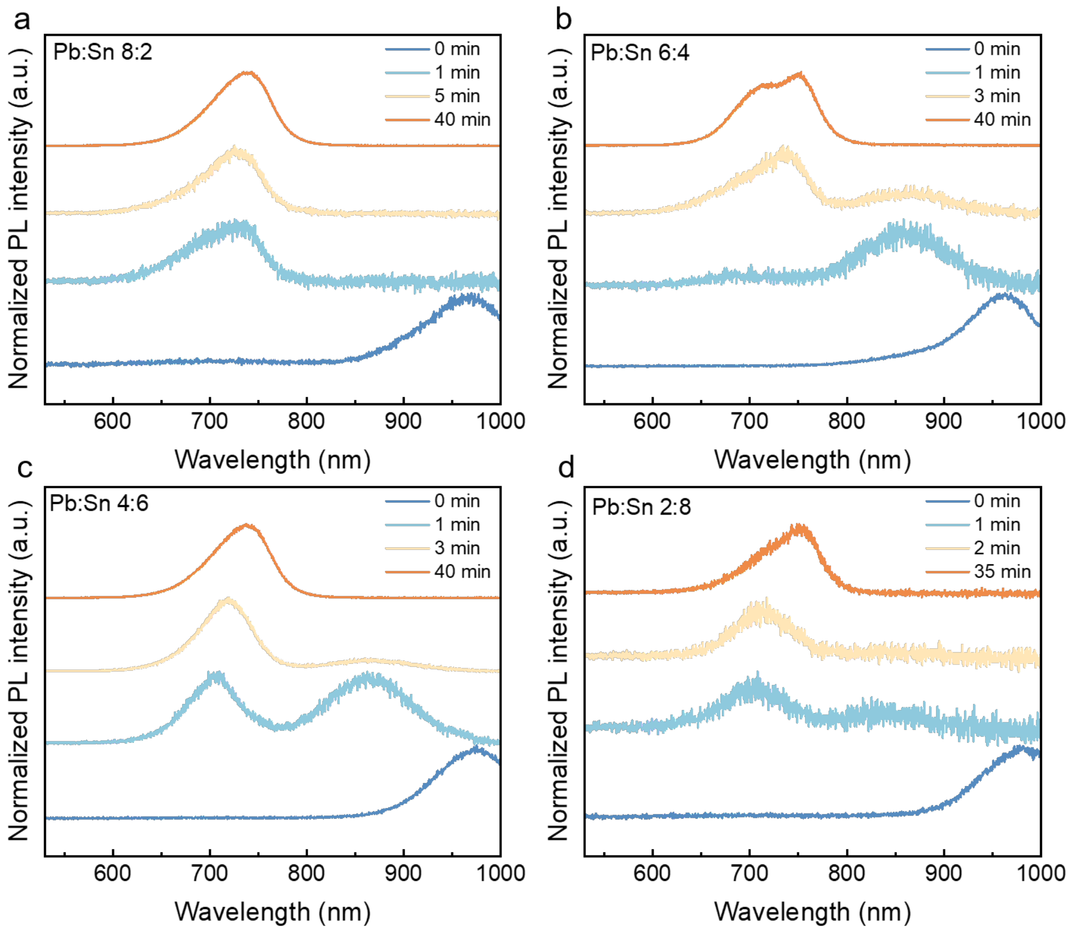

Fig. S16. PL spectra of $FA_{0.7}MA_{0.3}Sn_xPb_{1-x}I_3$ perovskite thin films under irradiation. (a) The PL spectra of $FA_{0.7}MA_{0.3}Sn_{0.2}Pb_{0.8}I_3$ perovskite films under irradiation (Mercury lamp, 13 W cm^{-2}). (b) The PL spectra of $FA_{0.7}MA_{0.3}Sn_{0.4}Pb_{0.6}I_3$ perovskite films under irradiation (Mercury lamp, 13 W cm^{-2}). (c) The PL spectra of $FA_{0.7}MA_{0.3}Sn_{0.6}Pb_{0.4}I_3$ perovskite films under irradiation (Mercury lamp, 13 W cm^{-2}). (d) The PL spectra of $FA_{0.7}MA_{0.3}Sn_{0.8}Pb_{0.2}I_3$ perovskite films under irradiation (Mercury lamp, 13 W cm^{-2}).

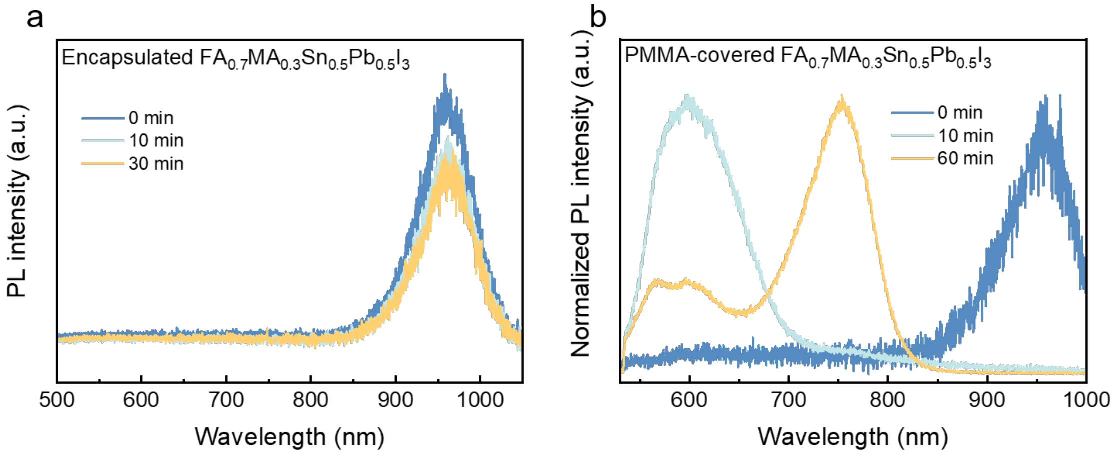

Fig. S17. Tin-lead perovskite thin films irradiation phenomena for different conditions and fractions. (a) Evolution of normalized PL spectrum results of encapsulated $FA_{0.7}MA_{0.3}Sn_{0.5}Pb_{0.5}I_3$ thin films under irradiation (Mercury lamp, 13 W cm^{-2}). (b) Evolution of normalized PL spectrum results of PMMA modified $FA_{0.7}MA_{0.3}Sn_{0.5}Pb_{0.5}I_3$ thin films under irradiation (Mercury lamp, 13 W cm^{-2}).

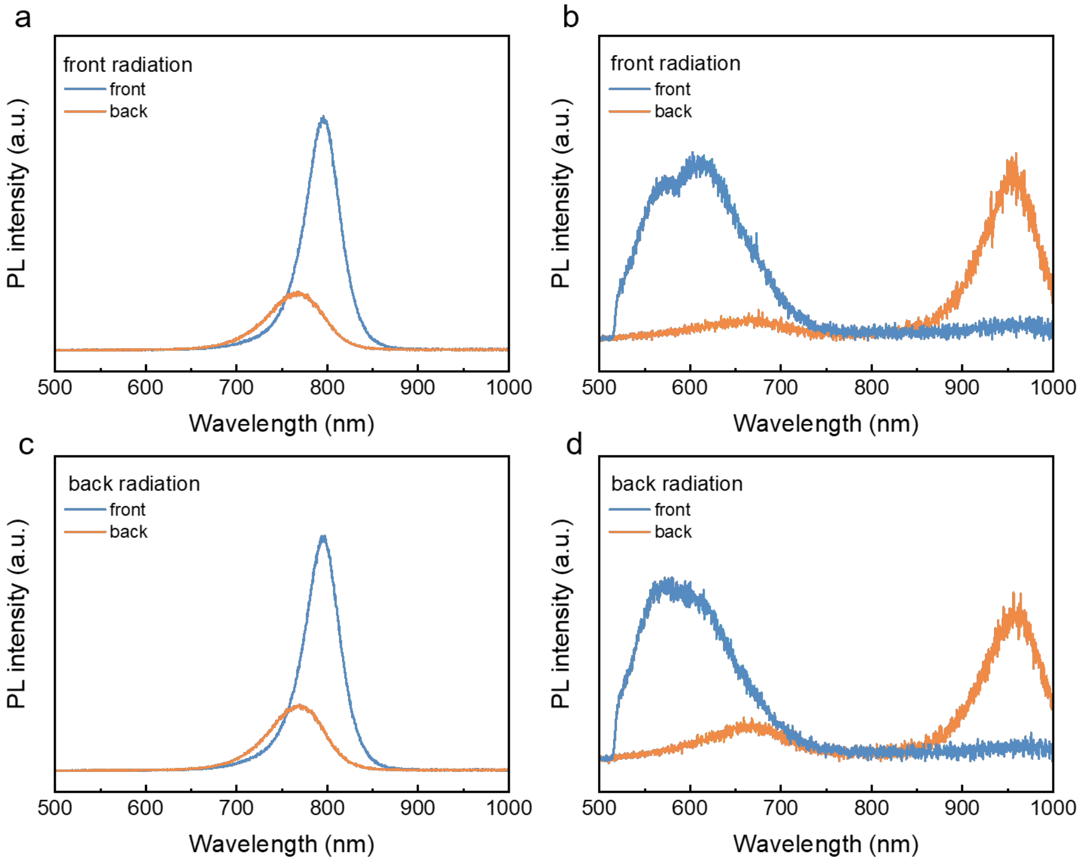

Fig. S18. Progressive irradiation aging of $FA_{0.7}MA_{0.3}Sn_{0.5}Pb_{0.5}I_3$ perovskite thin films. (a-b) The PL spectra of $FA_{0.7}MA_{0.3}Sn_{0.5}Pb_{0.5}I_3$ perovskite films in (A) stage III and (B) stage II from the front and back side under films front irradiation. (c-d) The PL spectra of $FA_{0.7}MA_{0.3}Sn_{0.5}Pb_{0.5}I_3$ perovskite films in (C) stage III and (D) stage II from the front and back side under films back irradiation.

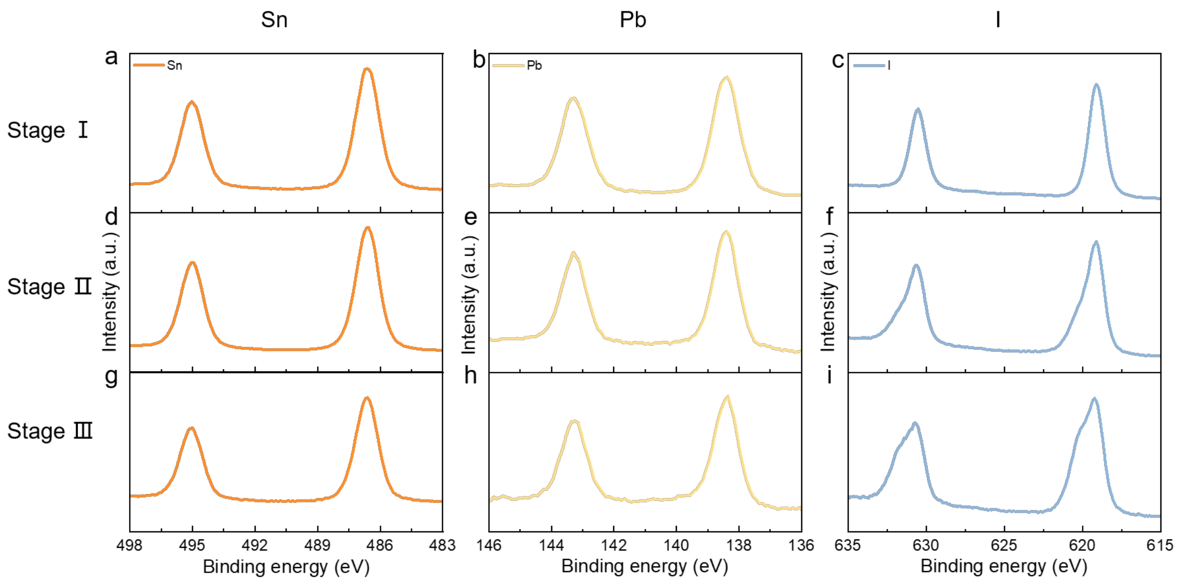

Fig. S19. The surface element properties of mixed tin-lead perovskite films during phase reconstruction. (a-c) Complete XPS spectra of mixed $FA_{0.7}MA_{0.3}Sn_{0.5}Pb_{0.5}I_3$ perovskite films in stage I: (a) Sn $3d_{5/2}$; (b) Pb $4f_{7/2}$; (c) I $3d_{5/2}$. (d-f) Complete XPS spectra of mixed $FA_{0.7}MA_{0.3}Sn_{0.5}Pb_{0.5}I_3$ perovskite films in stage II: (d) Sn $3d_{5/2}$; (e) Pb $4f_{7/2}$; (f) I $3d_{5/2}$. (g-i) Complete XPS spectra of mixed $FA_{0.7}MA_{0.3}Sn_{0.5}Pb_{0.5}I_3$ perovskite films in stage III: (g) Sn $3d_{5/2}$; (h) Pb $4f_{7/2}$; (i) I $3d_{5/2}$.

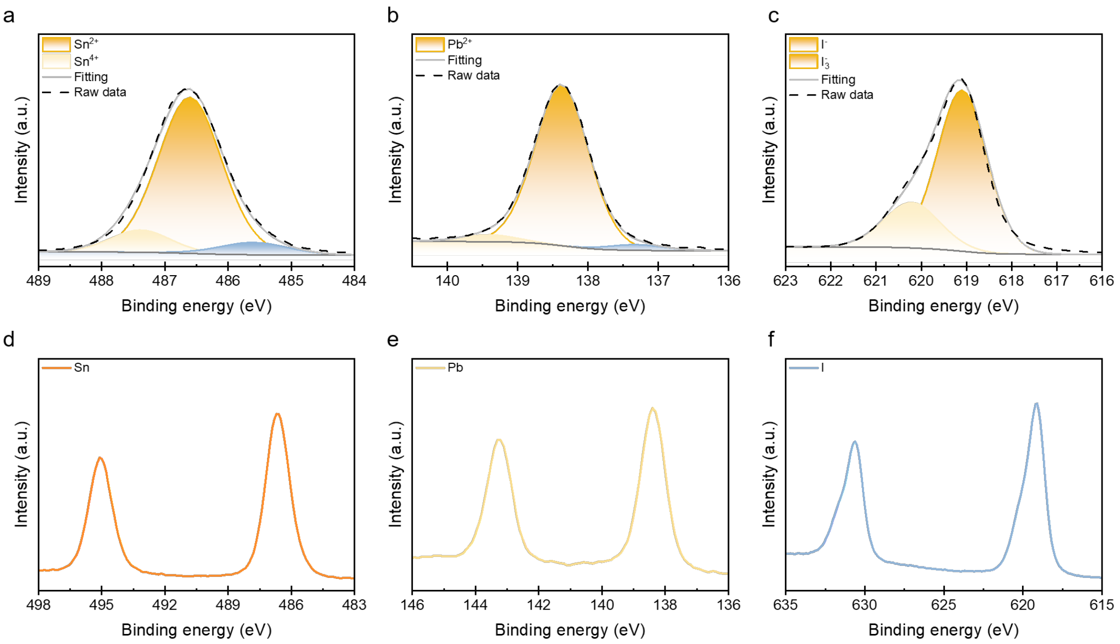

Fig. S20. Surface elemental properties of mixed tin-lead cladding crystalline films after 30 min of heating at 40 °C. (a-c) XPS spectra of mixed $FA_{0.7}MA_{0.3}Sn_{0.5}Pb_{0.5}I_3$ perovskite films after 30 min of heating at 40 °C: (a) Sn $3d_{5/2}$; (b) Pb $4f_{7/2}$; (c) I $3d_{5/2}$. (d-f) Complete XPS spectra of mixed $FA_{0.7}MA_{0.3}Sn_{0.5}Pb_{0.5}I_3$ perovskite films after 30 min of heating at 40 °C: (d) Sn $3d_{5/2}$; (e) Pb $4f_{7/2}$; (f) I $3d_{5/2}$.

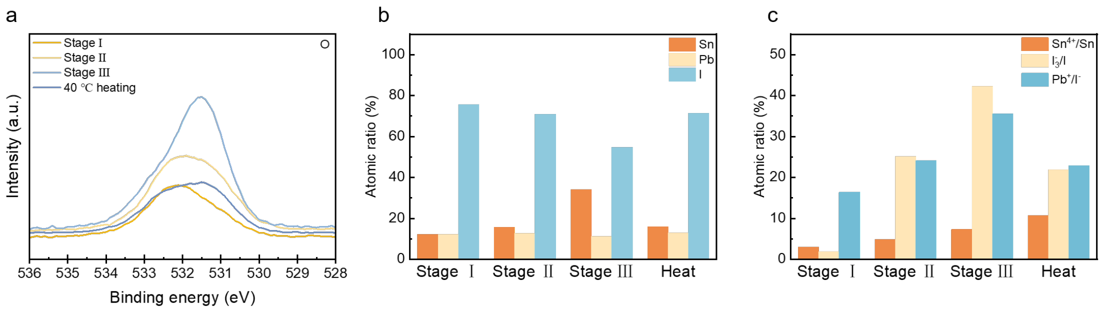

Fig. S21. Surface elemental properties of mixed tin-lead cladding crystalline films under different conditions. (a) Complete XPS spectra of O of mixed $FA_{0.7}MA_{0.3}Sn_{0.5}Pb_{0.5}I_3$ perovskite films under different conditions. (b) Proportion of elemental content on the surface of mixed $FA_{0.7}MA_{0.3}Sn_{0.5}Pb_{0.5}I_3$ crystal films under different conditions. (c) Proportion of different valence content of elements on the surface of mixed $FA_{0.7}MA_{0.3}Sn_{0.5}Pb_{0.5}I_3$ crystal films under different conditions.

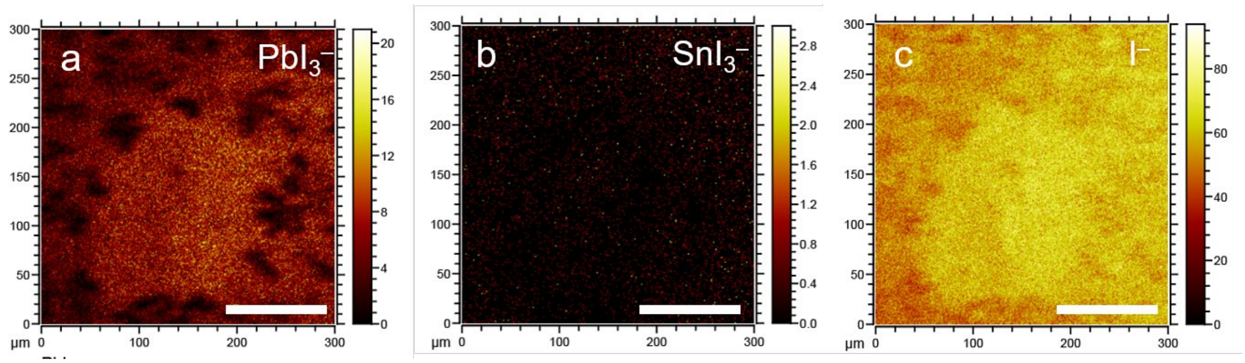

Fig. S22. The surfaced ions distribution of $FASn_{0.5}Pb_{0.5}I_3$ perovskite thin films in stage III (The scale bar is 100 μm). (a-c) TOF-SIMS mapping signals of (a) PbI_3^-, (b) SnI_3^-, (c) I^- on the mixed $FASn_{0.5}Pb_{0.5}I_3$ perovskite films in stage III.

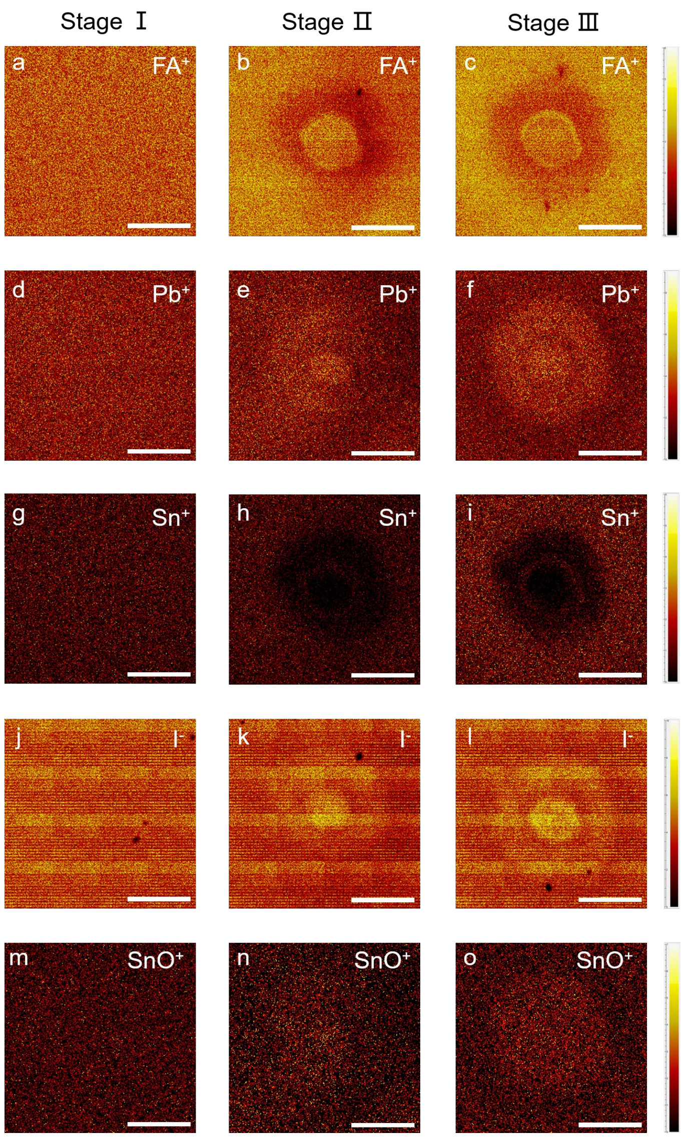

Fig. S23. The surfaced ions distribution of $FA_{0.7}MA_{0.3}Sn_{0.5}Pb_{0.5}I_3$ perovskite thin films in different stage (The scale bar is 100 µm). (a-c) TOF-SIMS mapping signals of FA^+ on the mixed $FA_{0.7}MA_{0.3}Sn_{0.5}Pb_{0.5}I_3$ perovskite films in different stage . (d-f) TOF-SIMS mapping signals of Pb^+ on the mixed $FA_{0.7}MA_{0.3}Sn_{0.5}Pb_{0.5}I_3$ perovskite films in different stage . (g-i) TOF-SIMS mapping signals of Sn^+ on the mixed $FA_{0.7}MA_{0.3}Sn_{0.5}Pb_{0.5}I_{33}$ perovskite films in different stage . (j-l) TOF-SIMS mapping signals of I^- on the mixed $FA_{0.7}MA_{0.3}Sn_{0.5}Pb_{0.5}I_3$ perovskite films in different stage . (m-o) TOF-SIMS mapping signals of SnO^+ on the mixed $FA_{0.7}MA_{0.3}Sn_{0.5}Pb_{0.5}I_3$ perovskite films in different stage .

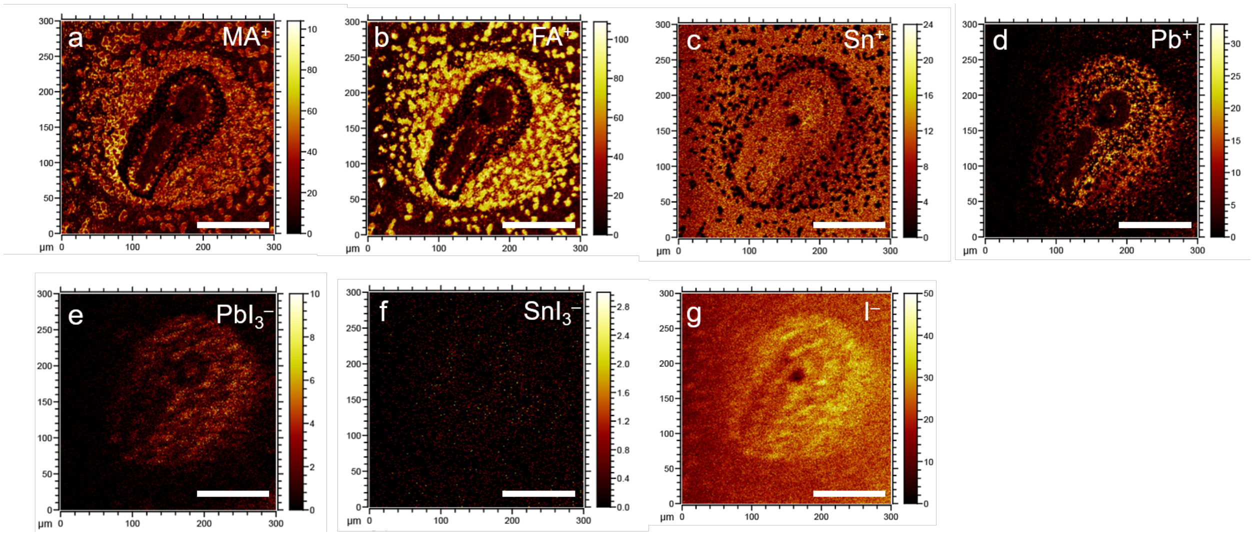

Fig. S24. The surfaced ions distribution of $FA_{0.7}MA_{0.3}Sn_{0.5}Pb_{0.5}I_3$ perovskite thin films in stage III (The scale bar is 100 μm). (a-g) TOF-SIMS mapping signals of (a) MA^+, (b) FA^+, (c) Sn^+, (d) Pb^+, (e) PbI_3^-, (f) SnI_3^-, (g) I^- on the mixed $FA_{0.7}MA_{0.3}Sn_{0.5}Pb_{0.5}I_3$ perovskite films in stage III.

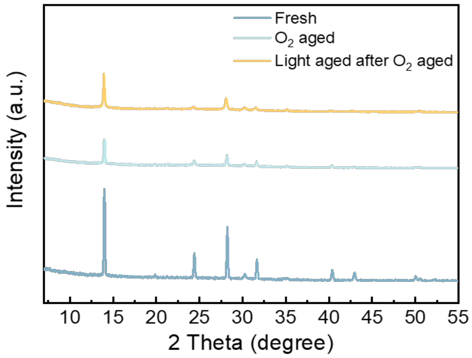

Fig. S25. XRD patterns of aged $FA_{0.7}MA_{0.3}Sn_{0.5}Pb_{0.5}I_3$ perovskite thin films. The XRD of the fresh $FA_{0.7}MA_{0.3}Sn_{0.5}Pb_{0.5}I_3$ perovskite films, the films with air exposure, and the films with continuous blue-light irradiation after air exposure.

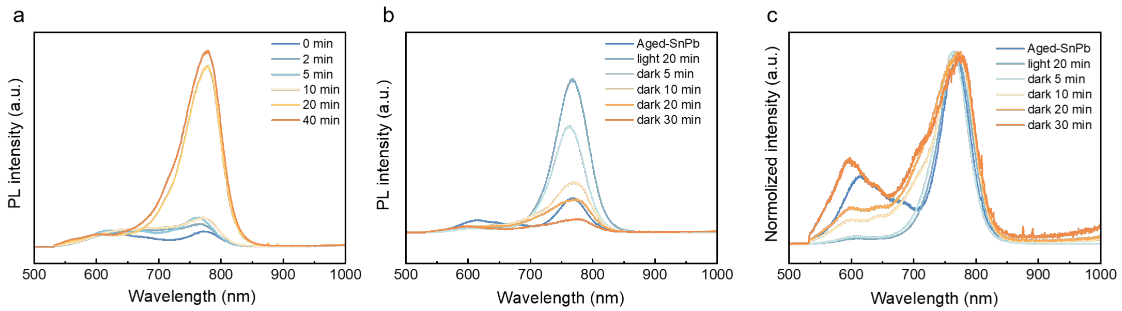

Fig. S26. Probing the reversibility of aged $FA_{0.7}MA_{0.3}Sn_{0.5}Pb_{0.5}I_3$ perovskite phase reconstruction. (a) PL spectra of irradiation of aged films. (b) PL spectra of irradiation of aged films under dark conditions. (c) Normalized PL spectra of irradiation of aged films under dark conditions.

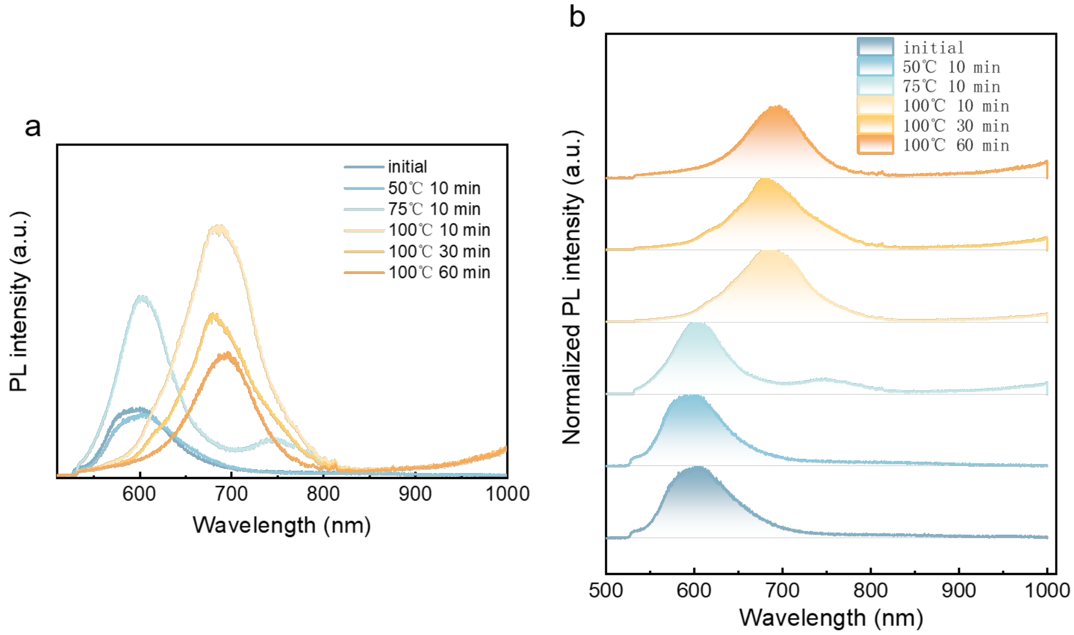

Fig. S27. Effect of heating on aged $FA_{0.7}MA_{0.3}Sn_{0.5}Pb_{0.5}I_3$ perovskite thin films. (a) PL spectra of aged $FA_{0.7}MA_{0.3}Sn_{0.5}Pb_{0.5}I_3$ perovskite films under different temperatures and times. (b) Normalized PL spectra of aged $FA_{0.7}MA_{0.3}Sn_{0.5}Pb_{0.5}I_3$ perovskite films under different temperatures and times.

2. Supplemental Tables

Table S1. Summary of photovoltaic parameters for PSCs measured in backward direction under AM 1.5G (100 mW cm^{-2}).

Devices	J_{sc} (mA cm^{-2})	V_{oc} (V)	FF (%)	PCE (%)
2T tandem-reverse	15.74	2.11	81.98	27.28
2T tandem-forward	15.80	2.11	77.91	26.03
WBG-reverse	17.71	1.30	81.82	18.82
WBG-forward	17.68	1.30	80.65	18.49
NBG-reverse	31.76	0.87	80.36	22.37
NBG-forward	31.88	0.85	78.63	21.36

Table S2. Relative distribution of elemental species for the tin-lead perovskite films in different stages.

		Binding energy (eV)	Stage I (%)	Stage II (%)	Stage III (%)	Heat (%)
total Sn						
	Sn^{2+}	486.6	96.8	94.7	91.9	82.6
	Sn^{4+}	487.2	3.0	4.8	7.3	10.7
	Sn (0)	485.3	0.2	0.5	0.8	6.6
total Pb						
	Pb^{2+}	138..4	98.3	97.3	97.3	92.6
	$PbCO_3$	139.4	0.8	1.3	1.2	3.7
	Sn 4s	137.2	0.9	1.4	1.5	3.7
total I						
	I^-	619.1	94.6	72.4	54.8	78.1
	I^{3-}	620.2	1.9	25.1	42.3	21.9
