# Supplementary material for: Unveiling the nexus between irradiation and phase reconstruction in tin-lead perovskite solar cells
Source: Nat Commun. 2025 Jan 8;16:506. doi: 10.1038/s41467-025-55814-0 (PMC11711683; doi:10.1038/s41467-025-55814-0)
Supplement: Supplementary file 2 — Reporting Summary [file 41467_2025_55814_MOESM2_ESM.pdf]

nature portfolio

Corresponding author(s): Weijun Ke, Ti Wang, and Hongxing Xu

Solar Cells Reporting Summary

Nature Portfolio wishes to improve the reproducibility of the work that we publish. This form is intended for publication with all accepted papers reporting the characterization of photovoltaic devices and provides structure for consistency and transparency in reporting. Some list items might not apply to an individual manuscript, but all fields must be completed for clarity.

For further information on Nature Research policies, including our data availability policy, see Authors & Referees.

Experimental design

Please check the following details are reported in the manuscript, and provide a brief description or explanation where applicable.

1. Dimensions

Area of the tested solar cells

- [x] Yes — Methods Section, Device performance measurements.
- [ ] No — *Explain why this information is not reported/not relevant.*

Method used to determine the device area

- [x] Yes — Methods Section, Device performance measurements.
- [ ] No — *Explain why this information is not reported/not relevant.*

2. Current-voltage characterization

Current density-voltage (J-V) plots in both forward and backward direction

- [x] Yes — Figure 1A, Supplemental Table S1, Supplemental Figure S1
- [ ] No

Voltage scan conditions

- [x] Yes — Methods Section, Device performance measurements.
- [ ] No — *Explain why this information is not reported/not relevant.*

Test environment

- [x] Yes — In an N2-filled glove box at room temperature.
- [ ] No — *Explain why this information is not reported/not relevant.*

Protocol for preconditioning of the device before its characterization

- [ ] Yes — *Provide a description of the protocol.*
- [x] No — No preconditioning is required for our cell characterization.

Stability of the J-V characteristic

- [x] Yes — Figure 1B
- [ ] No — *Explain why this information is not reported/not relevant.*

3. Hysteresis or any other unusual behaviour

Description of the unusual behaviour observed during the characterization

- [x] Yes — Negligible hysteresis was found.
- [ ] No — *Explain why this information is not reported/not relevant.*

Related experimental data

- [x] Yes — J-V curves under reverse and forward scans are provided.
- [ ] No — *Explain why this information is not reported/not relevant.*

4. Efficiency

External quantum efficiency (EQE) or incident photons to current efficiency (IPCE)

- [x] Yes — EQE curves are provided in Figure 1C.
- [ ] No — *Explain why this information is not reported/not relevant.*

A comparison between the integrated response under the standard reference spectrum and the response measure under the simulator

- [x] Yes — The integrated Jsc values obtained from EQE agree well with the Jsc determined from the J–V measurements.
- [ ] No — *Explain why this information is not reported/not relevant.*

April 2023

For tandem solar cells, the bias illumination and bias voltage used for each subcell

☒ Yes — Methods Section, Device performance measurements.
☐ No — *Explain why this information is not reported/not relevant.*

5. Calibration

Light source and reference cell or sensor used for the characterization

☒ Yes — Methods Section, Device performance measurements.
☐ No — *Explain why this information is not reported/not relevant.*

Confirmation that the reference cell was calibrated and certified

☒ Yes — The light intensity was set at 100 mW cm−2 and calibrated with a certified WPVS standard solar reference cell (SRC-2020, Enlitech; traceable to NREL) before measurements.
☐ No — *Explain why this information is not reported/not relevant.*

Calculation of spectral mismatch between the reference cell and the devices under test

☐ Yes — *Provide a value of the spectral mismatch and/or a description of how it has been taken into account in the measurements.*
☒ No — The light spectrum used for measurements matches well with the reference silicon cell, and we did not calculate the spectral mismatch between the reference cell and the tested devices.

6. Mask/aperture

Size of the mask/aperture used during testing

☒ Yes — The area of the aperture mask was 0.070225 cm2.
☐ No — *Explain why this information is not reported/not relevant.*

Variation of the measured short-circuit current density with the mask/aperture area

☐ Yes — *Report the difference in the short-circuit current density values measured with the mask and aperture area.*
☒ No — Negligible

7. Performance certification

Identity of the independent certification laboratory that confirmed the photovoltaic performance

☐ Yes — *Identify the independent certification laboratory.*
☒ No — This work is mainly devoted to the study of mechanism

A copy of any certificate(s)

☐ Yes — *Certificate copies should be provided in the Supplementary information. Please state the supplementary item number.*
☒ No — This work is mainly devoted to the study of mechanism

8. Statistics

Number of solar cells tested

☐ Yes — *Report how many solar cells have been tested, specifying the number of individual substrates.*
☒ No — This work does not focus on the repeatability of device performance

Statistical analysis of the device performance

☐ Yes — *State where this information can be found in the text.*
☒ No — This work does not focus on the repeatability of device performance

9. Long-term stability analysis

Type of analysis, bias conditions and environmental conditions

☒ Yes — Figure 1B
☐ No — under continue blue light soaking; no bias; at room temperature; in ambient air;
