# Supplementary material for: Unveiling the nexus between irradiation and phase reconstruction in tin-lead perovskite solar cells
Source: Nat Commun. 2025 Jan 8;16:506. doi: 10.1038/s41467-025-55814-0 (PMC11711683; doi:10.1038/s41467-025-55814-0)
Supplement: Supplementary file 3 — Peer Review file [file 41467_2025_55814_MOESM3_ESM.pdf]

Peer Review File

Unveiling the nexus between irradiation and phase reconstruction in tin-lead perovskite solar cells

Corresponding Author: Professor Ti Wang

This file contains all reviewer reports in order by version, followed by all author rebuttals in order by version.

Version 0:

Reviewer comments:

Reviewer #1

(Remarks to the Author)
This manuscript unveils a critical degradation mechanism of Sn-Pb perovskite under irradiation/air exposure. The proposed phase reconstruction process of Sn-Pb perovskite has been supported by full explanations and various characterizations. The superoxide generated from irradiation/air exposure drives the oxidation of Sn^{2+} to form Sn vacancies, causing the increase of Pb-rich regions to facilitate the random generation of black-phase perovskite from yellow-phase perovskite. This work is of great interest and significance to the community of this field, which provides a new perspective to improve the long-term stability of Sn-Pb PSCs. Therefore, I would recommend acceptance of this manuscript in Nature Communications after addressing the following minor issues/comments.
1. One of the enlightenments from this work is somewhat confusing. For Sn-Pb perovskites, even though an iodide-rich environment can certainly reduce the formation of iodide vacancies, it simultaneously increases the amounts of tin vacancies which are deemed as the main reason impairing the performance of Sn-Pb PSCs. Thus, the strategy of exploring an iodide-rich environment to prevent iodide vacancies for Sn-Pb perovskites seems inappropriate. Please make corresponding modifications or provide explanations.
2. Is the phase reconstruction process correlated with the light wavelength emitted by the excitation illumination?
3. The authors claim that Sn-Pb perovskite solar cells undergo phase reconstruction during irradiation in Fig. 1c. However, the shift in phase reconstruction after an irradiation duration of more than 40 min is abrupt. It is suggested to characterize devices with irradiation durations between 40 and 60 min. This could offer better understanding into the reconstruction process.
4. The migration and aggregation of Sn elements (or Sn vacancies) proposed by the authors seem to have weak reliability based solely on SIMS results. The authors should supplement characterizations and explanations on this.
5. Figs. 1d and 2a do not have the identical time scales for Sn-Pb perovskite phase reconstruction process. Please provide a detailed description about this.
6. The authors selected two typical Sn-Pb perovskite compounds for investigation: $FA_{0.7}MA_{0.3}Sn_{0.5}Pb_{0.5}I_3$ and MA-free $FASn_{0.5}Pb_{0.5}I_3$, and they demonstrated that the superoxide degradation mechanism of Sn-Pb perovskites is different from their pure-Pb and pure-Sn counterparts. Furthermore, I am curious about whether this degradation mechanism of Sn-Pb perovskites varies with the ratio of Sn to Pb?
7. The chemistry of the surface ions of the films at three different reconstruction stages is shown in Figure 3. Please provide complete XPS mapping for a full comparison of the results.
8. Please correct the mistake writing of “I^- and Sn^{2+} vacancies”.

Reviewer #2

(Remarks to the Author)
In their work, the authors are addressing a crucial topic of low-bandgap perovskite solar cells by investigating the phase transition processes in mixed Pb-Sn perovskites under air and light exposure. By doing so, the authors find fundamental limitations of that material class, generate new insights as well as learnings for future improvement of the operational stability of this interesting material class. The initial and final sections of the manuscript are written very well. However, some sections in between are bit hard to follow as their connections to the previous and following discussions are not fully clear. I would like to ask the authors to please work on clarifying the connections between the different investigations a bit better before the manuscript can be accepted. In addition, clarification is required on the following aspects:

On page 7 the authors write “However, the edge shifting to 800 nm indicates the formation of a new perovskite phase during the aging process.” It is not clear from these simple measurements that a “perovskite” phase is forming. It is also not clear that only “one” phase is forming. The authors should either proof their statements here or reword the sentence.

On page 8 the authors indicate that the illumination with AM1.5 spectrum and mercury spectrum result in similar effects and conclude that the blue light drives the degradation. Can the authors please further check that by using different filters that remove more or less parts within the wavelength range around 475 nm and investigating the degradation mechanism of the UV filtered AM1.5 spectrum that the degradation is indeed changing (i.e., is the degradation less pronounced without UV)?

On page 8 the authors write “While one might expect the unstable tin-lead mixed perovskites to degrade under continuous irradiation, they instead transition to the black phase of $FAPbI_3$”. That sentence is misleading as the perovskite did degrade. It does not matter if the final state is again another perovskite phase, the initial phase was not stable and degraded into a more stable other phase, i.e., it degraded. Please reword.

In some of the initial films (e.g. Fig. 2, S2 and S3) it looks like a tiny amount of the “600 nm” phase is already present. Can the authors please comment on that observation? Where does that phase come from and is this phase responsible for the initial degradation? Can an improved phase purity improve the operational stability of the authors’ devices?

On page 10 the authors write “At Stage I, the XRD patterns confirm a pure phase of $FA_{0.7}MA_{0.3}Sn_{0.5}Pb_{0.5}I_3$”, which doesn’t seem correct based on my comment from above. PL shows that there is already a tiny bit of a secondary phase in the initial films. Please clarify.

On page 11 the authors describe the phase dynamics of $FA_{0.5}MA_{0.5}Sn_{0.5}Pb_{0.5}I_3$. This paragraph needs more details as the phase dynamics and PL characteristics are more complex than the ones from the previous example.

The ToF-SIMS section is hard to follow. I would like to ask the the authors to clarify the observations and outcomes a bit better and also to link the outcomes to the previous and following sections better.

On page 14 the authors discuss the processes in films first aged in air and subsequently subjected to irradiation. This section seems to be misplaced as it makes the reader believe it is linked to the ToF-SIMS investigation. The two discussions are also merged in Figure 4, which makes it hard to follow the discussion. I would like to ask the authors to please rearrange these discussions (e.g., move to the very beginning) and to make connections between previous and following discussions a bit clearer.

On page 17 the authors write “The wavelength of light with energy higher than the band gap does not significantly affect phase reconstruction (Supplementary Fig. 17a, b).” This comment (and the respective explanations) are required earlier in the manuscript (e.g., at the very beginning when the light source is discussed).

In the end of their work, the authors mention that the decomposition processes cannot be prevented due to their fundamental nature. However, the authors have not commented on the removal of oxygen and the decomposition processes in absence of oxygen. Can the authors show a delayed decomposition if the films are encapsulated? Can the authors add an oxygen absorber layer for example that prevents oxygen from reaching the absorber? It would be helpful if the authors could show that oxygen is indeed the driving force for the decomposition and film stability improved in absence of oxygen.

Reviewer #3

(Remarks to the Author)
The authors report the phase reconstruction process of tin-lead perovskites under light irradiation in ambient air, proposing that this occurs through superoxide formation, where oxygen reacts with photoexcited electrons, leading to Pb-rich regions on the surface. However, the main conclusion of the paper lacks novelty, and the presented results do not fully validate their claims. Based on these observations, I recommend that this manuscript is not suitable for publication in Nature Communications.
Comments:
1. Recent literature reports oxidized species forming on perovskite surfaces due to air exposure. How do the authors distinguish between phase reconstruction caused by light irradiation versus air exposure? Moreover, the authors claim phase reconstruction is unaffected by moisture, despite moisture accelerating tin oxidation. How do they support this conclusion?
2. Tin-lead perovskites typically exhibit Sn-rich surfaces due to tin oxidation, yet the authors report Pb-rich regions. Could they explain this contradiction in light of other literature? (for instances, https://doi.org/10.1038/s41560-024-01613-8).
3. The manuscript does not confirm whether the atomic ratio of tin and lead matches the stoichiometry prior to irradiation. How do the authors conclude that Pb-rich regions result specifically from light exposure?
4. There are inconsistencies between the photoluminescence (PL) peak, absorbance edge, and EQE edge in determining the bandgap. Could the authors clarify this discrepancy?
5. In Fig. 1b, after 20 minutes of light irradiation, the single-junction narrow bandgap device showed an efficiency close to 0%, while the tandem device maintained 33% of its initial efficiency. If the narrow bandgap perovskite is completely oxidized and reconstructed, the tandem device may show a similar behavior. A detailed explanation is needed with the light irradiation and reconstruction process. Moreover, the manuscript reports near-zero power conversion efficiency after 20

minutes of light irradiation/air aging, yet EQE values are higher than those of fresh devices. How do the authors reconcile this?
6. The O1s core-level spectra are not included from the surface chemical analysis, and Sn and Pb peak area ratios are not confirmed across all stages. Could the authors address this? Moreover, In Fig. 2, it is concluded that the PL peak near 790 nm (stage Ⅲ) was originated from $FAPbI3$ formed on the surface. However, the XPS result in Fig. 3 shows that $Sn4+$ increases on the surface at stage Ⅲ, which is not consistent with the conclusion in Fig. 2. This indicates that the PL peak near 790 nm is likely to be a Sn-based or Sn-Pb mixed perovskite. Therefore, it is required to match the XRD and PL reference data of various Sn-based and Sn-Pb mixed perovskite compositions as mentioned in the text and Supplementray Fig. 8-10.
7. I suggest authors include detailed conditions for the stability tests, including moisture levels, incident light power, oxygen levels, and temperature?
8. TOF-SIMS analysis is provided only for the stage 3 film. Could the authors include results for stages 1 and 2 for clearer comparison and better understanding?
9. The authors suggest that Sn^{2+} vacancies lead to triiodide ion formation, creating Pb-rich regions. However, triiodide formation is typically linked to halide mobility and redox processes involving I^{-} and I_2, not tin vacancies. Could the authors provide additional clarity on this concept? Also, [Page 12, Line number 225] how do the authors conclude that Sn^{2+} oxidation drives triiodide formation based on XRD data? Moreover, the authors suggest that molecular iodine escapes to the surface to form triiodide, but supporting evidence is lacking. Could they provide more data or references?

Version 1:

Reviewer comments:

Reviewer #1

(Remarks to the Author)
The authors have addressed the comments and issues. I am satisfied with their revision. I would recommend acceptance of this manuscript.

Reviewer #2

(Remarks to the Author)
I thank the authors for their detailed revision and clarifications. The manuscript can be published in its current form.

Reviewer #3

(Remarks to the Author)
The referees' comments and questions have been thoroughly addressed with additional experiments and discussions. I have one minor issue remaining: FA_2SnI_6 is classified as a type of perovskite structure, specifically a vacancy-ordered double perovskite. Therefore, please revise the statement, 'it readily transforms into the FA_2SnI_6 non-perovskite structure. Unlike the perovskite structure, the $[SnI_6]^{4-}$ units in the FA_2SnI_6 structure do not connect through the iodide ions, but form a face-centered cubic arrangement with the A-site ions occupying the interstitials,' to accurately reflect this classification.

Open Access This Peer Review File is licensed under a Creative Commons Attribution 4.0 International License, which permits use, sharing, adaptation, distribution and reproduction in any medium or format, as long as you give appropriate credit to the original author(s) and the source, provide a link to the Creative Commons license, and indicate if changes were made.
In cases where reviewers are anonymous, credit should be given to 'Anonymous Referee' and the source.
The images or other third party material in this Peer Review File are included in the article's Creative Commons license, unless indicated otherwise in a credit line to the material. If material is not included in the article's Creative Commons license and your intended use is not permitted by statutory regulation or exceeds the permitted use, you will need to obtain permission directly from the copyright holder.
To view a copy of this license, visit https://creativecommons.org/licenses/by/4.0/

Revisions and list of changes

----

1. We sincerely thank the referee for raising this query. Upon reviewing the PL spectra of the original films, we identified some errors, which have now been corrected with the updated version.
2. The supporting information has been expanded with additional data to further substantiate the findings and discussions presented in the manuscript.
3. We appreciate the referee's careful review of the main text. In response, we have supplemented detailed environmental information on the aging process in the manuscript.
4. Thank We are grateful for the referee's insightful query. As a result, we have included new data on the irradiation aging of specific perovskite thin films in the supporting information for further clarity.

Reviewer: 1

Recommendation: Publish after minor issues/comments.

Comments:

This manuscript unveils a critical degradation mechanism of Sn-Pb perovskite under irradiation/air exposure. The proposed phase reconstruction process of Sn-Pb perovskite has been supported by full explanations and various characterizations. The superoxide generated from irradiation/air exposure drives the oxidation of Sn^{2+} *to form Sn vacancies, causing the increase of Pb-rich regions to facilitate the random generation of black-phase perovskite from yellow-phase perovskite. This work is of great interest and significance to the community of this field, which provides a new perspective to improve the long-term stability of Sn-Pb PSCs.*

We sincerely thank the referee for raising the important comments and suggestions, which have been invaluable in helping us refine and enhance the interpretation of this work.

1. One of the enlightenments from this work is somewhat confusing. For Sn-Pb perovskites, even though an iodide-rich environment can certainly reduce the formation of iodide vacancies, it simultaneously increases the amounts of tin vacancies which are deemed as the main reason impairing the performance of Sn-Pb PSCs. Thus, the strategy of exploring an iodide-rich environment to prevent iodide vacancies for Sn-Pb perovskites seems inappropriate. Please make corresponding modifications or provide explanations.

We are in full agreement that while the iodide-rich treatment applied directly to the perovskite film may somewhat reduce iodide vacancies, it simultaneously introduces a significant number of tin vacancies. In tin-only perovskites, tin vacancies generally form shallow defect states, but when tin is partially substituted with lead, the valence band is lowered due to enhanced spin-orbit coupling, potentially converting these vacancies into deep trap states. This could result in trap-mediated recombination in the absence of substantial self-doping (J. Phys. Chem. Lett. 2020, 11, 3546). However, it has been proposed that tin-vacancy formation diminishes as a larger fraction of tin is replaced with lead in mixed perovskites (ACS Energy Lett. 2021, 6, 2413; ACS Energy Lett. 2017, 2, 2159). Additionally, studies indicate that the carrier recombination rate in mixed tin-lead perovskites aged in air is higher than in pure tin-based perovskites, suggesting that deep-trap formation in intermediate tin-lead compositions may be more likely caused by iodide vacancies, which are known to generate deep-level defects in lead iodide perovskites (Adv Energy Mater. 2023, 13, 2200847; Energy Environ. Sci. 2018, 11, 702). Furthermore, the manuscript reveals that ageing progresses gradually from the surface to the deeper layers of the perovskite film. Therefore, we recommend further investigation into innovative fabrication methods for mixed lead-tin perovskites, particularly exploring iodide-rich conditions to mitigate iodide vacancies. This approach could

involve chemically anchoring the iodide vacancies either at the interface or within the bulk phase of the perovskite, rather than alter the perovskite's stoichiometric ratio. In the original text, we will ensure that the strategy for addressing iodine vacancies is articulated more clearly to avoid ambiguity.

2. Is the phase reconstruction process correlated with the light wavelength emitted by the excitation illumination?

The light-induced degradation of perovskite films primarily arises from the activation of conduction band electrons by incident light, which subsequently leads to the formation of superoxide ions and accelerates the degradation process. To investigate this, we used two wavelengths—532 nm and 633 nm—as the aging light source. As shown in Fig. R1, irradiation with both wavelengths induced phase reconstruction in the thin film, with no significant difference in the reconstruction timescale. However, when the mercury lamp' s light intensity was halved, the degradation rate of the film also decreased, suggesting that light intensity is a critical factor in determining the degradation rate.

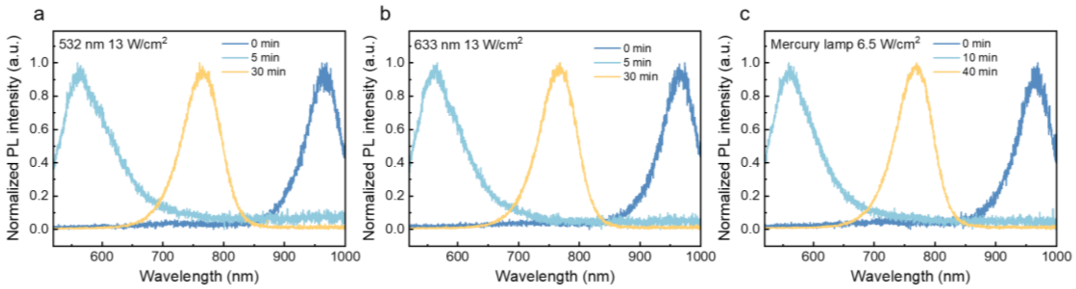

Fig. R1. The effect of light of different wavelengths and powers on the photostability of $FA_{0.7}MA_{0.3}Sn_{0.5}Pb_{0.5}I_3$ perovskite. (a) Normalized PL spectra of irradiated films under 532 nm light (532 nm light passes through a 532 nm bandpass filter). (b) Normalized PL spectra of irradiated films under 633 nm light (633 nm light passes through a 633 nm bandpass filter). (c) Normalized PL spectra of irradiated films under a Mercury lamp (Mercury lamp, 6.5 W cm^{-2}).

3. The authors claim that Sn-Pb perovskite solar cells undergo phase reconstruction during irradiation in Fig. 1c. However, the shift in phase reconstruction after an irradiation duration of more than 40 min is abrupt. It is suggested to characterize devices with irradiation durations between 40 and 60 min. This could offer better understanding into the reconstruction process.

As illustrated in Fig. R2, the tin-lead hybrid perovskite device with a thin film experiences a sudden shift in its EQE and absorption spectrum after irradiation by a mercury lamp. This phenomenon arises from the gradual phase reconstruction progressing from the surface to the bottom of the film. Initially, only parts of the surface develop a Pb-rich phase, while the bottom layer remains a mixed Sn-Pb phase, resulting in minimal change in the band gap during EQE and absorption measurements.

Only when phase reconstruction completes, creating extensive Pb-rich regions at the film’s bottom, is a marked band gap shift observed in the modified test.

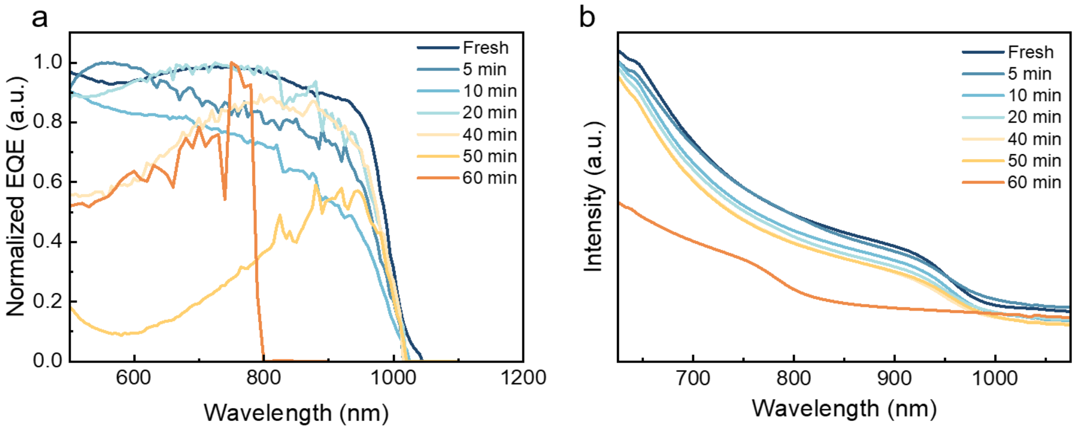

Fig. R2. Narrow bandgap perovskite single-junction PSCs. (a) External quantum efficiency spectra of a single-junction narrow bandgap perovskite solar cell under irradiation/air exposure. (b) Absorption spectra of a narrow bandgap perovskite thin film under irradiation/air exposure.

4. The migration and aggregation of Sn elements (or Sn vacancies) proposed by the authors seem to have weak reliability based solely on SIMS results. The authors should supplement characterizations and explanations on this.

As you rightly noted, analyzing TOF-SIMS profiles from a single stage is insufficient to substantiate a comparative statement. To minimize instrument-related discrepancies, we have recompiled the TOF-SIMS profiles for films across all three stages. As displayed in Fig. R3, the ion distribution on the surface of the stage I film is uniform, while films from stages II and III exhibit Pb^+ aggregation. In these Pb^+-enriched regions, clustering of FA^+ and I^- ions is also observed, along with a reduction in Sn^+ concentration. This relatively low Sn^+ intensity may result from the TOF-SIMS ion source’s limited efficiency in bombarding and releasing Sn^+ ions compared to others, rather than indicating an intrinsically low Sn^+ concentration. Although TOF-SIMS cannot determine the ions' valence states, trace amounts of SnO^+ are detectable in Pb^+-enriched regions, providing further insights into the superoxidation process.

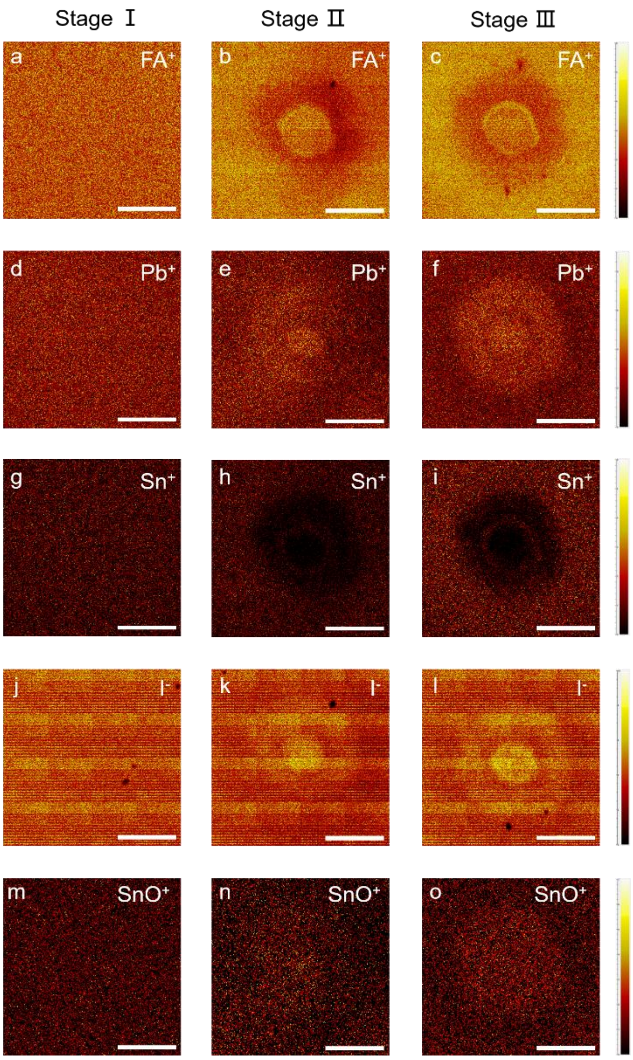

Fig. R3. The surfaced ions distribution of $FA_{0.7}MA_{0.3}Sn_{0.5}Pb_{0.5}I_3$ perovskite thin films in different stage (The scale bar is 100 μm). (a-c) TOF-SIMS mapping signals of FA^+ on the mixed $FA_{0.7}MA_{0.3}Sn_{0.5}Pb_{0.5}I_3$ perovskite films in different stages. (d-f) TOF-SIMS mapping signals of Pb^+ on the mixed $FA_{0.7}MA_{0.3}Sn_{0.5}Pb_{0.5}I_3$ perovskite films in different stages. (g-i) TOF-SIMS mapping signals of Sn^+ on the mixed $FA_{0.7}MA_{0.3}Sn_{0.5}Pb_{0.5}I_{33}$ perovskite films in different stages. (j-l) TOF-SIMS mapping signals of I^- on the mixed $FA_{0.7}MA_{0.3}Sn_{0.5}Pb_{0.5}I_3$ perovskite films in different stages. (m-o) TOF-SIMS mapping signals of SnO^+ on the mixed $FA_{0.7}MA_{0.3}Sn_{0.5}Pb_{0.5}I_3$ perovskite films in different stages.

5. Figs. 1d and 2a do not have the identical time scales for Sn-Pb perovskite phase reconstruction process. Please provide a detailed description about this.

We appreciate the reviewer's insight into this potentially confounding factor. The in-situ PL system used in our study, illustrated in Fig. R4, employs a mercury lamp as both the aging light source and the excitation source for the PL spectrum. In this configuration, the mercury lamp is focused through a microscope to produce a 100 μm spot (aging region). However, for XRD characterization of thin

films and EQE measurements of devices, a 100 µm spot is insufficient. Consequently, the mercury lamp must illuminate the film without focusing, resulting in a non-uniform light intensity distribution across the area and causing variations in the aging timeline. This aspect will be discussed in greater detail in the manuscript.

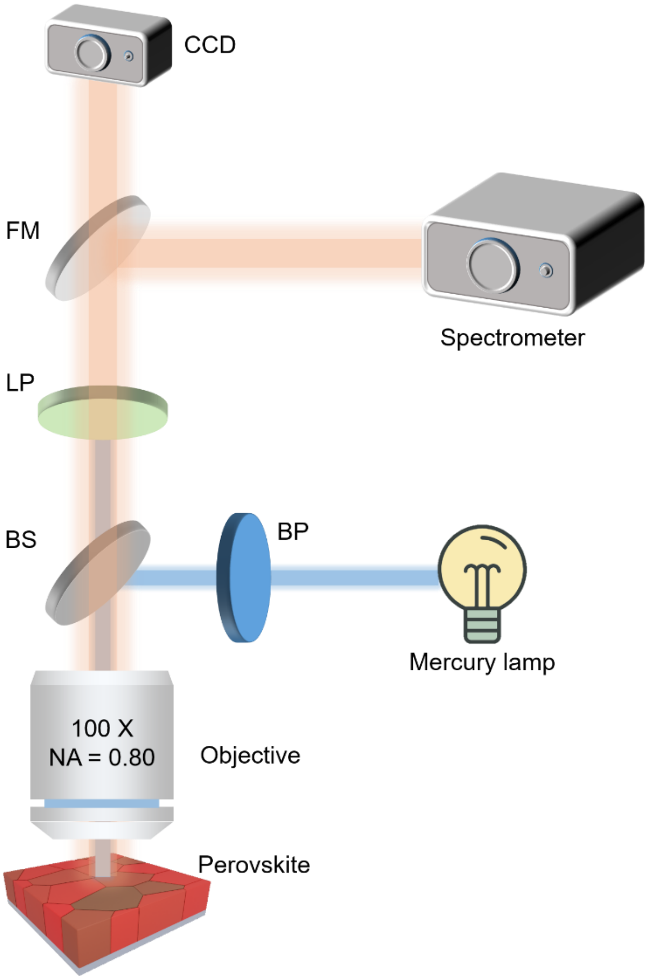

Fig. R4. Schematic of the PL microscopy experiment setup. BP: bandpass filter; BS: beam splitter; LP: long pass filter; FM: flip mirror; CCD: charge coupled device.

6. The authors selected two typical Sn-Pb perovskite compounds for investigation: $FA_{0.7}MA_{0.3}Sn_{0.5}Pb_{0.5}I_3$ and MA-free $FASn_{0.5}Pb_{0.5}I_3$, and they demonstrated that the superoxide degradation mechanism of Sn-Pb perovskites is different from their pure-Pb and pure-Sn counterparts. Furthermore, I am curious about whether this degradation mechanism of Sn-Pb perovskites varies with the ratio of Sn to Pb?

The original manuscript lacks an investigation of perovskite thin films with varying tin-lead atom ratios, which could help validate the main concepts of our study. As shown in Fig. R5, the irradiation dynamics in perovskite thin films with different tin-lead ratios are also noteworthy. None of the raw PL emission spectra of films with different Sn-Pb fractions exhibit spurious peaks beyond the primary emission peaks. For films with different tin-lead ratios, the overall trend in the PL spectra after sufficiently long irradiation indicates a shift toward the 790 nm PL emission peak. This

suggests that, despite varying tin-lead ratios, perovskite thin films undergo similar phase reconstruction processes under irradiation.

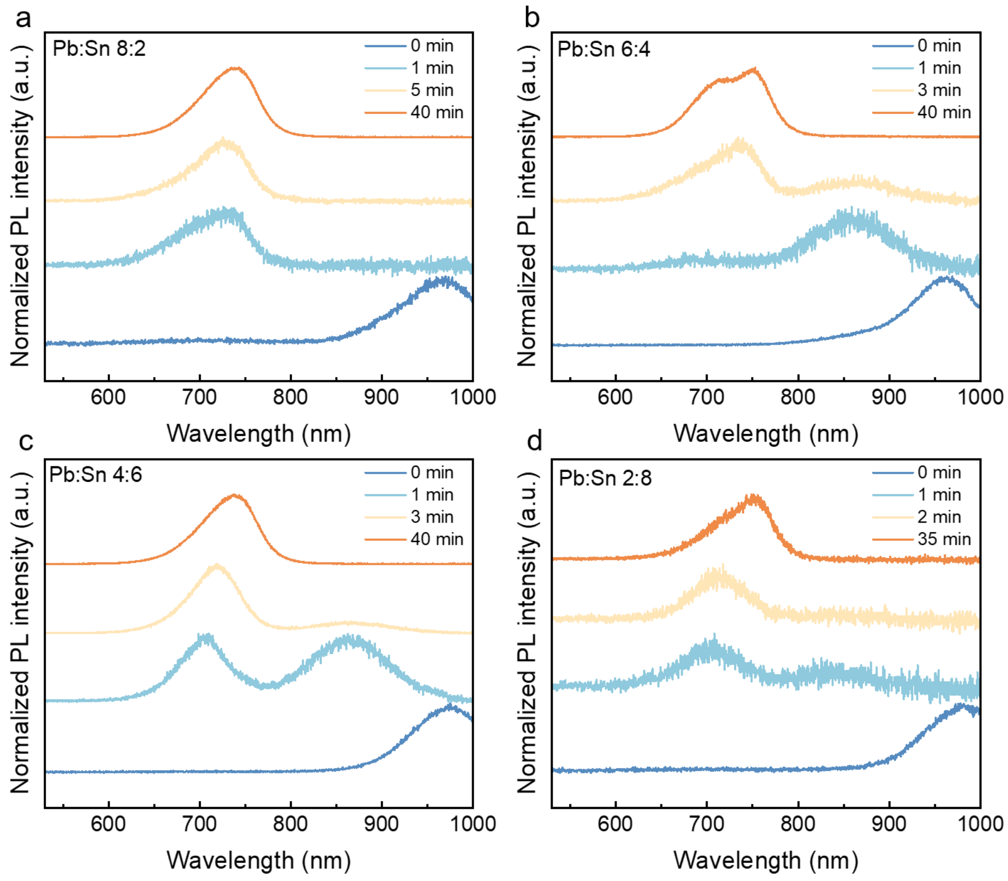

Fig. R5. PL spectra of $FA_{0.7}MA_{0.3}Sn_xPb_{1-x}I_3$ perovskite thin films under irradiation. (a) The PL spectra of $FA_{0.7}MA_{0.3}Sn_{0.2}Pb_{0.8}I_3$ perovskite films under irradiation (Mercury lamp, 13 W cm^{-2}). (b) The PL spectra of $FA_{0.7}MA_{0.3}Sn_{0.4}Pb_{0.6}I_3$ perovskite films under irradiation (Mercury lamp, 13 W cm^{-2}). (c) The PL spectra of $FA_{0.7}MA_{0.3}Sn_{0.6}Pb_{0.4}I_3$ perovskite films under irradiation (Mercury lamp, 13 W cm^{-2}). (d) The PL spectra of $FA_{0.7}MA_{0.3}Sn_{0.8}Pb_{0.2}I_3$ perovskite films under irradiation (Mercury lamp, 13 W cm^{-2}).

7. The chemistry of the surface ions of the films at three different reconstruction stages is shown in Figure 3. Please provide complete XPS mapping for a full comparison of the results.

The complete XPS spectra provide a more comprehensive visualization of the data (Fig. R6,7). Additionally, we have included XPS analyses of thermally aged films to allow for a more detailed comparison of atomic occupancies during the light-induced superoxidation process, taking into account both the light exposure duration and the temperature conditions under the mercury lamp. Comparing irradiated films with heat-aged films highlights the specific degradation mechanisms associated with photo-induced degradation. The increase in triiodide ion content in the irradiated films is shown to be significantly higher than that in thermally degraded films, as revealed by the full XPS spectrum.

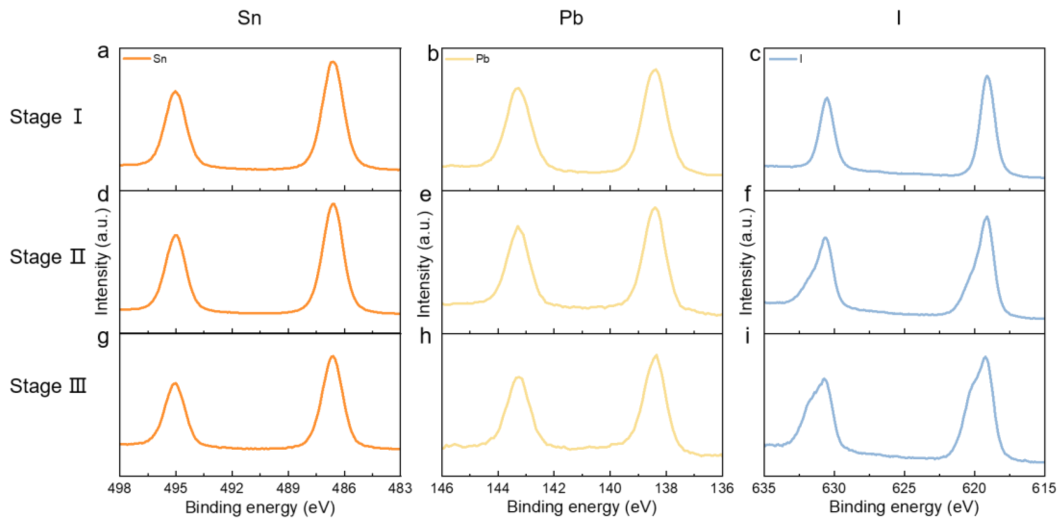

Fig. R6. The surface element properties of mixed tin-lead perovskite films during phase reconstruction. (a-c) Complete XPS spectra of mixed $FA_{0.7}MA_{0.3}Sn_{0.5}Pb_{0.5}I_3$ perovskite films in stage I: (a) Sn $3d_{5/2}$; (b) Pb $4f_{7/2}$; (c) I $3d_{5/2}$. (d-f) Complete XPS spectra of mixed $FA_{0.7}MA_{0.3}Sn_{0.5}Pb_{0.5}I_3$ perovskite films in stage II: (d) Sn $3d_{5/2}$; (e) Pb $4f_{7/2}$; (f) I $3d_{5/2}$. (g-i) Complete XPS spectra of mixed $FA_{0.7}MA_{0.3}Sn_{0.5}Pb_{0.5}I_3$ perovskite films in stage III: (g) Sn $3d_{5/2}$; (h) Pb $4f_{7/2}$; (i) I $3d_{5/2}$.

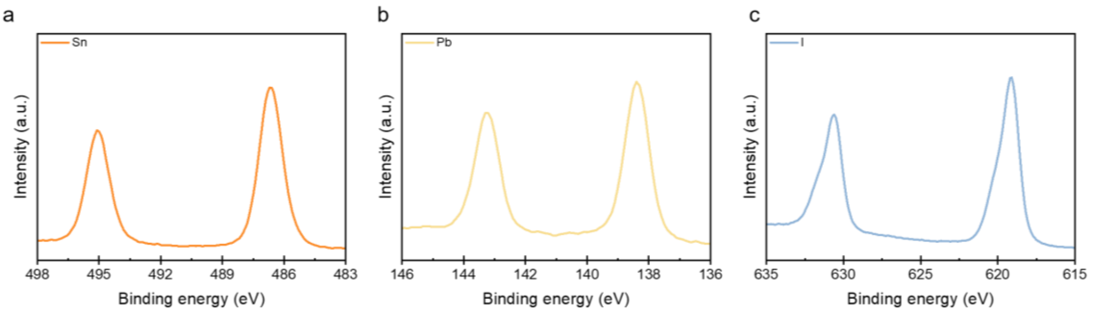

Fig. R7. Surface elemental properties of mixed tin-lead cladding crystalline films after 30 min of heating at 40 °C. (a-c) Complete XPS spectra of mixed $FA_{0.7}MA_{0.3}Sn_{0.5}Pb_{0.5}I_3$ perovskite films after 30 min of heating at 40 °C: (a) Sn $3d_{5/2}$; (b) Pb $4f_{7/2}$; (c) I $3d_{5/2}$.

8. Please correct the mistake writing of “I- and Sn2+ vacancies”.

We appreciate the reviewer’s attention to this detail. The statement regarding ionic vacancy migration indeed required clarification to prevent potential misinterpretation. We have revised it in the manuscript as follows:

"Due to the significantly slower mobility of Sn ions (with a typical timescale of hours) compared to halide ions (timescale of minutes), and in conjunction with the near-hourly reconstruction observed in Stage III, we conclude that the black phase of $FAPbI_3$ arises from the migration and accumulation of Sn ions. The degradation of FA_2SnI_6 or the formation of Sn^{4+} under prolonged irradiation results

in numerous Sn vacancies within the thin films. The migration and clustering of these ions contribute to the development of Pb-rich regions, while the original tin-lead lattice remains structurally intact. Consequently, Pb-rich regions emerge in the black phase $FAPbI_3$."

(please refer to this on Page 17 in our revised manuscript).

Reviewer: 2

Recommendation: Publish after major revisions noted.

Comments:

In their work, the authors are addressing a crucial topic of low-bandgap perovskite solar cells by investigating the phase transition processes in mixed Pb-Sn perovskites under air and light exposure. By doing so, the authors find fundamental limitations of that material class, generate new insights as well as learnings for future improvement of the operational stability of this interesting material class. The initial and final sections of the manuscript are written very well. However, some sections in between are bit hard to follow as their connections to the previous and following discussions are not fully clear. I would like to ask the authors to please work on clarifying the connections between the different investigations a bit better before the manuscript can be accepted. In addition, clarification is required on the following aspects.

We sincerely thank the reviewer for their insightful comments and valuable suggestions, which have been instrumental in enhancing our interpretation of this work.

These are major issues:

1.On page 7 the authors write "However, the edge shifting to 800 nm indicates the formation of a new perovskite phase during the aging process." It is not clear from these simple measurements that a "perovskite" phase is forming. It is also not clear that only "one" phase is forming. The authors should either proof their statements here or reword the sentence.

Thank you for highlighting this point. While our subsequent PL and XRD results presented in Figure 2 support our conclusion, we agree that the EQE shift alone does not provide conclusive evidence for the formation of a single new phase. Consequently, we have revised the sentence as follows:

"However, the shift to 790 nm indicates that the perovskite compounds underwent changes during the aging process."
(please see Page 6 in the revised manuscript).

2.On page 8 the authors indicate that the illumination with AM1.5 spectrum and mercury spectrum result in similar effects and conclude that the blue light drives the degradation. Can the authors please further check that by using different filters that remove more or less parts within the wavelength range around 475 nm and investigating the degradation mechanism of the UV filtered AM1.5 spectrum that the degradation is indeed changing (i.e., is the degradation less pronounced

without UV)?

The light-induced degradation of perovskite films primarily arises from the activation of conduction band electrons by incident light, which subsequently leads to the formation of superoxide ions, accelerating the degradation process. To investigate this, we used two wavelengths—532 nm and 633 nm—as the aging light source. As shown in Fig. R9, irradiation with both wavelengths induced phase reconstruction in the thin film, with no significant difference in the reconstruction timescale. When the power of the incident light from the mercury lamp was reduced by half, the degradation rate of the film also slowed down. This suggests that the intensity of light plays a key role in determining the rate of degradation.

To enhance clarity in the manuscript, we have revised this section as follows:

“Additionally, the PL spectra recorded at different light wavelengths and intensities revealed diverse timescales, indicating that both wavelength and light intensity are significant factors in the reconstruction process (Supplementary Fig. 9).”
(please refer to this on Page 9 in our revised manuscript).

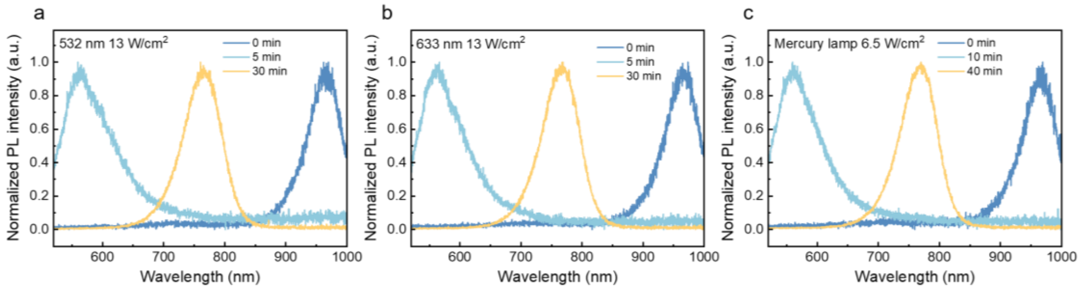

Fig. R9. The effect of light of different wavelengths and powers on the photostability of $FA_{0.7}MA_{0.3}Sn_{0.5}Pb_{0.5}I_3$ perovskite. (a) Normalized PL spectra of irradiated films under 532 nm light (532 nm light passes through a 532 nm bandpass filter). (b) Normalized PL spectra of irradiated films under 633 nm light (633 nm light passes through a 633 nm bandpass filter). (c) Normalized PL spectra of irradiated films under a Mercury lamp (Mercury lamp, 6.5 W cm^{-2}).

3.On page 8 the authors write “While one might expect the unstable tin-lead mixed perovskites to degrade under continuous irradiation, they instead transition to the black phase of $FAPbI_3$”. That sentence is misleading as the perovskite did degrade. It does not matter if the final state is again another perovskite phase, the initial phase was not stable and degraded into a more stable other phase, i.e., it degraded. Please reword.

The previous description may indeed have been misleading; this is a degradation process of the perovskite thin films rather than a simple phase transition (e.g., yellow to black) as seen in annealed

films. During degradation, various breakdown products emerge, with $FAPbI_3$ forming as one of these transformation products.

To clarify, we have revised the statement in the manuscript as follows:

"It was expected that tin-lead mixed perovskites would easily degrade into other compounds under continuous irradiation. However, surprisingly, they transition to the black phase of $FAPbI_3$, passing through an intermediate state with a PL emission at 590 nm."
(please refer to this on Page 9 in our revised manuscript).

4.In some of the initial films (e.g. Fig. 2, S2 and S3) it looks like a tiny amount of the "600 nm" phase is already present. Can the authors please comment on that observation? Where does that phase come from and is this phase responsible for the initial degradation? Can an improved phase purity improve the operational stability of the authors' devices?

As noted in the manuscript and supporting information, a minor phase with PL peak at 600 nm is present in some of the initial films. This is not due to phase instability or impurities in the perovskite films themselves, but rather stems from a slight lag in experimental testing. With the updated experimental test schematic, we observe that this real-time device utilizes a mercury lamp both as an aging and excitation light source, eliminating the need for additional excitation sources (Fig. R11). However, due to the spectrometer's detection settings, PL spectra can only be captured after tens of seconds of mercury lamp irradiation. As a result, the first PL spectrum (labeled as the 0S PL spectrum) actually reflects the film's state after several seconds of irradiation, which accounts for the initial phase. To eliminate ambiguities arising from real-time data, we have retested the PL spectra of the initial films and replaced data where inconsistencies were present (Fig. R10).

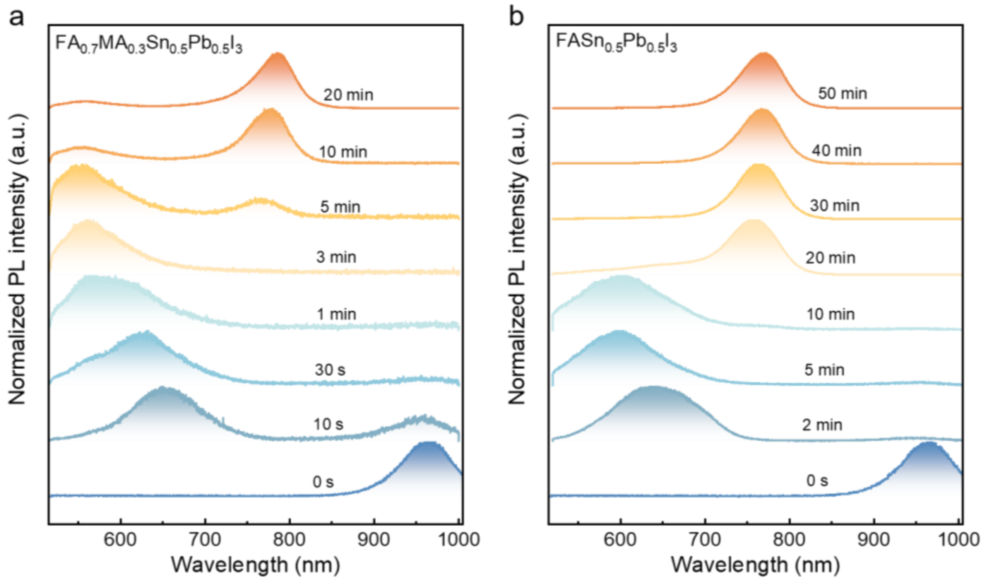

Fig. R10. In situ observation of the reconstruction of mixed tin-lead perovskite films. (a) Evolution of normalized PL spectrum results of $FA_{0.7}MA_{0.3}Sn_{0.5}Pb_{0.5}I_3$ thin films under irradiation/air

exposure (Mercury lamp, 13 W cm^{-2}). (b) Evolution of normalized PL spectrum results of $FASn_{0.5}Pb_{0.5}I_3$ thin films under irradiation/air exposure (Mercury lamp, 13 W cm^{-2}).

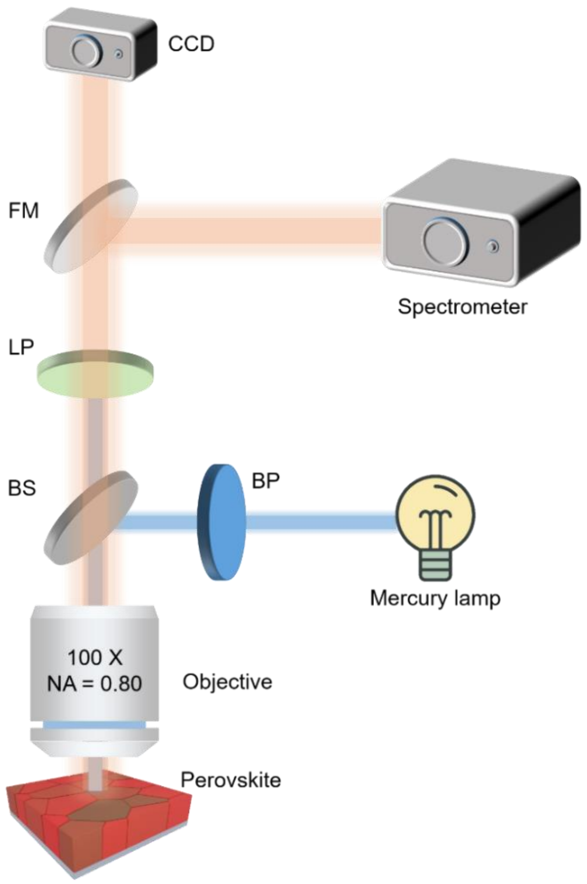

Fig. R11. Schematic of the PL microscopy experiment setup. BP: bandpass filter; BS: beam splitter; LP: long pass filter; FM: flip mirror; CCD: charge coupled device.

5.On page 10 the authors write "At Stage I, the XRD patterns confirm a pure phase of $FA_{0.7}MA_{0.3}Sn_{0.5}Pb_{0.5}I_3$", which doesn't seem correct based on my comment from above. PL shows that there is already a tiny bit of a secondary phase in the initial films. Please clarify.

We acknowledge that retaining the original data without addressing its inaccuracies may have introduced ambiguity. As noted in the manuscript and supporting information, a minor phase with PL peak at 600 nm is present in some of the initial films. This is not due to phase instability or impurities in the perovskite films themselves, but rather stems from a slight lag in experimental testing. With the updated experimental test schematic, we observe that this real-time device utilizes a mercury lamp both as an aging and excitation light source, eliminating the need for additional excitation sources (Fig. R10). However, due to the spectrometer's detection settings, PL spectra can only be captured after tens of seconds of mercury lamp irradiation. As a result, the first PL spectrum (labeled as the 0S PL spectrum) actually reflects the film's state after several seconds of irradiation, which accounts for the initial phase. Such results can indeed be misleading. To mitigate potential

experimental errors, we re-tested the PL spectra of the initial films with greater precision. This time, we used laser excitation for the initial films and extended exposure times to capture more accurate and reliable PL spectra.

6.On page 11 the authors describe the phase dynamics of $FA_{0.5}MA_{0.5}Sn_{0.5}Pb_{0.5}I_3$. This paragraph needs more details as the phase dynamics and PL characteristics are more complex than the ones from the previous example.

This section has not been reiterated in the manuscript, as the PL phase dynamics in this context are particularly intricate. To enhance clarity, we have expanded this section in the manuscript with the following addition:

"A similar phenomenon is observed in $FA_{0.5}MA_{0.5}Sn_{0.5}Pb_{0.5}I_3$ thin films, where an increase in MA content leads to a PL emission peak at 745 nm, as shown in Supplementary Fig. 11. This peak is due to Pb enrichment, resulting in the concurrent formation of $MAPbI_3$. The final state of the thin film exhibits a PL emission peak at 790 nm after 20 minutes of irradiation, suggesting that the phase reconstruction process is independent of A-site cations."
(please refer to this on Page 10 in our revised manuscript).

7.The ToF-SIMS section is hard to follow. I would like to ask the the authors to clarify the observations and outcomes a bit better and also to link the outcomes to the previous and following sections better.

The original manuscript lacked TOF-SIMS mappings for the other two stages, which limited the ability to draw meaningful comparisons from a single stage. To clarify the phase reconstruction process driven by degradation, we have now supplemented the manuscript with a complete three-phase TOF-SIMS atlas. By complementary comparison of the TOF-SIMS images of the other two stages, it can be found that the signals of FA^+, Pb^+ and I^- in stages II and III within the microzone are significantly enhanced compared to the initial film (Fig. R11). While the Sn^+ signal within this microzone is significantly reduced, this experimental phenomenon is consistent with our observation of pure Pb-based perovskite PL emission peaks on PL spectra. This addition enhances continuity with previous sections and further elaborates on the degradation process. We have added the following to the text:

"Moreover, a comparison of TOF-SIMS results across Stages I, II, and III reveals enhanced signals of FA^+, Pb^+, and I^- within the same region, while the Sn^+ signal in this area is significantly reduced (Supplementary Fig. 23)."
(please refer to this on Page 13 in our revised manuscript).

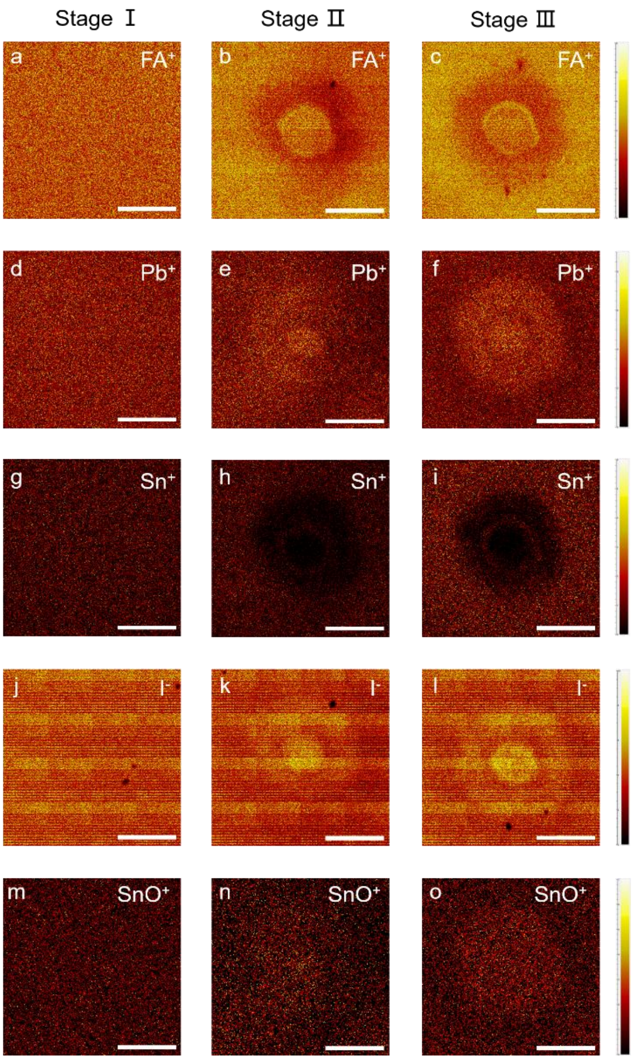

Fig. R11. The surfaced ions distribution of $FA_{0.7}MA_{0.3}Sn_{0.5}Pb_{0.5}I_3$ perovskite thin films in different stage (The scale bar is 100 μm). (a-c) TOF-SIMS mapping signals of FA^+ on the mixed $FA_{0.7}MA_{0.3}Sn_{0.5}Pb_{0.5}I_3$ perovskite films in different stages. (d-f) TOF-SIMS mapping signals of Pb^+ on the mixed $FA_{0.7}MA_{0.3}Sn_{0.5}Pb_{0.5}I_3$ perovskite films in different stages. (g-i) TOF-SIMS mapping signals of Sn^+ on the mixed $FA_{0.7}MA_{0.3}Sn_{0.5}Pb_{0.5}I_{33}$ perovskite films in different stages. (j-l) TOF-SIMS mapping signals of I^- on the mixed $FA_{0.7}MA_{0.3}Sn_{0.5}Pb_{0.5}I_3$ perovskite films in different stages. (m-o) TOF-SIMS mapping signals of SnO^+ on the mixed $FA_{0.7}MA_{0.3}Sn_{0.5}Pb_{0.5}I_3$ perovskite films in different stages.

8.On page 14 the authors discuss the processes in films first aged in air and subsequently subjected to irradiation. This section seems to be misplaced as it makes the reader believe it is linked to the ToF-SIMS investigation. The two discussions are also merged in Figure 4, which makes it hard to follow the discussion. I would like to ask the authors to please rearrange these discussions (e.g., move to the very beginning) and to make connections between previous and following discussions a bit clearer.

As noted, there was ambiguity in the layout of this section concerning the sequence of air aging followed by irradiation. The primary focus of this section is to demonstrate that superoxide degradation can occur extensively in tin-lead mixed perovskites, affecting both pristine films and already degraded films (Fig. R12). Therefore, the irradiation results of air-aged films have been included here and are connected to the final mechanism diagram. To improve clarity and prevent misinterpretation, we have revised the diagram to enhance reader comprehension.

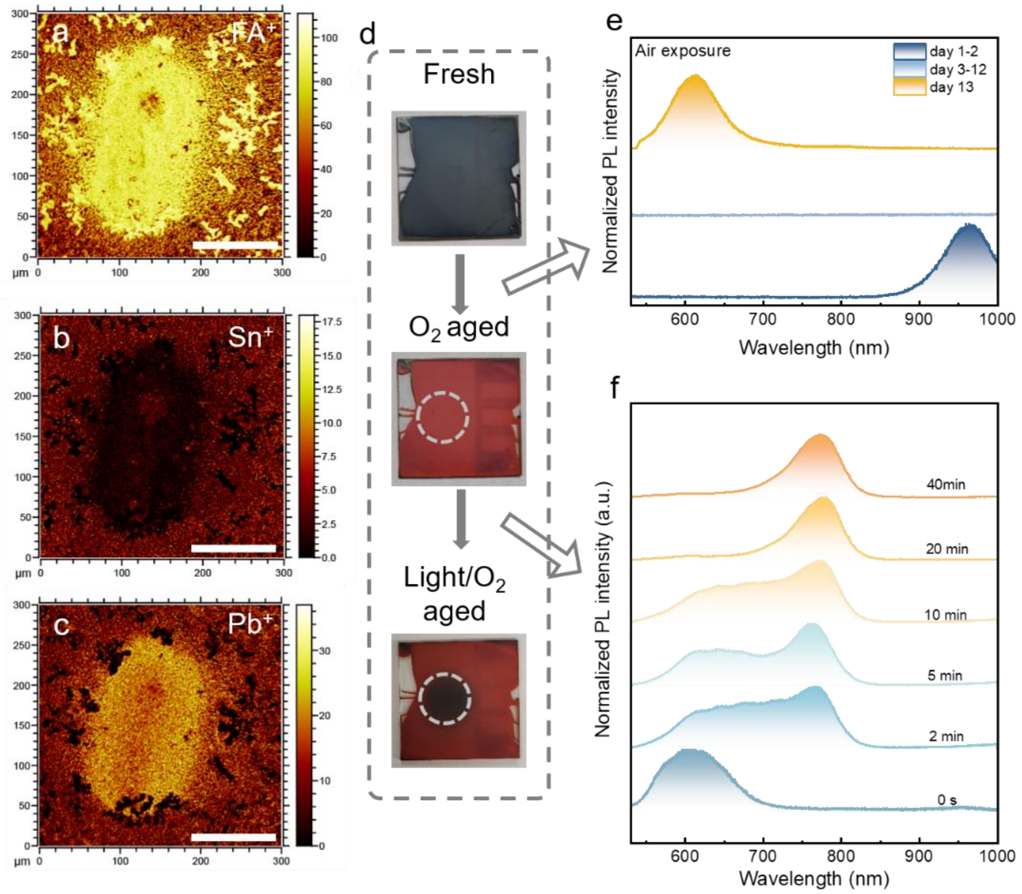

Fig. R12. The surfaced ions distribution of mixed tin-lead perovskite thin films during phase reconstruction and the phase reconstruction processes in air-aged tin-lead perovskite thin films. (a-c) TOF-SIMS mapping signals of (a) FA^+, (b) Pb^+, (c) Sn^+ on the mixed $FASn_{0.5}Pb_{0.5}I_3$ perovskite films in stage III. The scale bar is 100 μm. (d) Digital images of the fresh $FA_{0.7}MA_{0.3}Sn_{0.5}Pb_{0.5}I_3$ perovskite films, the films with air exposure, and the films with continuous blue-light irradiation after air exposure. (e) Evolution of normalized PL spectrum results of $FA_{0.7}MA_{0.3}Sn_{0.5}Pb_{0.5}I_3$ perovskite films with air exposure. (f) Evolution of normalized PL spectrum results of $FA_{0.7}MA_{0.3}Sn_{0.5}Pb_{0.5}I_3$ perovskite films with air exposure under continuous blue-light irradiation (Mercury lamp, 13 W cm^{-2}).

9.On page 17 the authors write “The wavelength of light with energy higher than the band gap does not significantly affect phase reconstruction (Supplementary Fig. 17a, b).” This comment (and the respective explanations) are required earlier in the manuscript (e.g., at the very beginning

when the light source is discussed).

The wavelength and intensity of light were previously omitted from the discussion on factors influencing degradation, though their early inclusion in the manuscript is crucial.

In our revised manuscript, we clarified this point with the following addition:

"Factors affecting the overall reconstruction process include oxygen content, light wavelength, and power (Supplementary Figs. 7-9). Thin films irradiated under varying oxygen levels exhibit differences in PL spectra timescales. Notably, the degradation rate accelerates at high oxygen content and decelerated under low oxygen conditions (Supplementary Figs. 7, 8). Additionally, PL spectra recorded at different light wavelengths and intensities reveal diverse timescales, indicating that both wavelength and light intensity are significant factors in the reconstruction process (Supplementary Fig. 9)."
(please refer to this on Page 9 in our revised manuscript).

10.In the end of their work, the authors mention that the decomposition processes cannot be prevented due to their fundamental nature. However, the authors have not commented on the removal of oxygen and the decomposition processes in absence of oxygen. Can the authors show a delayed decomposition if the films are encapsulated? Can the authors add an oxygen absorber layer for example that prevents oxygen from reaching the absorber? It would be helpful if the authors could show that oxygen is indeed the driving force for the decomposition and film stability improved in absence of oxygen.

As anticipated, the PL spectra of encapsulated films demonstrated good stability when aged under mercury lamp irradiation. Additionally, we applied an interfacial layer to insulate the film from oxygen, aiming to mitigate superoxidation. However, as shown in Fig. R13, surface modification with PMMA only slightly extended the degradation time and did not effectively inhibit the reconstruction process. To further confirm oxygen's critical role in light-induced superoxidative degradation, we used an airtight vacuum chamber to control oxygen levels (setup schematic shown in Fig. R14). The results indicate that increased oxygen content accelerated degradation, while reduced oxygen content slowed it (Fig. R15), emphasizing oxygen's essential role in driving the superoxidation reaction.

To clarify, we have revised the statement in the manuscript as follows:

"Factors affecting the overall reconstruction process include oxygen content, light wavelength, and power (Supplementary Figs. 7-9). Thin films irradiated under varying oxygen levels exhibit differences in PL spectra timescales. Notably, the degradation rate accelerates at high oxygen content and decelerated under low oxygen conditions (Supplementary Figs. 7, 8)."
(please refer to this on Page 9 in our revised manuscript).

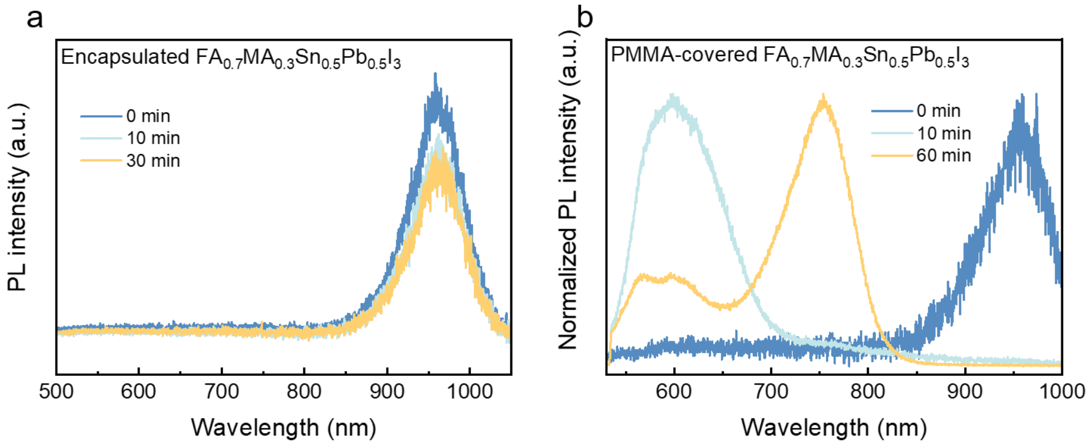

Fig. R13. (a) Evolution of normalized PL spectrum results of encapsulated $FA_{0.7}MA_{0.3}Sn_{0.5}Pb_{0.5}I_3$ thin films under irradiation (Mercury lamp, 13 W cm^{-2}). (b) Evolution of normalized PL spectrum results of PMMA-covered $FA_{0.7}MA_{0.3}Sn_{0.5}Pb_{0.5}I_3$ thin films under irradiation (Mercury lamp, 13 W cm^{-2}).

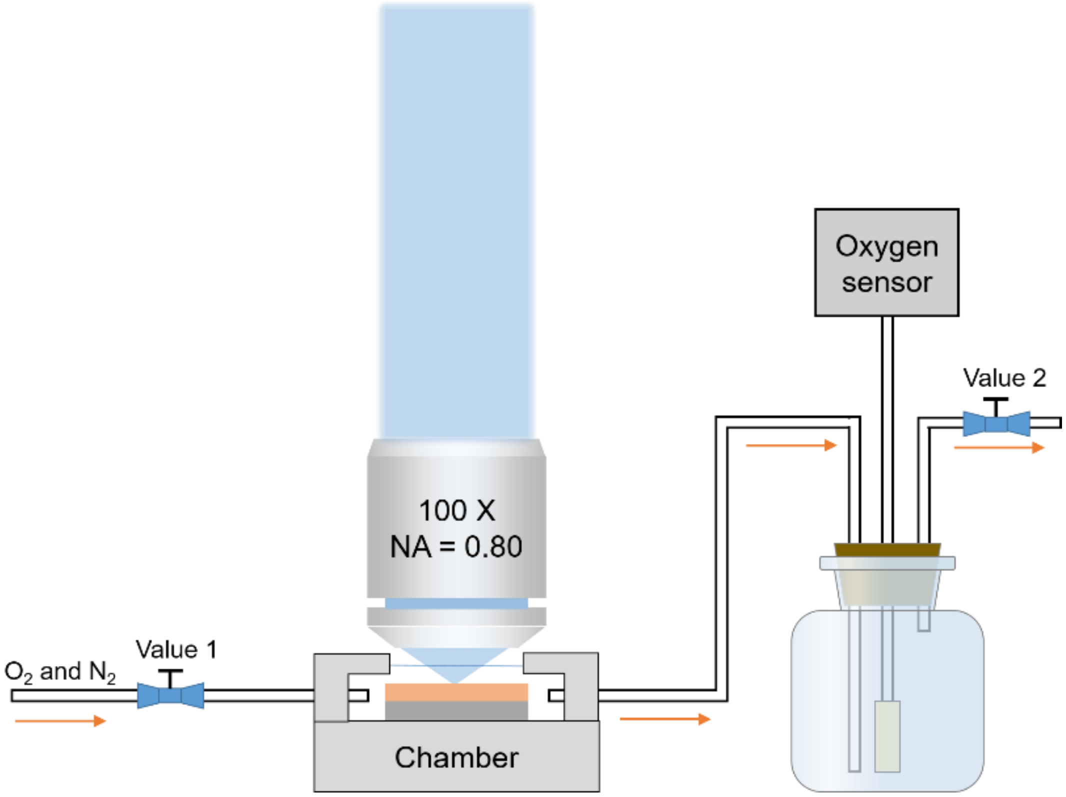

Fig. R14. Schematic diagram of the experimental setup for oxygen content control. Oxygen content is controlled by passing a mixture of gases into a closed chamber.

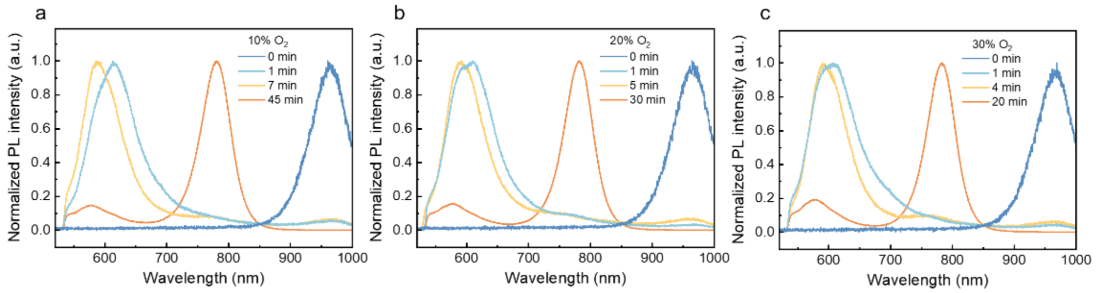

Fig. R15. Effect of oxygen content on $FA_{0.7}MA_{0.3}Sn_{0.5}Pb_{0.5}I_3$ perovskite thin films under irradiation. (a) Normalized PL spectra of $FA_{0.7}MA_{0.3}Sn_{0.5}Pb_{0.5}I_3$ perovskite films under 10% O_2. (b) Normalized PL spectra of $FA_{0.7}MA_{0.3}Sn_{0.5}Pb_{0.5}I_3$ perovskite films under 20% O_2. (b) Normalized PL spectra of $FA_{0.7}MA_{0.3}Sn_{0.5}Pb_{0.5}I_3$ perovskite films under 30% O_2

Reviewer: 3

Recommendation: Not recommended for publication.

Comments:

The authors report the phase reconstruction process of tin-lead perovskites under light irradiation in ambient air, proposing that this occurs through superoxide formation, where oxygen reacts with photoexcited electrons, leading to Pb-rich regions on the surface. However, the main conclusion of the paper lacks novelty, and the presented results do not fully validate their claims. Based on these observations, I recommend that this manuscript is not suitable for publication.

We sincerely thank the reviewer for their valuable comments and suggestions, which have greatly contributed to enhancing our interpretation of this work.

A comprehensive understanding of the degradation mechanisms in both tandem solar cells and Sn-Pb perovskites is essential for advancing their commercialization. Alloying Sn and Pb in Sn-Pb perovskites introduces a far more complex degradation process compared to single metal-ion-based perovskites. In our study, we examine the efficiency decay process of all-perovskite tandem devices, identifying that instability primarily arises from the Sn-Pb perovskite component. We then investigate the degradation process of Sn-Pb perovskites using a range of analytical techniques. This leads us to uncover an intriguing, irreversible phase reconstruction process occurring during the degradation of Sn-Pb perovskites, providing valuable insights that could inform future strategies for enhancing the stability of these materials and their tandem devices.

The main novelties of this work are highlighted as follows:

1. **A Novel Degradation Mechanism in Sn-Pb Perovskites:** Our research reveals that light irradiation and O_2 induce a superoxidation process, strongly evidenced by a range of experimental techniques such as in-situ photoluminescence, XRD, XPS, and TOF-SIMS. Unlike the common belief that moisture and high temperature are the primary degradation factors, our results highlight the formation of superoxide from light irradiation and oxygen as the primary cause of Sn-Pb perovskite degradation, offering a new perspective to the perovskite community.

2. **A Unique Phase Reconstruction Process:** We discovered that mixed Sn-Pb perovskites undergo a distinctive degradation process in three stages: $FASn_{0.5}Pb_{0.5}I_3$ first degrades to the non-perovskite phase FA_2SnI_6, then converts the yellow phase $FAPbI_3$ to the black phase. This process, characterized by the formation of Sn^{2+} vacancies, triiodide ions, and Pb-rich regions, has not been observed in other perovskites, offering a fresh perspective on material behavior.

3. **Broad Applicability for Various Mixed Sn-Pb Perovskites:** Our investigation extended to a

range of tin-lead perovskite compounds and comparisons with pure Sn- or pure Pb-based perovskites. We found that the unique degradation process involving superoxide formation is consistent across different Sn-Pb perovskites, regardless of the organic cations used. This broad applicability is highly relevant to researchers studying narrow-bandgap mixed Sn-Pb perovskite materials and other mixed metal-ion-based materials.

4. **Guidance for Enhancing Tandem Solar Cell Stability:** Our findings indicate that the superoxidation process in Sn-Pb perovskite thin films involves both Sn^{2+} oxidation and iodide oxidation, leading to triiodide ion generation. To enhance the stability of tin-lead perovskites, it is crucial to maintain an iodide-rich environment and anchor iodine ions to prevent iodide vacancies, in addition to eliminating tin inhomogeneities.

In the revised manuscript, we strengthened our arguments by adding data on the irradiation degradation process of films with different tin-lead ratios. We also included TOF-SIMS mappings for various aging stages and XPS mappings for thermally induced degraded films. These additions further substantiate our conclusions.

These are major issues:

1. Recent literature reports oxidized species forming on perovskite surfaces due to air exposure. How do the authors distinguish between phase reconstruction caused by light irradiation versus air exposure? Moreover, the authors claim phase reconstruction is unaffected by moisture, despite moisture accelerating tin oxidation. How do they support this conclusion?

Figure R16 shows the PL spectra of tin-lead perovskite films aged in air without exposure. The PL emission peaks disappear after two days. Over time, a new PL emission peak appears around 620 nm, likely corresponding to the yellow phase of perovskite formed via tin-lead degradation. This observation suggests a fundamental difference between the intrinsic mechanisms of air-induced and light-induced aging, resulting in distinct manifestations in the PL spectra throughout the aging process.

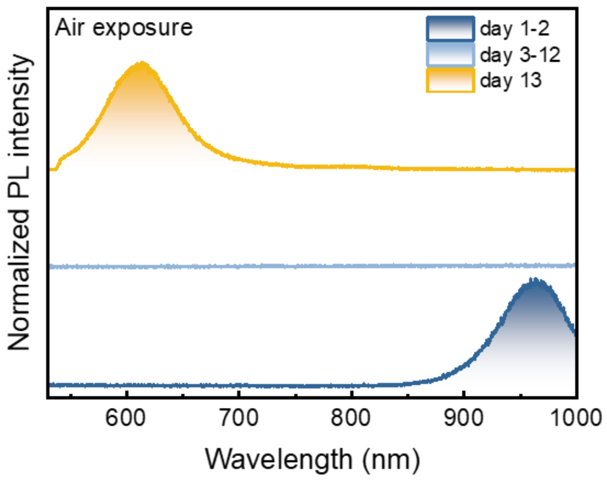

Fig. R16. Evolution of normalized PL spectrum results of $FA_{0.7}MA_{0.3}Sn_{0.5}Pb_{0.5}I_3$ perovskite films with air exposure.

Figure R17 displays the PL spectra of tin-lead perovskite films subjected to light-induced aging under varying humidity conditions, revealing that the duration of PL emission peaks at different wavelengths remains largely unchanged. Previous studies have shown that moisture accelerates tin-based perovskite degradation in air by introducing additional degradation pathways (Nat. Commun. 2021, 12, 2853). However, in that study, moisture accelerated film aging in air for up to 24 hours, indicating a more moderate impact on degradation. In contrast, the rate of light-induced aging observed here far exceeds 24 hours, with superoxidation driving a significantly more intense reaction.

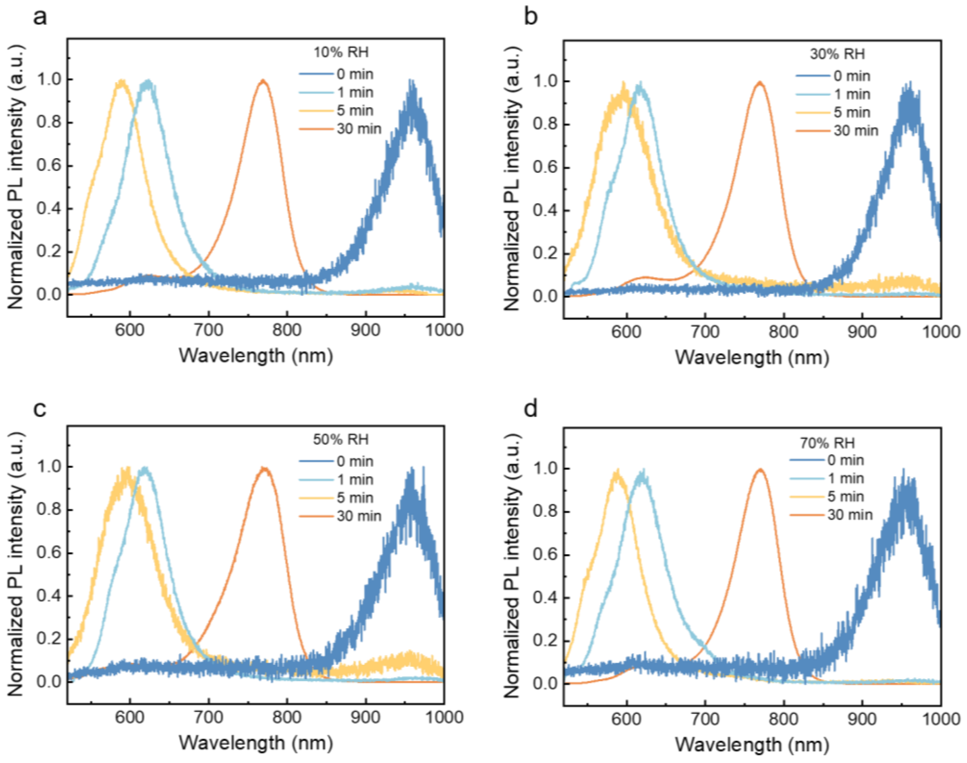

Fig. R17. Effect of moisture on $FA_{0.7}MA_{0.3}Sn_{0.5}Pb_{0.5}I_3$ perovskite thin films under irradiation. (a)

Normalized PL spectra of $FA_{0.7}MA_{0.3}Sn_{0.5}Pb_{0.5}I_3$ perovskite films under 10% RH. (b) Normalized PL spectra of $FA_{0.7}MA_{0.3}Sn_{0.5}Pb_{0.5}I_3$ perovskite films under 30% RH. (c) Normalized PL spectra of $FA_{0.7}MA_{0.3}Sn_{0.5}Pb_{0.5}I_3$ perovskite films under 50% RH. (d) Normalized PL spectra of $FA_{0.7}MA_{0.3}Sn_{0.5}Pb_{0.5}I_3$ perovskite films under 70% RH.

.

2. Tin-lead perovskites typically exhibit Sn-rich surfaces due to tin oxidation, yet the authors report Pb-rich regions. Could they explain this contradiction in light of other literature? (for instances, https://doi.org/10.1038/s41560-024-01613-8).

We agree that, after tin oxidation, the surface of tin-lead perovskite films frequently shows tin enrichment, contrasting with the Pb-rich regions reported in the manuscript. This discrepancy arises for two main reasons. First, the manuscript lacks detail in emphasizing the extent of Pb-rich regions on the surface. Second, the broad detection range of certain characterization tools (e.g., XPS) can lead to ambiguity without thorough explanations. To address this, we have added a detailed clarification in the revised manuscript. Figure R18 presents the tin-lead atom ratios on the film surface at different irradiation stages, consistently showing an increase in tin atoms on the aged film's surface. These results do not conflict with the presence of Pb-rich regions, as the XPS test area is larger than the post-focused light area and does not exclusively capture the irradiated micro-regions.

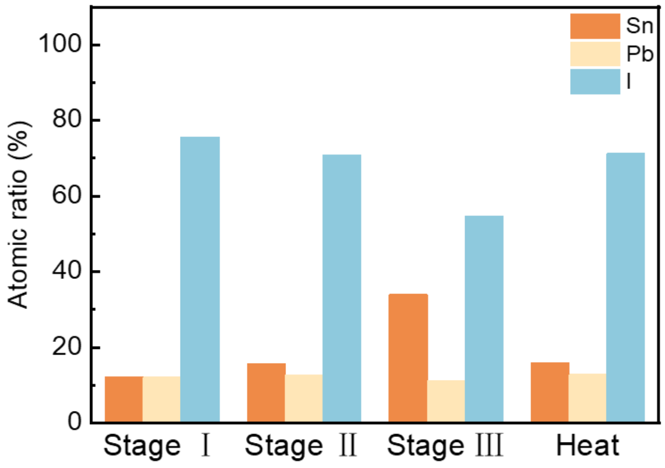

Fig. R18. Proportion of elemental content on the surface of mixed $FA_{0.7}MA_{0.3}Sn_{0.5}Pb_{0.5}I_3$ crystal films under different conditions.

3. The manuscript does not confirm whether the atomic ratio of tin and lead matches the stoichiometry prior to irradiation. How do the authors conclude that Pb-rich regions result specifically from light exposure?

We fully understand the need to strengthen this aspect of the experiment and discussion. The tin-lead atomic ratios shown in Fig. R19 were derived from XPS mapping data across the three phases,
s
u
p
p
l

emented by films aged at the same temperature and duration as the light-induced degradation

(
h
e
a
t

When comparing the Sn^{4+} fraction in Sn with the Pb^{+} to I^{-} ratio, we observe that Sn^{4+} proportions induced by heating align more closely with those induced by light exposure. Additionally, the Pb^{+} to I^{-} ratio in heat-induced samples is lower than in light-induced samples. We refrained from directly comparing Sn^{4+} to I^{-}, as the complex nature of tin oxidation products goes beyond what a single-element comparison can capture. Tin vacancies typically function as shallow defect states in tin-only perovskites; however, when tin is partially substituted with lead, the valence band is lowered due to enhanced spin-orbit coupling, which can transform these vacancies into deep trap states in mixed tin-lead perovskites. This increases trap-mediated recombination without substantial self-[illegible] in the Pb-rich regions within the microregions.

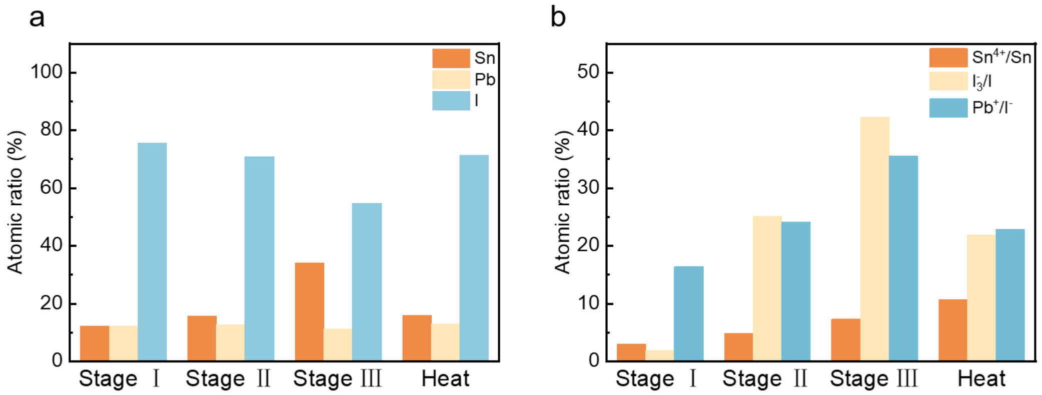

Fig. R19. (a) Proportion of elemental content on the surface of mixed $FA_{0.7}MA_{0.3}Sn_{0.5}Pb_{0.5}I_3$ crystal films under different conditions. (b) Proportion of different valence content of elements on the surface of mixed $FA_{0.7}MA_{0.3}Sn_{0.5}Pb_{0.5}I_3$ crystal films under different conditions.

4. There are inconsistencies between the photoluminescence (PL) peak, absorbance edge, and EQE edge in determining the bandgap. Could the authors clarify this discrepancy?

It is quite normal for the bandgaps determined from PL, UV-vis, and EQE spectra to show slight differences, as observed in the literature (Nat. Energy. 2023, 8, 610–620; Adv. Funct. Mater. 2023, 33, 2300473). The principle of photoluminescence (PL) spectroscopy relies on the phenomenon that when a semiconductor absorbs a photon, an electron transitions from the valence band to the

conduction band and then returns to the valence band, emitting a photon. The semiconductor's optical bandgap, which also corresponds to the visible absorption spectrum, can be calculated by measuring the wavelength of this emitted light. In contrast, EQE (external quantum efficiency) spectroscopy determines the electrical bandgap by measuring the relationship between the photogenerated current and incident photons. During EQE testing, the device temperature rises due to electron production, slightly altering the bandgap—an effect absent in PL spectroscopy. Consequently, the two bandgaps may differ slightly. Additionally, the Stokes shift explains that the energy of absorbed photons is higher than that of emitted photons in solids, causing the fluorescence spectrum to shift from the absorption spectrum. Furthermore, due to byproducts in the degradation process of perovskite films, minor differences may occur between the visible and PL spectra. Variability in the films also introduces small inconsistencies in the light degradation process. As shown in Fig. R20, after repeated testing and calculations, close agreement was achieved among all three measurements.

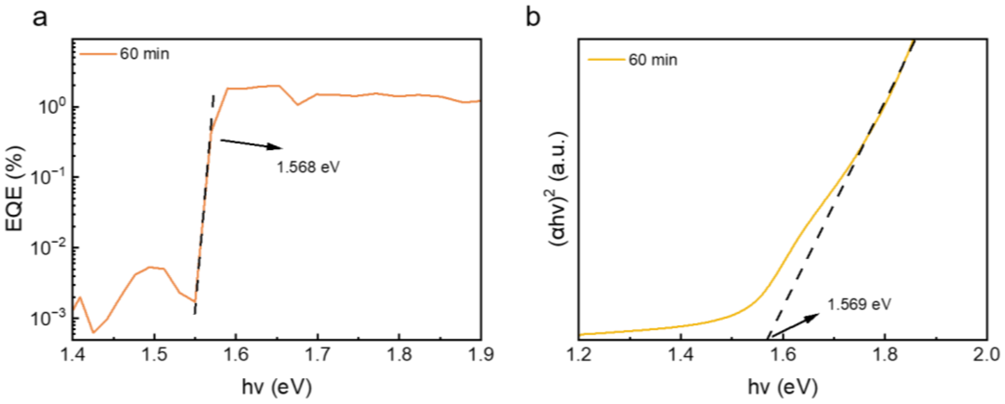

Fig R20. (a) Optical band gap of a film aged for 60 min under a mercury lamp, as calculated by EQE. (b) Optical bandgap of films aged for 60 min in a mercury lamp, calculated from absorption spectra.

5. In Fig. 1b, after 20 minutes of light irradiation, the single-junction narrow bandgap device showed an efficiency close to 0%, while the tandem device maintained 33% of its initial efficiency. If the narrow bandgap perovskite is completely oxidized and reconstructed, the tandem device may show a similar behavior. A detailed explanation is needed with the light irradiation and reconstruction process. Moreover, the manuscript reports near-zero power conversion efficiency after 20 minutes of light irradiation/air aging, yet EQE values are higher than those of fresh devices. How do the authors reconcile this?

It is widely acknowledged that all-perovskite tandem solar cells generally exhibit greater stability than single-junction narrow-bandgap mixed Sn-Pb cells, a finding supported by reports from other reputable groups (e.g., Adv. Energy Mater. 2023, 13, 2301218). Our observed stability trend between single-junction and tandem cells aligns with the literature and has been consistently

reproduced in our lab.

This stability difference primarily arises from variations in light exposure between single-junction Sn-Pb cells and tandem structures. For single-junction Sn-Pb cells, the full light spectrum is applied, leading to higher exposure. In tandems, however, the top wide-bandgap subcells filter the light spectrum, significantly reducing the light exposure for the bottom mixed Sn-Pb subcells. Additionally, the top wide-bandgap subcells benefit from protection against environmental factors provided by the bottom Sn-Pb subcells, further enhancing the tandem’s stability. This stands in contrast to silicon-perovskite tandem solar cells, where stable silicon bottom subcells result in stability comparable to that of their top single-junction wide-bandgap cells.

To clarify this point, in our revised manuscript, we have added the following sentence after the tandem stability discussion:

"It is important to note that the stability of all-perovskite tandems is generally superior to that of their single-junction mixed tin-lead perovskite counterparts. This is primarily because the light irradiation on the bottom tin-lead perovskite subcells in tandem configurations is considerably weaker, as it is filtered by the top wide-bandgap subcells."

(please refer to this on Page 6 in our revised manuscript).

The EQE data in Fig. R21 of the manuscript are normalized, which may give the impression that devices aged for longer periods exhibit higher EQE values in certain wavelength ranges than the original devices. The EQE data were normalized because prolonged irradiation significantly reduces the EQE values compared to pristine devices, making it challenging to visualize the bandgap changes in the EQE. To avoid similar ambiguities, we have included the raw, un-normalized EQE data in the supporting information.

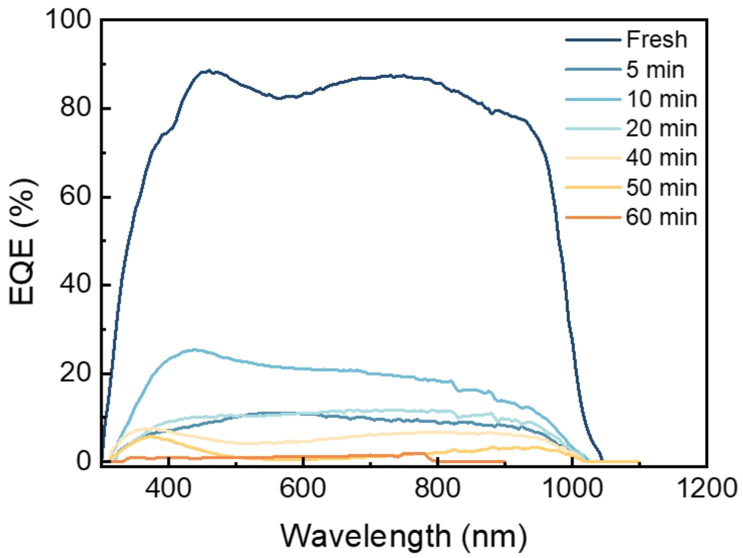

Fig R21. External quantum efficiency spectra of a single-junction narrow bandgap perovskite solar cell under irradiation/air exposure.

6. The O1s core-level spectra are not included from the surface chemical analysis, and Sn and Pb

peak area ratios are not confirmed across all stages. Could the authors address this? Moreover, In Fig. 2, it is concluded that the PL peak near 790 nm (stage Ⅲ) was originated from $FAPbI_3$ formed on the surface. However, the XPS result in Fig. 3 shows that Sn^{4+} increases on the surface at stage Ⅲ, which is not consistent with the conclusion in Fig. 2. This indicates that the PL peak near 790 nm is likely to be a Sn-based or Sn-Pb mixed perovskite. Therefore, it is required to match the XRD and PL reference data of various Sn-based and Sn-Pb mixed perovskite compositions as mentioned in the text and Supplementray Fig. 8-10.

Including this experimental data is essential. Supplementary Fig. R23 shows the XPS profiles of O on the film surface at various stages. To better isolate the effects of temperature and provide a valid comparison, we also included a control group subjected to heating aging under the same time and temperature conditions as the light exposure. The super-oxidation process on films aged with mercury lamps is significantly more intense than in heat-aged films (Fig. R22). This conclusion is supported by the substantial increase in the absolute intensity of O in XPS from Stage III, where the O peak at 531.3 eV corresponds to the binding energy of O with Sn^{4+}. The notable increase of this peak in Stage III further indicates that the light-induced oxidation process is more pronounced.
As shown in Fig. R23, the surfaces of both light-aged and heat-aged films exhibit a higher proportion of Sn relative to Pb, a phenomenon consistent with previous reports (doi.org/10.1038/s41560-024-01613-8).

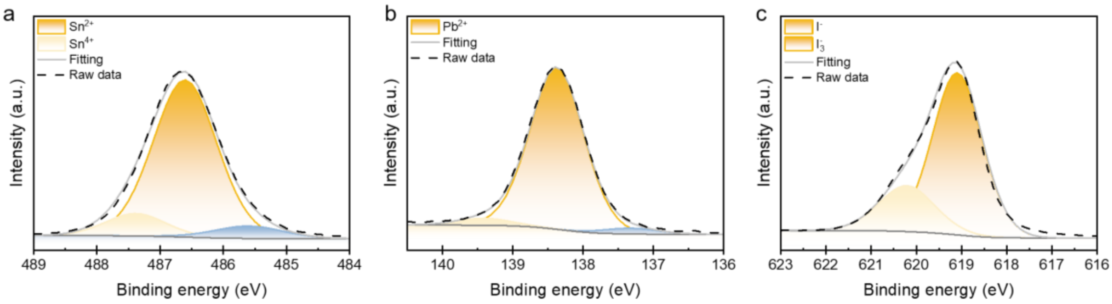

Fig. R22. Surface elemental properties of mixed tin-lead cladding crystalline films after 30 min of heating at 40 °C. (a-c) XPS spectra of mixed $FA_{0.7}MA_{0.3}Sn_{0.5}Pb_{0.5}I_3$ perovskite films after 30 min of heating at 40 °C: (a) Sn $3d_{5/2}$; (b) Pb $4f_{7/2}$; (c) I $3d_{5/2}$.

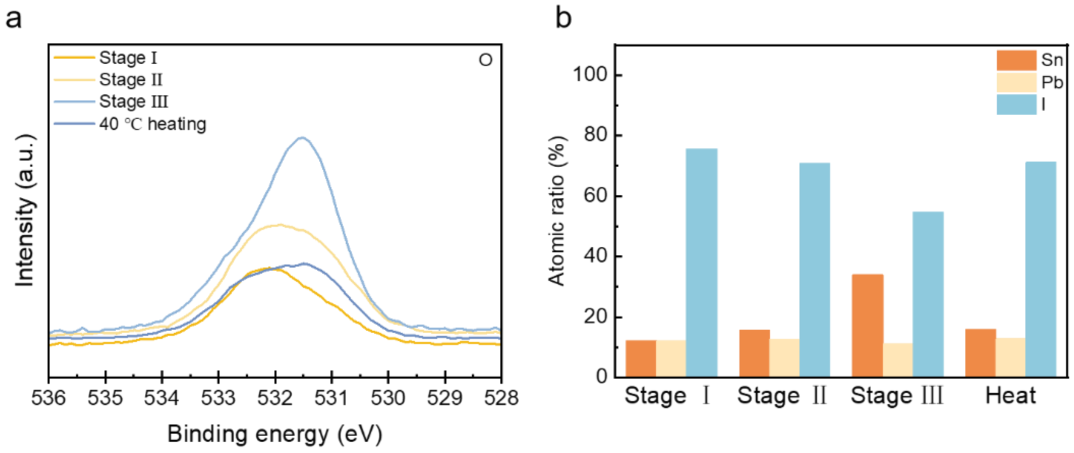

Fig. R23. Surface elemental properties of mixed tin-lead cladding crystalline films under different

conditions. (a) Complete XPS spectra of O of mixed $FA_{0.7}MA_{0.3}Sn_{0.5}Pb_{0.5}I_3$ perovskite films under different conditions. (b) Proportion of elemental content on the surface of mixed $FA_{0.7}MA_{0.3}Sn_{0.5}Pb_{0.5}I_3$ crystal films under different conditions.

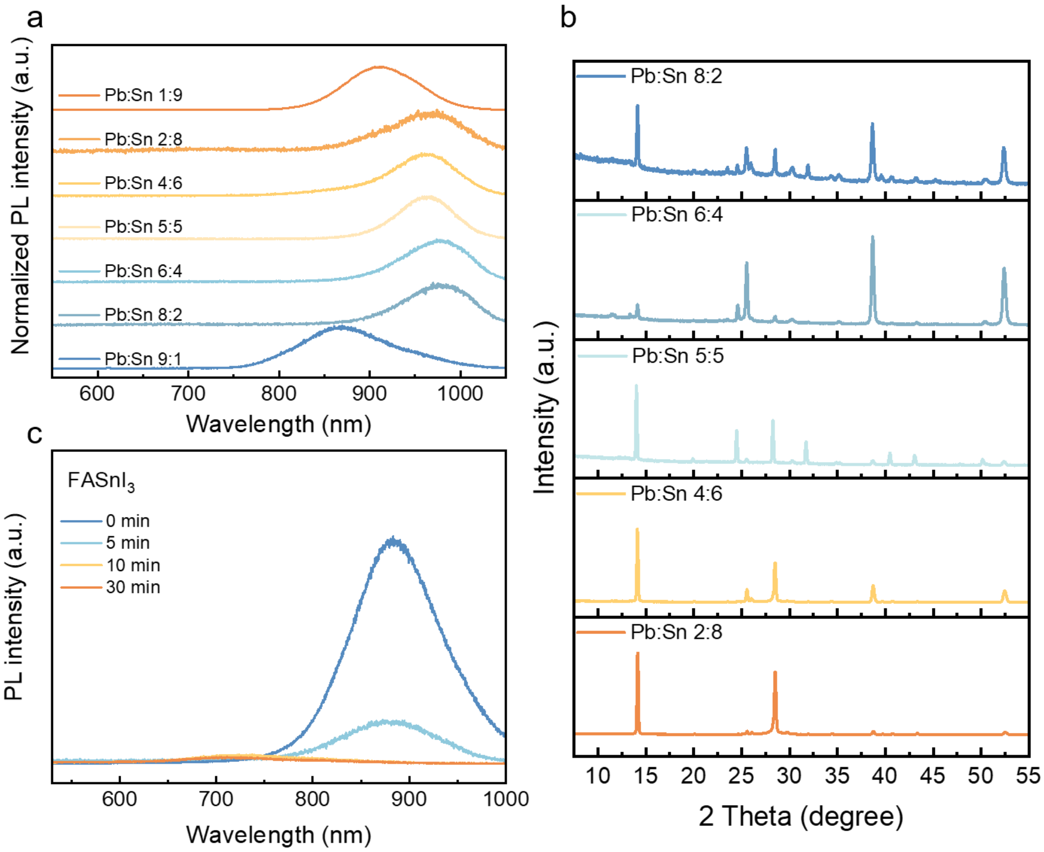

Fig. R24. The XRD and PL spectra of $FA_{0.7}MA_{0.3}Sn_xPb_{1-x}I_3$ perovskite thin films. (a) Steady-state PL spectra of $FA_{0.7}MA_{0.3}Sn_xPb_{1-x}I_3$ thin films on the cover glass. (b) XRD patterns of $FA_{0.7}MA_{0.3}Sn_xPb_{1-x}I_3$ thin films on the cover glass. (c) Evolution of normalized PL spectrum results of $FASnI_3$ thin films under light/air exposure (Mercury lamp, 13 W cm^{-2}).

Figures R24 a and b present the PL spectra and XRD patterns of perovskite films with varying tin-lead ratios. The PL emission wavelengths of the tin-lead mixed chalcogenide films are all significantly above 790 nm, indicating that the 790 nm PL emission peak is not associated with tin-lead chalcogenide phases in different ratios. Figure R24 c shows the degradation of a pure tin-based perovskite film under irradiation. The pristine pure tin perovskite film exhibits a PL emission peak well above 790 nm, and the degradation process does not show a shift of the PL emission peak toward 790 nm. These findings confirm that the 790 nm PL emission peak originates from Pb-based perovskites rather than perovskites with other compositions.

7. I suggest authors include detailed conditions for the stability tests, including moisture levels, incident light power, oxygen levels, and temperature?

Thank you for this careful suggestion. We realized that we had overlooked annotating the detailed conditions for the entire experimental procedure. To address this, we have included a discussion of the experimental conditions in the revised manuscript, with specific details about the light power added in the figure notes.

In our revised manuscript, we added the following sentence after the discussion of film degradation conditions:

"Unless specified otherwise, all degradation experiments reported herein were conducted by exposing the samples to ambient air (relative humidity, RH = 38.0 ± 7.5%; temperature, T = 24.5 ± 0.9 °C; oxygen content, 20.9%), which is consistent with normal working conditions."

(please refer to this on Page 7 in our revised manuscript).

8. TOF-SIMS analysis is provided only for the stage 3 film. Could the authors include results for stages 1 and 2 for clearer comparison and better understanding?

The original manuscript lacked TOF-SIMS mappings for the other two stages, which limited the ability to make meaningful comparisons. In response to your suggestion, we have now supplemented the TOF-SIMS profiles for all three stages. Given an instrumentation update during this process, we unified the characterization of the three film stages to minimize experimental errors associated with equipment changes.

As shown in the figure, the ion distribution on the surface of the stage I film is uniform, whereas films from stages II and III exhibit Pb^+ aggregation. In these Pb^+-enriched regions, aggregation of I^- with FA^+ ions and a decrease in Sn^+ are also observed (the relatively low overall intensity of Sn^+ in the data may be due to the TOF-SIMS ion source's limited efficiency in bombarding and releasing Sn^+ ions, rather than an inherently low Sn^+ concentration). Although TOF-SIMS cannot determine the ions' valence states, small amounts of SnO^+ are detectable in the Pb^+-enriched regions, offering further insights into the superoxidation process.

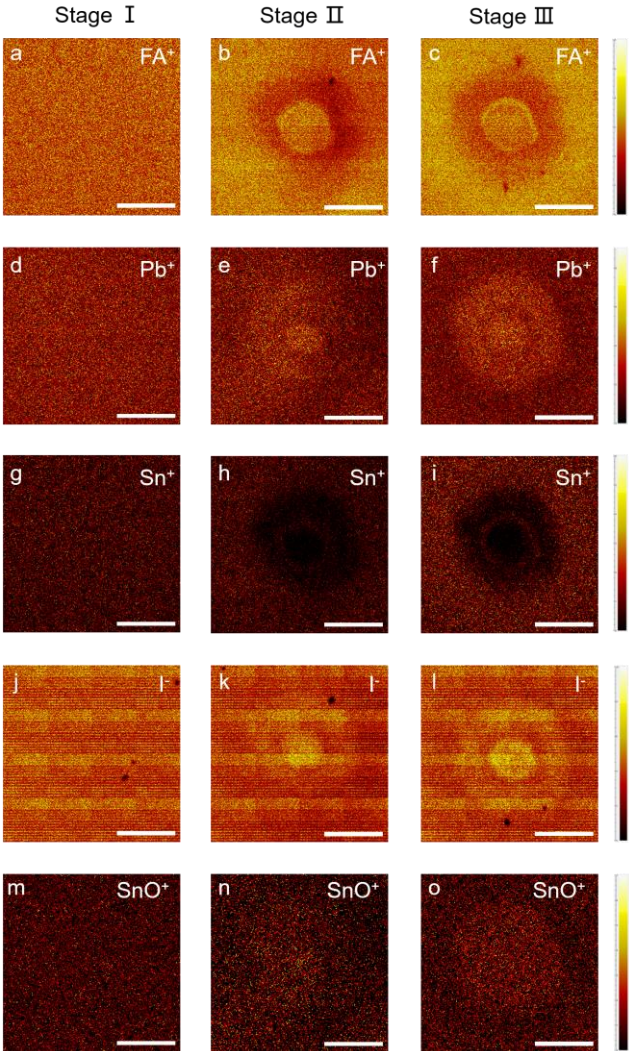

Fig. R25. The surfaced ions distribution of $FA_{0.7}MA_{0.3}Sn_{0.5}Pb_{0.5}I_3$ perovskite thin films in different stage (The scale bar is 100 μm). (a-c) TOF-SIMS mapping signals of FA^+ on the mixed $FA_{0.7}MA_{0.3}Sn_{0.5}Pb_{0.5}I_3$ perovskite films in different stage. (d-f) TOF-SIMS mapping signals of Pb^+ on the mixed $FA_{0.7}MA_{0.3}Sn_{0.5}Pb_{0.5}I_3$ perovskite films in different stage. (g-i) TOF-SIMS mapping signals of Sn^+ on the mixed $FA_{0.7}MA_{0.3}Sn_{0.5}Pb_{0.5}I_{33}$ perovskite films in different stage. (j-l) TOF-SIMS mapping signals of I^- on the mixed $FA_{0.7}MA_{0.3}Sn_{0.5}Pb_{0.5}I_3$ perovskite films in different stage. (m-o) TOF-SIMS mapping signals of SnO^+ on the mixed $FA_{0.7}MA_{0.3}Sn_{0.5}Pb_{0.5}I_3$ perovskite films in different stage.

9. The authors suggest that Sn^{2+} vacancies lead to triiodide ion formation, creating Pb-rich regions. However, triiodide formation is typically linked to halide mobility and redox processes involving I^- and I_2, not tin vacancies. Could the authors provide additional clarity on this concept? Also, [Page 12, Line number 225] how do the authors conclude that Sn^{2+} oxidation drives triiodide formation based on XRD data? Moreover, the authors suggest that molecular iodine escapes to the surface to form triiodide, but supporting evidence is lacking. Could they provide more data or references?

There is an error in the presentation of this section in the manuscript, and, as the reviewer noted,

there is no direct evidence to support that Sn^{2+} vacancies directly lead to triiodide ion formation. Additionally, there is ambiguity in the data presented alongside the XRD results. Jesús Sanchez – Diaz et al. investigated the evolution of the Sn^{2+}/Sn^{4+} ratio in $FASnI_3$ perovskite samples using XPS after different durations of light irradiation (Joule 2022, 6, 861-883). They found that the Sn^{2+}/Sn^{4+} ratio increased with illumination, peaking after three days of light soaking, suggesting that SnI_4 decomposes into SnI_2 and I_2 under light exposure.

Heavy metal complexes, such as metal halides, may exhibit metal-to-ligand or ligand-to-metal charge transfer transitions, which are especially pronounced when there is a significant difference in the electron-donating or withdrawing properties of the metal and ligand, respectively, as reflected in their redox potentials. These charge transfer processes are often associated with optical activity, indicating intramolecular photo-induced electronic transitions between the metallic center and the ligands. Notably, the Sn^{2+}/Sn^{4+} redox potential is +0.15 V, much lower than the typical chemical potential (or quasi-Fermi energy) difference of photoexcited carriers under illumination. Consequently, the observed decrease in Sn^{4+} content under illumination is likely due to the photo-induced reduction of Sn^{4+} to Sn^{2+} (Joule 2022, 6, 861-883).

In the manuscript, we analyzed the XPS data to suggest that the increase in triiodide ions in irradiated films is more pronounced relative to the Sn^{2+} oxidation rate. For validation, we included heated, aged films for comparison, confirming a faster increase of iodide ions in irradiated films. This insight could provide a basis for improving tin-lead perovskite thin films. Additional literature on the escape of molecular iodine to the surface, leading to triiodide formation, supports this mechanism (Energy Environ. Sci. 2022, 15, 5274; Angew. Chem. Int. Ed. 2015, 54, 8208-8212).

To clarify that more iodide ions are generated under irradiation, we have revised the manuscript accordingly:

" It is noteworthy that a distinct inconsistency exists between the changes in film surface elements during the reconstruction process and those following thermally induced degradation (under comparable temperature and duration conditions). The surface of thermally degraded thin films show significantly fewer triiodide ions and a higher Sn^{4+} concentration compared to irradiated films (Supplementary Fig. 20). The XPS profiles of oxygen across different stages further reveal that irradiation-induced degradation leads to more pronounced oxidation of the film than heating (Supplementary Fig. 21a, b). This indicates an accelerated generation of iodine vacancies during irradiation-induced superoxide degradation. Additionally, the rapid increase in the atomic ratio of lead ions to iodide ions during irradiation-induced phase reconstruction corroborates the formation of Pb-rich regions (Supplementary Fig. 21c)."

(please refer to this on Page 12 in our revised manuscript).

Reviewer 3 (Remarks to the Author):

Comments:

The referees' comments and questions have been thoroughly addressed with additional experiments and discussions. I have one minor issue remaining: FA_2SnI_6 is classified as a type of perovskite structure, specifically a vacancy-ordered double perovskite. Therefore, please revise the statement, 'it readily transforms into the FA_2SnI_6 non-perovskite structure. Unlike the perovskite structure, the $[SnI_6]^{4-}$ units in the FA_2SnI_6 structure do not connect through the iodide ions, but form a face-centered cubic arrangement with the A-site ions occupying the interstitials,' to accurately reflect this classification.

We sincerely thank the reviewer for their valuable comments and suggestions, which have greatly contributed to enhancing our interpretation of this work.

These are major issues:

FA_2SnI_6 is classified as a type of perovskite structure, specifically a vacancy-ordered double perovskite. Therefore, please revise the statement, 'it readily transforms into the FA_2SnI_6 non-perovskite structure. Unlike the perovskite structure, the $[SnI_6]^{4-}$ units in the FA_2SnI_6 structure do not connect through the iodide ions, but form a face-centered cubic arrangement with the A-site ions occupying the interstitials,' to accurately reflect this classification.

We fully agree that FA_2SnI_6 should be classified as a perovskite structure simply because its structure is inconsistent with conventional perovskite optically active black phases. What we are communicating to in the manuscript is the formation of a differently structured perovskite phase that differs from our common perovskite octahedral cell. The misrepresentation of the classification of crystal structures in the manuscript may be misleading.

To clarify, we have revised the statement in the manuscript as follows:

"it readily transforms into the FA_2SnI_6 structure. Unlike the common perovskite structure, the $[SnI_6]^{4-}$ units in the FA_2SnI_6 structure do not connect through the iodide ions, but form a face-centered cubic arrangement with the A-site ions occupying the interstitials."
(please refer to this on Page 16 in our revised manuscript).
